# A prospective trial of treatment de-escalation following neoadjuvant paclitaxel/trastuzumab/pertuzumab in HER2-positive breast cancer

**DOI:** 10.1038/s41523-022-00429-7

**Published:** 2022-05-10

**Authors:** Adrienne G. Waks, Neelam V. Desai, Tianyu Li, Philip D. Poorvu, Ann H. Partridge, Natalie Sinclair, Laura M. Spring, Meredith Faggen, Michael Constantine, Otto Metzger, Jillian Alberti, Julia Deane, Shoshana M. Rosenberg, Elizabeth Frank, Sara M. Tolaney, Ian E. Krop, Nadine M. Tung, Nabihah Tayob, Tari A. King, Elizabeth A. Mittendorf, Eric P. Winer

**Affiliations:** 1 Medical Oncology, Dana-Farber Cancer Institute, Boston, MA USA; 2 Breast Oncology Program, Dana-Farber/Brigham and Women’s Cancer Center, Boston, MA USA; 3 Harvard Medical School, Boston, MA USA; 4 Medical Oncology, Beth Israel Deaconess Medical Center, Boston, MA USA; 5 Department of Data Sciences, Dana-Farber Cancer Institute, Boston, MA USA; 6 Hematology/Oncology, Dana-Farber/Brigham and Women’s Cancer Center at Milford, Milford, MA USA; 7 Hematology/Oncology, Massachusetts General Hospital, Boston, MA USA; 8 Hematology/Oncology, Dana-Farber/Brigham and Women’s Cancer Center at South Shore Hospital, South Weymouth, MA USA; 9 Division of Breast Surgery, Department of Surgery, Brigham and Women’s Hospital, Boston, MA USA; 10 Present Address: Clinical Affairs, TransMedics, Inc, Andover, MA USA; 11 Present Address: Department of Population Health Sciences, Weill Cornell Medicine, New York, NY USA; 12 Present Address: Yale Cancer Center, New Haven, CT USA

**Keywords:** Breast cancer, Breast cancer

## Abstract

De-escalating adjuvant therapy following pathologic complete response (pCR) to an abbreviated neoadjuvant regimen in human epidermal growth factor receptor 2-positive (HER2+) breast cancer is the focus of international research efforts. However, the feasibility of this approach and its appeal to patients and providers had not been formally investigated. We aimed to assess adherence to de-escalated adjuvant antibody doublet therapy (trastuzumab and pertuzumab [HP], without chemotherapy) among patients with pCR following neoadjuvant paclitaxel/HP (THP). In this single-arm prospective trial, patients with treatment-naïve stage II-III HER2+ breast cancer received neoadjuvant weekly paclitaxel ×12 and HP every 3 weeks ×4. The primary endpoint was receipt of adjuvant non-HER2-directed cytotoxic chemotherapy. Ninety-eight patients received ≥1 dose of THP on study. Patients had median age of 50 years, 86% had stage II tumors, and 34% were hormone receptor-negative. Five patients had incomplete clinical response following THP and received doxorubicin and cyclophosphamide before surgery; they were classified as non-pCR and censored from further analyses. The overall pCR rate was 56.7%. Among patients with pCR, the adherence rate to de-escalated antibody-only therapy (HP) was 98.2% (95% CI 90.3–100.0%), and the primary feasibility endpoint was reached. The majority of patients felt positive or neutral about their adjuvant treatment plans. With brief follow-up (median 19.1 months), there were no breast cancer recurrences. De-escalation of adjuvant chemotherapy among patients who experience pCR in early-stage HER2+ breast cancer is a practicable approach for both patients and physicians. Planned and ongoing prospective trials will determine the long-term efficacy of this approach.

**Trial registration** clinicaltrials.gov, NCT03716180, https://clinicaltrials.gov/ct2/show/NCT03716180.

## Introduction

Modern treatment regimens for human epidermal growth factor receptor 2-positive (HER2+) breast cancer produce favorable long-term outcomes in the vast majority of patients with non-metastatic disease. The APHINITY trial demonstrated 3-year invasive disease-free survival (DFS) of 92% among node-positive early-stage HER2+ breast cancer patients treated with trastuzumab (H) and pertuzumab (P) plus adjuvant chemotherapy^1^. However, current standard-of-care neo/adjuvant regimens for stage II-III HER2+ breast cancer involve 2–3 chemotherapy agents plus HER2-directed therapy^2^, and these regimens are associated with both serious and burdensome short- and long-term toxicities^3^. It is of great interest to determine if a subset of patients with anatomic stage II-III HER2+ breast cancer can be adequately treated with curative intent using less toxic therapy.

Pathologic complete response (pCR) at surgery following neoadjuvant therapy is a strong favorable prognostic biomarker in all subtypes of breast cancer, including HER2+ breast cancer treated with standard modern regimens incorporating HER2-targeted therapy^4–6^. pCR is associated with an excellent long-term outcome and may identify patients who are prime candidates for de-escalated adjuvant treatment. Preliminary data indicate that pCR correlates with excellent long-term outcomes in HER2+ breast cancer even when the neoadjuvant regimen is chemotherapy-sparing or otherwise non-standard^7,8^. The CompassHER2-pCR trial (NCT04266249) is ongoing and will determine recurrence-free survival among patients with HER2+ breast cancer who receive an abbreviated neoadjuvant regimen and experience pCR, then omit additional standard cytotoxic chemotherapy.

Patients’ and providers’ acceptance of a pCR-based de-escalated treatment approach has not been formally investigated. One recent survey found that 43% of breast cancer patients were not interested in clinical trials investigating chemotherapy de-escalation, with fear of cancer recurrence and fear of regret being the most commonly cited reasons for concern^9^. Understanding concerns and preferences around this paradigm will be important for optimizing communication with patients about the new potential strategy and encouraging its uptake among appropriate patients.

The goal of this trial (DAPHNe: De-escalation to Adjuvant antibodies Post-pCR to Neoadjuvant THP) was to assess the feasibility of de-escalating therapy from a multi-agent to a single-agent chemotherapy backbone plus HP in select patients with anatomic stage II-III HER2+ breast cancer, based on pCR as a prognostic biomarker. All patients were planned to receive neoadjuvant paclitaxel-HP (THP), and patients who experienced pCR were recommended to receive adjuvant HP only, without further adjuvant cytotoxic chemotherapy. The primary objective was to assess adherence to the protocol-specified de-escalated adjuvant regimen (HP only) among patients with pCR. Post-operative patient questionnaires were administered to all patients and physician rationales were reviewed in the medical record to explore patient and provider attitudes in adjuvant therapy decision-making.

## Results

### Patient characteristics

**Fig. 1:**
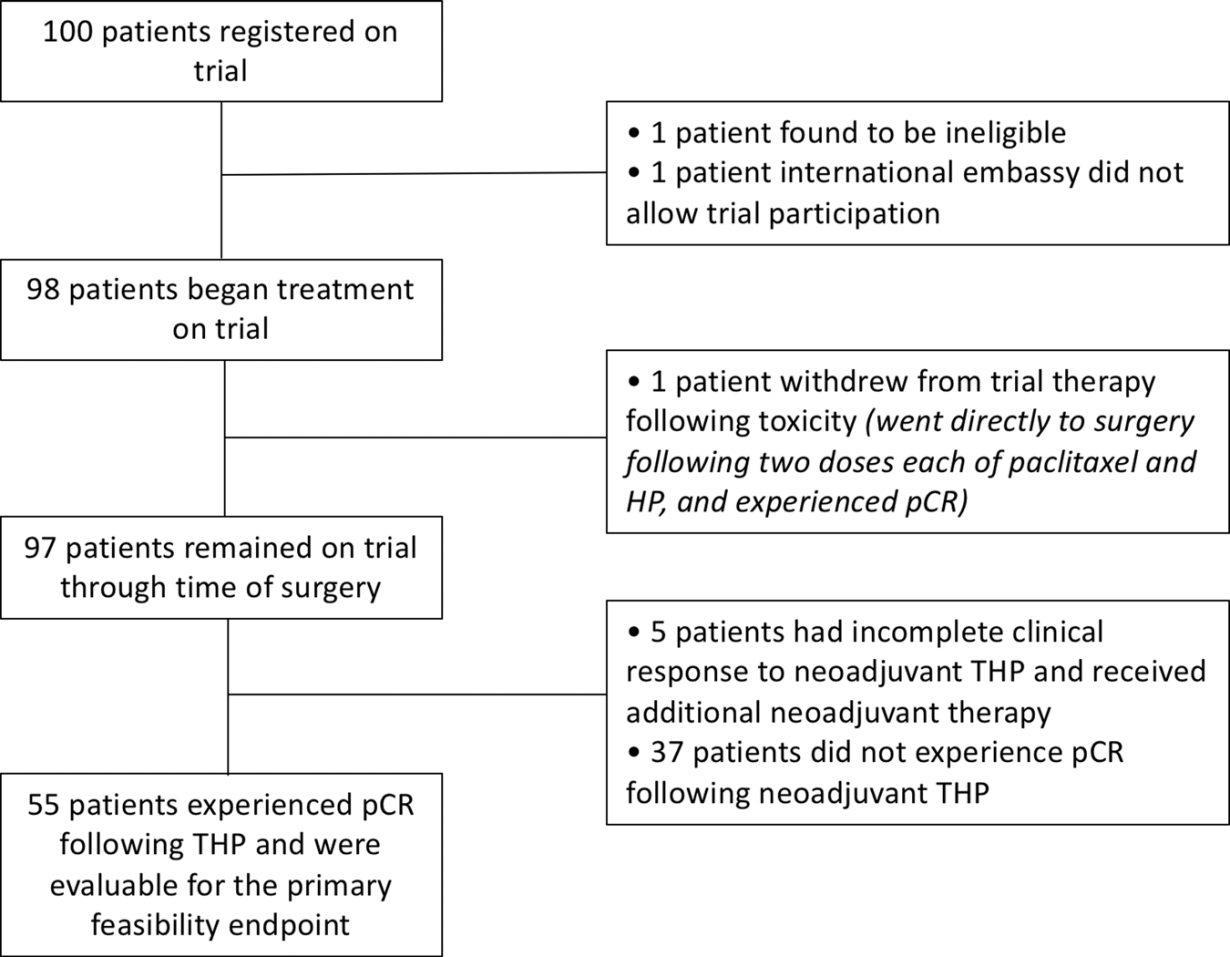
Trial flow diagram. pCR pathologic complete response, THP paclitaxel/trastuzumab/pertuzumab.

Table 1 summarizes patient and tumor characteristics for 98 patients who began treatment on trial. The large majority of patients had clinical anatomic stage II disease (85.7%), and approximately one-third of patients had hormone receptor-negative (HR-) tumors (33.7%). Supplementary Table 1 shows all neoadjuvant treatments received: 84.7% of patients completed all 12 doses of neoadjuvant paclitaxel, and 99%/98% of patients completed at least 4 doses of neoadjuvant H/P, respectively. One patient withdrew early for toxicity and is not included in subsequent analyses. Five patients (5.1%) had obvious residual disease at the completion of THP and received preoperative doxorubicin and cyclophosphamide (AC); all other patients underwent surgery following THP (Fig. 1).

**Table 1:** Patient and tumor characteristics.

Characteristic	No. of patients (%) (*N* = 98)
Age, years	
Median (range)	49.5 (24–78)
Sex	
Female	97 (99%)
Male	1 (1%)
Race	
White	82 (83.7%)
Black	5 (5.1%)
Asian	7 (7.1%)
Other	4 (4.1%)
Ethnicity	
Hispanic or Latino	5 (5.1%)
Non-Hispanic	89 (90.8%)
Unknown	4 (4.1%)
ECOG PS at baseline	
0	93 (94.9%)
1	4 (4.1%)
Unknown	1 (1%)
Stage at initial diagnosis	
II	84 (85.7%)
III	14 (14.3%)
T status	
Tx	1 (1%)
T1	17 (17.3%)
T2	72 (73.5%)
T3	8 (8.2%)
T4	0 (0%)
N status	
N0	65 (66.3%)
N1	30 (30.6%)
N2	2 (2%)
N3	1 (1%)
Hormone receptor status	
ER+/PR+	45 (45.9%)
ER+/PR−	18 (18.4%)
ER−/PR+	2 (2%)
ER−/PR−	33 (33.7%)
HER2 status	
Positive	98 (100%)
Size of breast tumor by physical exam (cm)	
Median (range)	3 (0–6)
Breast surgery	
Lumpectomy	54 (55.1%)
Mastectomy	44 (44.9%)

*ECOG PS* Eastern Cooperative Oncology Group Performance Status, *ER* estrogen receptor, *HER2* human epidermal growth factor receptor 2, *PR* progesterone receptor.

### Neoadjuvant therapy responses and adjuvant therapy received

**Fig. 2:**
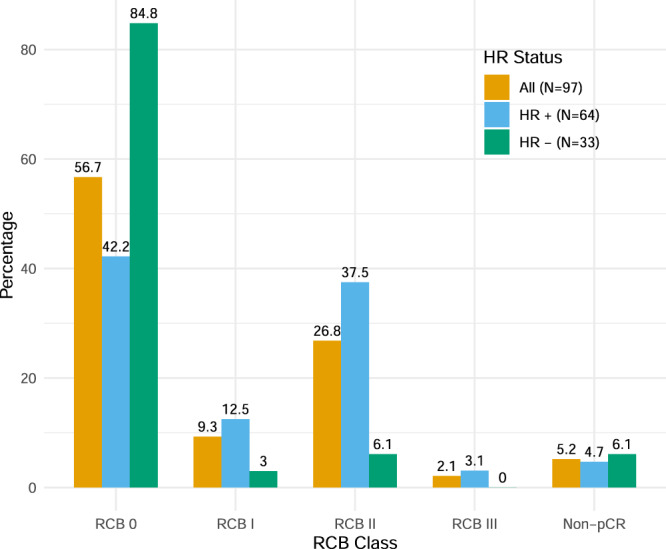
Pathologic response results. Non-pCR indicates patients who received additional neoadjuvant chemotherapy following paclitaxel/trastuzumab/pertuzumab. HR hormone receptor, pCR pathologic complete response, RCB residual cancer burden.

The overall pCR rate was 56.7%, with residual cancer burden (RCB) I, II, and III responses in 9.3%, 26.8%, and 2.1% of patients, respectively. The pCR rate was 42.2% for hormone receptor-positive (HR+) patients, and 84.8% for HR- patients (Fig. 2). Table 2 shows all adjuvant therapies received by RCB category. Among patients who experienced pCR following neoadjuvant THP (*N* = 55), the rate of adherence to de-escalated antibody-only therapy (HP) was 98.2% (95% confidence interval [CI] 90.3–100.0%). Thus, the trial met its primary feasibility endpoint (*p* value from binomial test: <0.001). Among the remaining 37 patients with non-pCR responses to neoadjuvant THP, 16 patients received adjuvant chemotherapy (AC) [*N* = 14]; cyclophosphamide alone [*N* = 2], and 21 patients did not receive adjuvant chemotherapy (19 of whom received adjuvant T-DM1). Overall, 29/37 patients who did not have a pCR (78%) received at least one dose of adjuvant T-DM1. 84.4% of patients with HR+ disease (54/64 patients) initiated adjuvant hormonal therapy.

**Table 2:** All non-hormonal adjuvant systemic therapies received.

pCR status	Adjuvant cytotoxic chemotherapy received		Adjuvant antibody therapy received	
	Regimen	No. patients (%)	Regimen	No. patients (%)
pCR aka RCB 0 (*N* = 55)	AC ×4 cycles	1 (1.8%) (95% CI 0.05–9.7%)	H (trastuzumab)	1 (100%)
			P (pertuzumab)	1 (100%)
			T-DM1	0
	None	54 (98.2%) (95% CI 90.3–100%)	H	54 (100%)
			P	50 (92.6%)
			T-DM1	0
RCB I (*N* = 9)	AC ×4 cycles	1 (11.1%)	H	0
			P	0
			T-DM1	1 (100%)
	None	8 (88.9%)	H	5 (62.5%)
			P	4 (50%)
			T-DM1	7 (87.5%)
RCB II (*N* = 26)	AC ×4 cycles^a^	12 (46.2%)	H	6 (50%)
			P	6 (50%)
			T-DM1	7 (58.3%)
	Cyclophosphamide x4 cycles	2 (7.7%)	H	1 (50%)
			P	0
			T-DM1	1 (50%)
	None	12 (46.2%)	H	5 (41.7%)
			P	2 (16.7%)
			T-DM1	11 (91.7%)
RCB III (*N* = 2)	AC x4 cycles	1 (50%)	H	0
			P	0
			T-DM1	1 (100%)
	None	1 (50%)	H	0
			P	0
			T-DM1	1 (100%)

Patients who received neoadjuvant AC are not included in this table.

*AC* doxorubicin + cyclophosphamide, *CI* confidence interval, *pCR* pathologic complete response, *RCB* residual cancer burden.

^a^In one patient 4 cycles of AC were planned, but stopped early (after 2 cycles) for toxicity.

With 19.1 months of median follow-up, there were no breast cancer recurrences, new primary breast cancers, or deaths. One patient was diagnosed with metastatic small cell carcinoma of likely pancreatic primary.

### Patient and provider attitudes toward chemotherapy and de-escalation

**Fig. 3:**
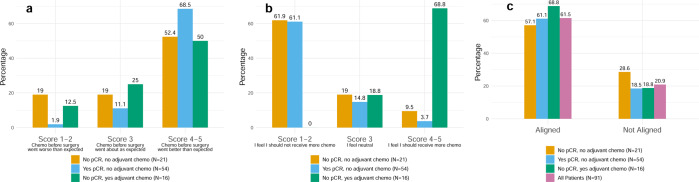
Patient responses to questionnaire regarding neoadjuvant and adjuvant chemotherapy. **a** Patient reflections on neoadjuvant chemotherapy. Specifically, this panel shows responses to the question, “How would you describe your experience with the chemotherapy you received before surgery”? **b** Patient perspectives on adjuvant chemotherapy. Specifically, this panel shows responses to the question, “How strongly do you feel that you should or should not receive more chemotherapy after your surgery?” Patients who selected score 1–2 (“I feel I should not receive more chemo”) or score 3 (“I feel neutral”) and did not have adjuvant chemotherapy planned were classified as feeling positive/neutral about their planned adjuvant regimen. Patients who selected score 4–5 (“I feel I should receive more chemo”) or score 3 (“I feel neutral”) and had adjuvant chemotherapy planned were classified as feeling positive/neutral about their planned adjuvant regimen. **c** Patient-physician alignment in planning for adjuvant chemotherapy, as rated by patients. “Aligned” was defined as: patient gave a response of 1 or 2 on question describing patient’s feeling about adjuvant chemotherapy and question describing treating physician’s feeling about adjuvant chemotherapy; or patient gave a response of 3 on both questions; or patient gave a response of 4 or 5 on both questions. “Not aligned” was defined as everything else. pCR pathologic complete response.

Post-operative questionnaires were administered to 100% of patients to query patients’ experiences with neoadjuvant chemotherapy, attitudes toward additional adjuvant chemotherapy, and perceived alignment with their treating physician about the need for additional adjuvant chemotherapy. Response data are shown according to the following patient categories: no pCR and did not receive adjuvant chemotherapy; yes pCR and did not receive adjuvant chemotherapy; no pCR and did receive adjuvant chemotherapy (Fig. 3, associated data in Supplementary Table 2). Non-de-escalator patient data (yes pCR and did receive adjuvant chemotherapy) are included only in the supplement as only one patient was in this category. There was a 10–20% non-response rate for all questions, with approximately equivalent non-response rates across patient categories. In all patient categories, ≥50% of patients felt that preoperative chemotherapy went better than expected (score 4–5), and patients who experienced pCR were numerically most likely to report a better than expected preoperative chemotherapy experience (Fig. 3a).

The large majority of patients felt positive or neutral about their adjuvant treatment plans, regardless of whether they planned to omit or receive additional chemotherapy such as AC. Among patients who did not plan to receive adjuvant chemotherapy, though most felt positive or neutral about that decision (score 1–3), a small minority (3.7% who had experienced pCR, and 9.5% who had not experienced pCR) reported feeling that they “should” receive more chemotherapy (score 4–5)—despite not planning to receive more. Among patients who planned to receive adjuvant chemotherapy after not experiencing pCR, 100% felt positive or neutral about that decision (score 3–5; Fig. 3b). 61.5% of patients overall felt aligned with their treating physician about adjuvant chemotherapy decisions while 20.9% of patients felt non-aligned (with 17.6% missing data for this two-question analysis; Fig. 3c).

Patient and physician rationale for administering or omitting adjuvant chemotherapy were also explored through questionnaires and medical record review, with opportunity for prespecified or free-text responses. For patients who did not achieve pCR and did not receive adjuvant chemotherapy such as AC (*N* = 21), the most common reason cited for omitting adjuvant chemotherapy was plan for adjuvant T-DM1 (cited by 14 patients and 17 physicians), and the second most common reason was a good response to neoadjuvant chemotherapy (cited by 8 patients and 7 physicians; Supplementary Table 3). Themes that emerged from free-text responses were grouped by omission or receipt of adjuvant chemotherapy such as AC after either pCR or lack of pCR, respectively. Among patients with pCR, themes related to omission of adjuvant chemotherapy included (1) following physician advice, (2) emphasizing the importance of pCR found at surgery, and (3) worry about chemotherapy toxicity. Among patients without pCR, themes related to receipt of adjuvant chemotherapy included (1) high disease risk, and (2) following the most evidence-based treatment approach regardless of side effects. Supplementary Table 4 contains all patient-written responses.

## Discussion

This trial demonstrated the feasibility of de-escalating from multi-agent to single-agent cytotoxic chemotherapy in combination with dual anti-HER2 antibody therapy in patients with pCR after neoadjuvant THP. In this cohort, where the majority of patients had clinical anatomic stage II disease, just over half (56.7%) of patients experienced pCR. With brief follow-up in this small cohort, no breast cancer recurrences were seen. If ongoing larger trials (e.g. CompassHER2-pCR) demonstrate favorable long-term efficacy associated with this treatment approach, then the majority of patients with anatomic stage II-III HER2+ breast cancer may be able to avoid the substantial toxicities associated with standard combined chemotherapy regimens.

The overall pCR rate of 56.7% seen in this trial is comparable to pCR rates previously reported in other cohorts of stage II-III HER2+ breast cancer treated with various chemo-plus-HP regimens. In the NeoSphere trial, 4 cycles of docetaxel/HP produced a pCR rate (ypT0/isN0) of 39.3% (*N* = 107)^10^; in the KRISTINE trial, 6 cycles of docetaxel/carboplatin/HP (TCHP) or T-DM1/P produced pCR rates (ypT0/isN0) of 55.7% (*N* = 221) and 44.4% (*N* = 223), respectively^11^; and in the TRYPHAENA trial, 6 cycles of 5-fluorouracil/epirubicin/cyclophosphamide-docetaxel/HP (FEC-THP) or TCHP produced pCR rates (ypT0N0) of 45.3% (*N* = 75) and 51.9% (*N* = 76)^12^. As in all other cohorts of HER2+ breast cancer treated with neoadjuvant therapy, pCR was significantly more likely for those with HR- tumors compared to HR+ tumors. Though patients with HR+/HER2+ tumors are less likely to experience pCR, pCR carries less prognostic importance in this subset compared to HR−/HER2+ tumors, presumably due to the long-term benefits of adjuvant endocrine therapy^4^.

The DAPHNe trial represents a formal assessment of feasibility for a pCR-based de-escalation approach to therapy in HER2+ breast cancer. HER2+ breast cancer is well suited to systemic therapy de-escalation due to the development of relatively low-toxicity, high-efficacy targeted therapies beginning with the U.S. Food and Drug Administration approval of adjuvant trastuzumab in 2006. The use of pCR as a patient-level surrogate for de-escalation candidacy^13^ is supported by the excellent outcomes for patients with HER2+ breast cancer and pCR regardless of neoadjuvant regimen. In the KRISTINE trial, patients who experienced pCR after neoadjuvant T-DM1 plus P had 96.7% 3-year invasive DFS (despite only 9.1% receiving adjuvant chemotherapy), and the I-SPY2 trial reported a 93–97% 3-year event-free survival for patients with pCR following varied neoadjuvant regimens for stage II–III HER2+ breast cancer, including investigational regimens^7,8^. Therefore, prospectively evaluating the efficacy of pCR-based de-escalation in HER2+ breast cancer is essential. The ongoing CompassHER2-pCR trial will enroll 1250 patients with stage II-IIIA HER2+ breast cancer and determine recurrence-free survival with a treatment approach nearly identical to the DAPHNe trial. A similarly structured European trial (DECRESCENDO) is planned for 1065 patients with ER−/HER2+ stage I–II breast cancer (tumor size 15–50 mm)^13^. Patients with stage III disease likely will not be well-represented in these trials (with stage IIIB/C entirely excluded), as we observed in the DAPHNe trial: only 14 stage III patients participated, though all non-inflammatory stage III tumors were eligible.

For patients without pCR on DAPHNe, several themes in adjuvant therapy administration are notable. While all adjuvant therapy was administered off-trial and therefore up to clinician discretion, the protocol specifically recommended adjuvant T-DM1 in all patients with residual disease, and additional chemotherapy in patients with RCB III residual disease at surgery or otherwise high risk. At least one dose of adjuvant T-DM1 was administered in 78% of patients with residual disease. Adjuvant chemotherapy was omitted in most patients with RCB I and approximately half of patients with RCB II residual disease at surgery. This reflects the fact that long-term disease outcomes are strongly associated with RCB categorization, with increasing (less favorable) RCB score predicting worse relapse-free survival^14^. Though ongoing and planned trials will inform adjuvant therapy decisions for patients with pCR, it is unlikely that prospective trials will be performed to determine the optimal adjuvant regimen for patients with good but non-pCR response to THP. Accordingly, these decisions will continue to be made on an individualized basis, as was the case in the DAPHNe cohort. For patients with significant residual disease at surgery, the use of adjuvant anthracycline-based chemotherapy (e.g. AC) will remain an important consideration. If used, AC should be administered in a dose-dense fashion (every 2 weeks) as this schedule was associated with improved 10-year breast cancer outcomes in a large meta-analysis^15^.

Patients’ and treating physicians’ reports offer insights into the reasoning and confidence level underlying adjuvant therapy decisions. Most patients reported feeling positive or neutral about their adjuvant regimen, regardless of whether further chemotherapy was planned or not. However, there were modestly numerically higher rates of positive/neutral feelings toward adjuvant therapy plan and slightly higher rates of perceived patient–physician alignment among patients who were planned for adjuvant chemotherapy, potentially suggestive of a higher level of ambivalence among patients who did not plan adjuvant chemotherapy. This underscores the importance of thorough communication about the risks and benefits of de-escalation as well as acknowledgment of the potential for psychological discomfort. Conversely, the fact that planned use of T-DM1 was the top reason cited for de-escalation among patients without pCR highlights patients’ and physicians’ relative comfort with the substitution of a more targeted, less toxic agent for a standard combination chemotherapy regimen—and likely reflects the fact that de-escalation of toxic therapy is easier to consider when something alternative is offered in its place.

Our trial data have several limitations. Most patients were enrolled at a single tertiary academic cancer center (DFCI) where providers already had familiarity with adjuvant de-escalation trials in HER2+ breast cancer based on participation in prior protocols, which may have impacted their comfort level with this approach and experience presenting it to prospective participants. Offsetting this, approximately one in three enrolled patients were from other centers including community satellite practices. While even large trials of a similar de-escalation approach (CompassHER2-pCR and DECRESCENDO) will be potentially subject to the same enrollment biases related to provider experience/comfort, we expect that given larger sample sizes and broad recruitment base, those efficacy results will be generalizable for community uptake. The patient questionnaires used to assess adjuvant therapy decision-making were developed by the study team and not previously validated. Finally, we did not gather data on the number or characteristics of patients who declined to participate in the trial, though the rapidity of accrual (>7 patients/month) highlights broad patient interest.

The DAPHNe trial formally assessed patients’ acceptance of de-escalated adjuvant therapy in clinical anatomic stage II-III HER2+ breast cancer. Given the landscape of ongoing trials, we anticipate that this may be a major emerging treatment paradigm in non-metastatic HER2+ breast cancer. While larger cohorts will be instrumental in establishing the long-term efficacy of this treatment strategy, this trial was unique in its focus on patient attitudes toward chemotherapy, patient-physician alignment with respect to adjuvant chemotherapy, and patients’ sources of reassurance and reservation about adjuvant therapy de-escalation within this specific patient population. We must continue to evaluate patients’ and physicians’ perspectives on de-escalation in order to optimize communication, facilitate informed decision-making, and ultimately encourage uptake of this evolving treatment approach that seeks to minimize toxicity without compromising benefit in the appropriate contexts.

## Methods

### Patient population

Eligible patients had clinical anatomic stage II-III HER2+ invasive breast cancer. HER2 positivity was defined according to 2018 American Society of Clinical Oncology/College of American Pathologists guidelines^16^. Patients could have any menopausal or hormone receptor status, and were required to have performance status ≤1 and adequate organ function at baseline. Patients with baseline cardiac ejection fraction <50% or significant peripheral neuropathy (grade ≥ 2 by common terminology criteria for adverse events v4.0) were excluded. All patients provided written informed consent and the study was carried out in accordance with the Declaration of Helsinki.

### Treatment protocol

This was a single-arm prospective trial that enrolled patients from November 2018 to January 2020 at Dana-Farber/Harvard Cancer Center (DF/HCC; composed of Dana-Farber Cancer Institute [DFCI], Massachusetts General Hospital, and Beth Israel Deaconess Medical Center) and affiliated community satellite practices. All patients were assigned to receive preoperative paclitaxel (T; 80 mg/m^2^ weekly for 12 weeks), trastuzumab (H; loading dose 8 mg/kg, subsequent doses 6 mg/kg, every 3 weeks for 4 cycles), and pertuzumab (P; loading dose 840 mg, subsequent doses 420 mg, every 3 weeks for 4 cycles) prior to breast surgery. Up to two additional cycles of HP were allowed in cases of surgical delay. Patients with obvious residual disease at completion of THP were allowed to receive additional neoadjuvant therapy at investigator discretion; 4 cycles of AC was the recommended regimen. Pathologic response to neoadjuvant therapy was quantified at surgery according to RCB score;^17^ pCR was defined as RCB 0 (ypT0/isN0). Patients with pCR were suggested to complete one year of adjuvant HP, without additional cytotoxic chemotherapy. In patients without pCR, adjuvant systemic therapy was per investigator discretion, with 14 cycles of trastuzumab emtansine (T-DM1) recommended for all patients (per protocol amendment following presentation of the KATHERINE trial data^18^) and 4 cycles of AC recommended in patients with significant residual disease. Post-operative hormonal therapy was administered per investigator discretion. All patients were followed for disease outcomes post-operatively. All trial procedures were approved by the DF/HCC institutional review board. The full protocol is included in Supplementary Material.

### Assessment of adjuvant therapy decision-making

After completion of final breast surgery, patients belonged to one of four adjuvant therapy designations based on their pCR status and receipt of adjuvant cytotoxic chemotherapy: (1) non-de-escalator: patients with pCR who received adjuvant cytotoxic chemotherapy; (2) patients without pCR who did not receive adjuvant cytotoxic chemotherapy; (3) patients without pCR who received adjuvant cytotoxic chemotherapy; and (4) patients with pCR who did not receive adjuvant cytotoxic chemotherapy. T-DM1 was not considered cytotoxic chemotherapy for purposes of this categorization. A 4-item paper-based questionnaire, developed by the study team, regarding preferences and rationale for receipt/non-receipt of adjuvant cytotoxic chemotherapy was administered post-operatively and prior to initiation of adjuvant systemic therapy to all patients. Prior to questionnaire administration, the final plan for adjuvant cytotoxic chemotherapy administration (yes/no and regimen) was signed off on by the treating physician. Treating physician rationale for administration/non-administration of adjuvant cytotoxic chemotherapy was recorded by two independent physician reviewers based on review of progress notes in the medical record. Discordant opinions were jointly discussed by the two reviewers and consensus was reached. Questionnaires and standardized medical record review forms are included in Supplementary Material.

### Statistical methods

The primary objective was to assess adherence to protocol-specified antibody doublet therapy (HP only) in the adjuvant setting among patients with pCR following neoadjuvant THP. The primary endpoint was receipt of adjuvant cytotoxic chemotherapy, assessed 3 months post-operatively. Among patients with pCR to THP, de-escalation would be deemed infeasible if the true rate of adherence to HP only was ≤80%. With a sample size of 100 patients, the study was designed to have > 90% power to reject the null if the true rate of adherence was ≥ 95% (binomial exact test; one-sided alpha = 0.05). Patients who progressed during neoadjuvant THP, withdrew consent to participate, received neoadjuvant therapy in addition to THP, or did not have pCR were not included in the primary analysis (prespecified). Secondary endpoints included event-free survival and overall survival. Patients who received additional non-THP neoadjuvant therapy were counted as non-pCR. Questionnaire and medical record review results for analysis of adjuvant therapy decision-making were summarized descriptively and patients who received additional neoadjuvant therapy following THP were not included in this analysis. SAS v9.4 was used for data analysis and R v4.0.2 was used to make figures.

### Reporting summary

Further information on research design is available in the Nature Research Reporting Summary linked to this article.

## Supplementary information

Supplementary Tables

Protocol

Questionnaire Packet

NPJ Reporting Summary

## Data Availability

The datasets generated during and/or analyzed during the current study are available from the corresponding author on reasonable request.
