# Supplementary material for: A prospective trial of treatment de-escalation following neoadjuvant paclitaxel/trastuzumab/pertuzumab in HER2-positive breast cancer
Source: NPJ Breast Cancer. 2022 May 10;8:63. doi: 10.1038/s41523-022-00429-7 (PMC9091255; doi:10.1038/s41523-022-00429-7)
Supplement: Supplementary file 1 — Supplementary Tables [file 41523_2022_429_MOESM1_ESM.pdf]

SUPPLEMENT

Supplementary Table 1. Neoadjuvant Treatment Received

Agent and # of doses received	No. of patients (N=98) (%)
**Paclitaxel**	
12 doses received*	83 (84.7%)
<12 doses received	15 (15.3%)
Any doses delayed	10 (10.2%)
Any doses reduced*	16 (16.3%)
**Trastuzumab**	
4 doses received (exactly)	80 (81.6%)
5 doses received (exactly)	12 (12.2%)
6 doses received (exactly)	5 (5.1%)
<4 doses received	1 (1%)
**Pertuzumab**	
4 doses received (exactly)	84 (85.7%)
5 doses received (exactly)	8 (8.2%)
6 doses received (exactly)	4 (4.1%)
<4 doses received	2 (2%)
**Additional (non-THP) neoadjuvant therapy given**	
Yes	5 (5.1%)
No	93 (94.9%)
**Additional neoadjuvant regimen administered**	
AC x 4 cycles	4 (4.1%)
Other (please list)**	1 (1%)

*One patient received 12 doses of nab-paclitaxel, some at reduced dose, following hypersensitivity reaction at cycle 1 day 1 of paclitaxel

**3 cycles of AC were given. 4 were planned but only 3 were completed due to toxicity.
AC: doxorubicin + cyclophosphamide, THP: paclitaxel/trastuzumab/pertuzumab

Supplementary Table 2:
Patient responses to questionnaire regarding neoadjuvant and adjuvant chemotherapy.

2A: Patient reflections on neoadjuvant chemotherapy—responses to the question "How would you describe your experience with the chemotherapy you received before surgery?"

Numeric response	No pCR, no adjuvant chemotherapy N=21	Yes pCR, no adjuvant chemotherapy N=54	No pCR, yes adjuvant chemotherapy N=16
**1 (chemo before surgery went worse than expected)**	2 (9.5%)	0 (0%)	1 (6.3%)
**2**	2 (9.5%)	1 (1.9%)	1 (6.3%)
**3 (chemo before surgery went about as expected)**	4 (19%)	6 (11.1%)	4 (25%)
**4**	1 (4.8%)	6 (11.1%)	3 (18.8%)
**5 (chemo before surgery went better than expected)**	10 (47.6%)	31 (57.4%)	5 (31.3%)
**Missing data for this question**	2 (9.5%)	10 (18.5%)	2 (12.5%)

2B: Patient perspectives on adjuvant chemotherapy—responses to the question “How strongly do you feel that you should or should not receive more chemotherapy after your surgery?”

Numeric response	No pCR, no adjuvant chemotherapy N=21	Yes pCR, no adjuvant chemotherapy N=54	No pCR, yes adjuvant chemotherapy N=16
1 (I feel I should not receive more chemo)	10 (47.6%)	30 (55.6%)	0 (0%)
2	3 (14.3%)	3 (5.6%)	0 (0%)
3 (I feel neutral)	4 (19%)	8 (14.8%)	3 (18.8%)
4	2 (9.5%)	0 (0%)	2 (12.5%)
5 (I feel I should receive more chemo)	0 (0%)	2 (3.7%)	9 (56.3%)
Missing data for this question	2 (9.5%)	11 (20.4%)	2 (12.5%)

2C: Patient-physician alignment in planning for adjuvant chemotherapy, as rated by patients.

Patient-MD alignment status	All patients overall N=91	No pCR, no adjuvant chemotherapy N=21	Yes pCR, no adjuvant chemotherapy N=54	No pCR, yes adjuvant chemotherapy N=16
**Aligned**	56 (61.5%)	12 (57.1%)	33 (61.1%)	11 (68.8%)
**Not aligned**	19 (20.9%)	6 (28.6%)	10 (18.5%)	3 (18.8%)
**Missing data for this analysis (i.e. missing data for question 1 or 2)**	16 (17.6%)	3 (14.3%)	11 (20.4%)	2 (12.5%)

“Aligned” was defined as: patient gave a response of 1 or 2 on question describing patient’s feeling about adjuvant chemotherapy *and* question 2 describing treating physician’s feeling about adjuvant chemotherapy; or patient gave a response of 3 on both questions; or patient gave a response of 4 or 5 on both questions. “Not aligned” was defined as everything else.
pCR: pathologic complete response

Supplementary Table 3:

Patient and treating physician reasons for not administering adjuvant chemotherapy in patients without pCR (N=21)

**Reasons for non-administration of adjuvant chemotherapy**	**No. of patients (%) Source: patient questionnaire**	**No. of patients (%) Source: physician reviewers**
Good response to neoadjuvant chemo	8 (38.1%)	7 (33.3%)
Plan for adjuvant T-DM1	14 (66.7%)	17 (81%)
Patient age	1 (4.8%)	1 (4.8%)
Patient comorbidity	0 (0%)	1 (4.8%)
Plan for hormonal therapy	5 (23.8%)	2 (9.5%)
Other* (write-in opportunity)	1 (4.8%)	N/A
No response provided to this question	4 (19%)	0 (0%)

Multiple responses were allowed to this question.

*One patient provided the write-in "Other" response: *"Double mastectomy – no node involvement."*

pCR: pathologic complete response

Supplementary Table 4: Free text rationales for adjuvant chemotherapy treatment decisions according to pCR status

**Rationales among patients with pCR (not planned for adjuvant chemotherapy)**
**Theme 1: Following physician advice**
My doctor mentioned a relevant 7-year study which made the reasoning even stronger.
Doctors said further chemo after surgery not needed per the pathology results.
**Theme 2: Emphasizing the importance of pCR found at surgery**
There is nothing left to shrink.
Complete pathological response.
The plan worked and killed off the cancer - no more chemo.
**Theme 3: Worry about chemotherapy toxicity**
In addition to a pCR (which guided my decision not to do AC) I was extremely wary of doing AC due to its cardio toxicity and potential for leukemia later in life.
**Rationales among patients without pCR (planned for adjuvant chemotherapy)**
**Theme 1: High disease risk**
Because my cancer is high risk and pathology scored my residual cancer burden = 2 ***(RCB II)***
I will because it is going to get rid of any cancer cells that are left. ***(RCB II)***
**Theme 2: Following the most evidence-based treatment regardless of side effects**
Pt wants to receive treatment with most research that she felt would treat her the best (even if it has more side effects/is more aggressive). ***(RCB II)***
I made a personal decision with guidance from my doctor to have more chemo…I personally want to do whatever it takes to get to the best possible outcome and this path has data to support good results. I also did not want to wonder or second guess my decision if I didn't get more chemo. The mental aspect of this played a huge part. I wasn't willing to risk not having more chemo without data to support that path. ***(RCB II)***
**Rationale among patient with pCR (planned for adjuvant chemotherapy)**
Even though all cancer gone my long term outcome could be better with more chemo

Responses provided to the optional write-in question, *"Other than the guidelines of the clinical trial, please list any other reasons that you will or will not be receiving more chemotherapy after surgery."* 58 patients completed at least one question on this questionnaire; 10 wrote in a response to this question. RCB score of the patient who provided the comment is included in parentheses for patients with non-pCR. The single patient who received adjuvant chemotherapy (AC) following pCR provided a free text response to the prompt *"The reason(s) I am going to receive more chemotherapy after my surgery is/are…"* which is included in the final line of the table.

AC: doxorubicin + cyclophosphamide, pCR, pathologic complete response, RCB: residual cancer burden
