# Supplementary material for: A prospective trial of treatment de-escalation following neoadjuvant paclitaxel/trastuzumab/pertuzumab in HER2-positive breast cancer
Source: NPJ Breast Cancer. 2022 May 10;8:63. doi: 10.1038/s41523-022-00429-7 (PMC9091255; doi:10.1038/s41523-022-00429-7)
Supplement: Supplementary file 2 — Protocol [file 41523_2022_429_MOESM2_ESM.pdf]

DF/HCC Protocol #: 18-394

TITLE: DAPHNe: De-escalation to adjuvant antibodies post-pCR to neoadjuvant THP (paclitaxel/trastuzumab/pertuzumab)—a pilot study in HER2-positive breast cancer

Coordinating Center: Dana-Farber Cancer Institute

Project Manager: Dana-Farber Cancer Institute
Phone: 617-582-8935
Fax: 617-632-5152
CTOPM@dfci.harvard.edu

Principal Investigator (PI): Adrienne Gropper Waks, MD
Dana-Farber Cancer Institute
450 Brookline Ave, Smith 353
Boston, MA 02215
Tel: 617-632-3779
Fax: 617-632-5822
awaks@partners.org

Other Investigators: Eric P. Winer, MD
Dana-Farber Cancer Institute
450 Brookline Avenue, Yawkey 1244
Boston, MA 02215
Tel: 617-632-6876
Fax: 617-632-1930
Eric_Winer@dfci.harvard.edu

Nadine Tung, MD
Beth Israel Deaconess Medical Center
330 Brookline Avenue, Shapiro 9
Boston, MA 02215
Tel: 617-667-7081
Fax: 617-975-5665
Email: ntung@bidmc.harvard.edu

Neelam Desai, MD
Beth Israel Deaconess Medical Center
330 Brookline Avenue, Shapiro 9
Boston, MA 02215
Tel: 617-667-7081
Fax: 617-975-5665
Email: ndesai@bidmc.harvard.edu

Elizabeth Mittendorf, MD, PhD
Dana-Farber Cancer Institute

450 Brookline Avenue
Boston, MA 02215
Tel: 617-632-2495
Fax: 617-632-3891
Email: emittendorf@bwh.harvard.edu

Tari King, MD
Dana-Farber Cancer Institute
450 Brookline Avenue
Boston, MA 02215
Tel: 617-632-2495
Fax: 617-632-3891
Email: tking7@bwh.harvard.edu

Shoshana Rosenberg, PhD
Dana-Farber Cancer Institute
44 Binney St
Boston, MA 02115
Phone: 617-582-7593
Fax: 617-632-5690
Email: shoshana_rosenberg@dfci.harvard.edu

Ann Partridge, MD
Dana-Farber Cancer Institute
450 Brookline Avenue, Yawkey 1244
Boston, MA 02215
Tel: 617-632-3800
Fax: 617-632-1930
Email: ann_partridge@dfci.harvard.edu

NCI-Supplied Agent(s): N/A
Other Agent(s): Paclitaxel (commercial), trastuzumab (commercial), pertuzumab (commercial)

Study Exempt from IND Requirements per 21 CFR 312.2(b)

Protocol Type / Version # / Version Date: Sponsor Amendment 5 / Version 6 / February 7, 2020

SCHEMA

N= 100
1 Cycle= 3wks

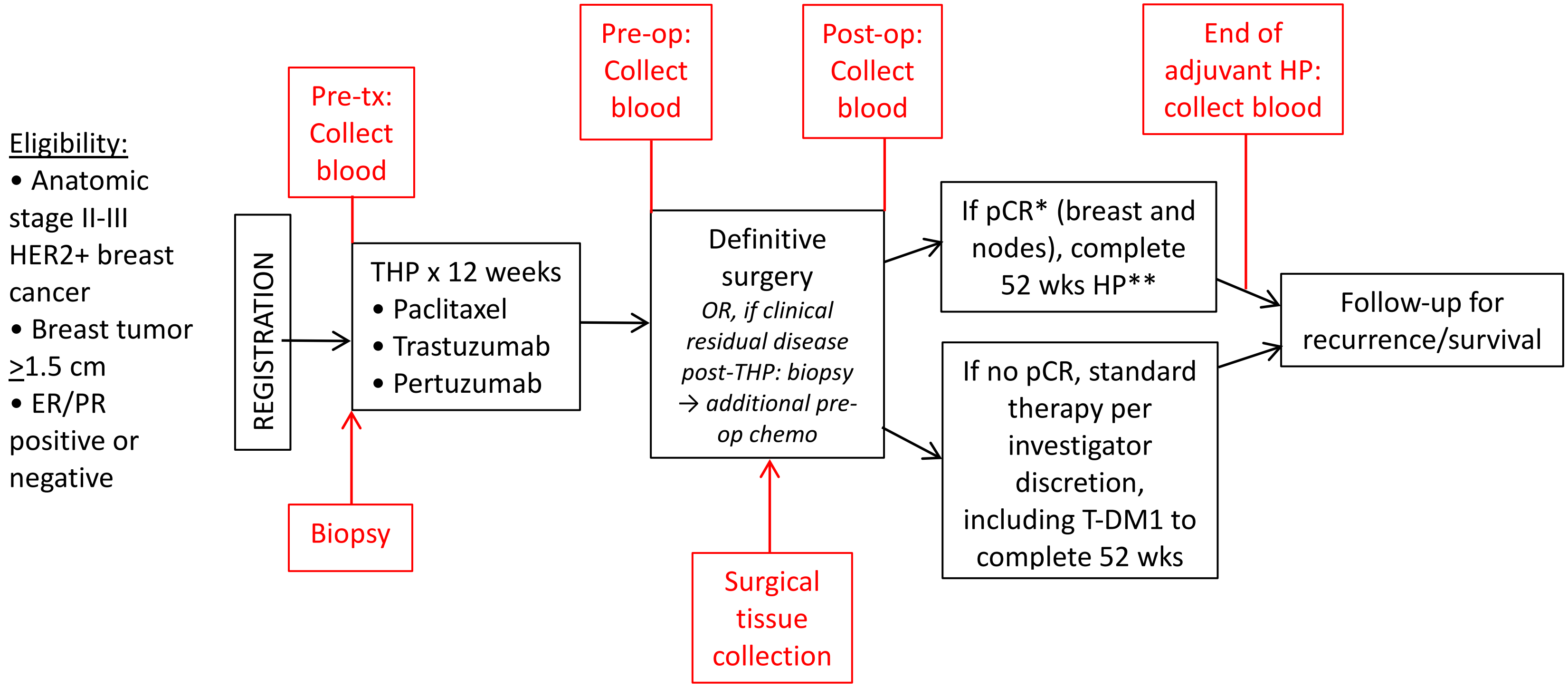

*pCR defined as: ypT0/is ypN0
**Concurrent endocrine tx allowed if HR+
Abbreviations: HP: trastuzumab/pertuzumab; HR: hormone receptor; THP: paclitaxel/trastuzumab/pertuzumab; pCR: pathologic complete response

TABLE OF CONTENTS

SCHEMA 3

1. OBJECTIVES 3
   - 1.1 Study Design 3
   - 1.2 Primary Objectives 3
   - 1.3 Secondary Objectives 3
   - 1.4 Correlative Objectives: 4

2. BACKGROUND 4
   - 2.1 Study Disease: HER2-positive breast cancer and neoadjuvant therapy 4
   - 2.2 Agents 6
   - 2.3 Rationale 9
   - 2.4 Correlative Studies Background 10

3. PARTICIPANT SELECTION 12
   - 3.1 Eligibility Criteria 12
   - 3.2 Exclusion Criteria 13
   - 3.3 Inclusion of Women and Minorities 14

4. REGISTRATION PROCEDURES 14
   - 4.1 General Guidelines for DF/HCC Institutions 14
   - 4.2 Registration Process for DF/HCC Institutions 14
   - 4.3 General Guidelines for Other Investigative Sites 15
   - 4.4 Registration Process for Other Investigative Sites 15

5. TREATMENT PLAN AND STUDY PROCEDURES 15
   - 5.1 Treatment Regimen 16
   - 5.2 Pre-Treatment Criteria 16
   - 5.3 Agent Administration 17
   - 5.4 Additional Pre-Surgical Therapy 18
   - 5.5 Medical History and Demographic Data 19
   - 5.6 Physical Examinations 19
   - 5.7 Research Blood and Tissue Sample Collection 19
   - 5.8 Tumor Staging 19
   - 5.9 Surgical Assessment 20
   - 5.10 Axillary Assessment and Axillary Surgery 20
   - 5.11 General Concomitant Medication and Supportive Care Guidelines 21
   - 5.12 Definitive Breast Surgery 22
   - 5.13 Post-operative Radiotherapy 22
   - 5.14 Post-operative Adjuvant Systemic Therapy 22
   - 5.15 Assessment of Adjuvant Systemic Therapy Decision-making 23
   - 5.16 Criteria for Taking a Participant Off Protocol Therapy 25
   - 5.17 Duration of Follow-Up 26

5.18 Criteria for Taking a Participant Off Study 27

6. DOSING DELAYS/DOSE MODIFICATIONS 27

7. ADVERSE EVENTS: LIST AND REPORTING REQUIREMENTS 28
7.1 Expected Toxicities 28
7.2 Adverse Event Characteristics 30
7.3 Expedited Adverse Event Reporting 30
7.4 Expedited Reporting to Hospital Risk Management 31
7.5 Routine Adverse Event Reporting 31

8. PHARMACEUTICAL INFORMATION 31
8.1 Trastuzumab 31
8.2 Paclitaxel 34
8.3 Pertuzumab 35

9. BIOMARKER, CORRELATIVE, AND SPECIAL STUDIES 37
9.1 Hypotheses 39
9.2 Overview of tissue collection protocols 39
9.3 Overview of blood collection protocols 41
9.4 Planned assays 42
9.5 Sites Performing Correlative Studies and/or Data Analysis 43
9.6 Tissue and blood banking 43

10. STUDY CALENDAR 44

11. MEASUREMENT OF EFFECT 46
11.1 Antitumor Effect – Solid Tumors 46
11.2 Radiographic assessment 46
11.3 Clinical assessments 46
11.4 Pathologic Response 46

12. DATA REPORTING / REGULATORY REQUIREMENTS 47
12.1 Data Reporting 47
12.2 Data Safety Monitoring 48
12.3 Multicenter Guidelines 48
12.4 Collaborative Research and Future Use of Data and Biospecimens 48

13. STATISTICAL CONSIDERATIONS 48
13.1 Study Design 48
13.2 Endpoints 49
13.3 Sample Size and Analysis of Primary Endpoint 50
13.4 Interim Monitoring Plan 50
13.5 Analysis of Secondary Endpoints 51

14. PUBLICATION PLAN 52

15. REFERENCES 53

16. APPENDIX A: PERFORMANCE STATUS CRITERIA 57

17. APPENDIX B: NEW YORK HEART ASSOCIATION (NYHA) CLASSIFICATIONS 58

18. APPENDIX C: DF/HCC MULTI-CENTER DATA AND SAFETY MONITORING PLAN 59

1. OBJECTIVES

1.1 Study Design

This is a single arm, open-label pilot trial enrolling patients with treatment-naïve stage II-III (according to AJCC cancer staging manual anatomic staging table, 8th edition) HER2+ breast cancer and a minimum primary tumor size of 1.5 cm. Patients with any hormone receptor status will be eligible. Following registration, all patients will receive neoadjuvant paclitaxel/trastuzumab/pertuzumab (THP), and pathologic response will be assessed at surgery per standard clinical practice. Patients with pathologic complete response (pCR; defined as ypT0/is N0) will complete one year of HP therapy in the adjuvant setting. Patients who do not achieve pCR will receive additional adjuvant therapy of the investigator's choice (four cycles of adjuvant adriamycin/cyclophosphamide (AC) chemotherapy is suggested but not mandated by the protocol), in addition to completing one year of HP in the adjuvant setting. In all patients with hormone receptor-positive (HR+) disease, any adjuvant hormonal therapy may be given at the investigator's discretion. All patients will be followed for recurrence and survival events. A tissue biopsy will be required at registration and serial blood collection will be performed.

1.2 Primary Objectives

To assess adherence to protocol-specified antibody doublet therapy (HP) in the adjuvant setting among patients with stage II-III HER2+ breast cancer who achieve pCR following neoadjuvant THP.

1.3 Secondary Objectives

- To assess pCR rate (defined as ypT0/is N0) in:
    a. All patients
    b. HR+ patients
    c. HR- patients
- To assess Residual Cancer Burden (RCB) scores[1] in:
    a. All patients
    b. HR+ patients
    c. HR- patients
- To assess reasons for off-protocol escalation (for patients with pCR) or de-escalation (for patients without pCR)
- To assess the percentage of patients completing one year of HP
- To measure event-free survival (EFS) and recurrence-free interval (RFI), and to compare EFS (or RFI) in the following subgroups:
    a. Patients with pCR versus patients without pCR
    b. Patients with RCB 0 or 1, versus patients with RCB 2 or 3
- To measure overall survival (OS) as an exploratory endpoint
- As an exploratory objective, to evaluate the correlation between post-THP imaging findings and pathology findings in the surgical specimen

1.4 Correlative Objectives:

- To assess how specific tumor-infiltrating lymphocyte (TIL) phenotype and T cell repertoire impact response to chemotherapy plus HER2-directed therapy.
- To assess how myeloid cells within the tumor microenvironment impact the response to standard THP administered neoadjuvantly to HER2+ breast cancer patients.
- To explore how the presence and functional activity of NK cells impacts response to anti-HER2 antibody therapy.

2. BACKGROUND

2.1 Study Disease: HER2-positive breast cancer and neoadjuvant therapy

HER2, also known as *neu* and *c-erbB-2*, is an oncogene encoding a tyrosine kinase growth factor receptor in the family of the epidermal growth factor receptor (EGFR), and is amplified in approximately 20% of all human breast cancers. It is an independent predictor of time to relapse and overall survival in multivariable models, and is a marker of poor prognosis.[2] The functional significance of HER2 amplification in breast cancer prompted development of trastuzumab, a monocloncal anti-HER2 antibody that was the first agent developed to target the HER2 pathway. Addition of trastuzumab to standard chemotherapy produces an overall survival benefit in HER2-amplified metastatic breast cancer.[3] Similarly, trastuzumab plus chemotherapy is superior to chemotherapy alone in multiple large, randomized trials of adjuvant therapy in HER2-positive disease, resulting in a striking reduction in the risk of relapse and death by 50% and 30%, respectively.[4-7] These results led to the approval of trastuzumab for use in the early stage disease setting.

In addition to large registrational studies, clinical trials conducted in the preoperative setting have contributed to the understanding of the activity of anti-HER2 therapies with the ability to provide an early read-out of drug efficacy. In the "first generation" of neoadjuvant anti-HER2 trials, patients were randomized to receive chemotherapy with or without trastuzumab.[8-10] These seminal studies confirmed the superiority of neoadjuvant trastuzumab in combination with chemotherapy, showing pCR rates at least two times higher in the trastuzumab containing arms (summarized in Table 1). In the "second generation" of neoadjuvant anti-HER2 trials, patients were randomized to receive one of two different anti-HER2 agents or to receive trastuzumab with or without another anti-HER2 agent in an effort to further improve the clinical benefit achieved with trastuzumab alone (Table 1) [11-13].

Table 1: Representative first and second generation neoadjuvant trials of HER2-targeted therapies

Studies and sample size	Study design	pCR * rates, p values
MD Anderson [8] N = 64	1) T→FEC<br>2) T + H→FEC + H	26.3<br>60 (P = NR)
NOAH [9] N = 235	1) AT→T→CMF<br>2) AT +H→T+ H→CMF +H	19<br>38 (P = 0.001)

NeoALTTO [14] N = 455	1) H → T + H → surgery → FEC → H until w 52<br>2) L → T → surgery → FEC → L until w 52<br>3) H + L → T + H + L → surgery → FEC → H + L until w 52	29.5<br>24.7 (2 vs. 1; P = 0.34)<br>51.3 (3 vs. 1; P = 0.0001)
NeoSphere [15] N = 417	1) H + D → surgery → FEC → H until w 52<br>2) H + PZ + D → surgery → FEC → H until w 52<br>3) H + PZ → surgery → H + D → FEC → H until w 52<br>4) PZ + D → surgery → FEC → H until w 52	29.0<br>45.8 (2 vs. 1; P = 0.0141)<br>16.8 (3 vs. 1; P = 0.019)<br>24.0 (4 vs. 2; P = 0.03)
NSABP B41 N = 529 [16]	1) AC → T + H → surgery → H until w 52<br>2) AC → T + L → surgery → H until w 52<br>3) AC → T + H+ L → surgery → H until w 52	52.5<br>53.2 (2 vs. 1; P = 0.99)<br>62.0 (3 vs. 1; P = 0.09)
CALGB 40601 N = 301 [17]	1) T + H → surgery → ddAC → H until w 52<br>2) T + L → surgery → ddAC → H until w 52<br>3) T + H + L → surgery → ddAC → H until w 52	46.0<br>37.0 (2 vs. 1; P =0.12)<br>56.0 (3 vs. 1; P = 0.12)
GeparQuinto [18] N = 620	1) EC + H → D + H → surgery<br>2) EC + L → D + L → surgery	31.3<br>21.7<br>P < 0.05

Abbreviations: A = doxorubicin; C = cyclophosphamide; D= docetaxel; E = epirubicin; F = fluorouracil; H = trastuzumab; L = lapatinib; NR = not reported; PZ = pertuzumab; pCR = pathologic complete response; T = paclitaxel; X = capecitabine. Comments: MD Anderson and NOAH studies have chemotherapy-only arms compared with chemotherapy plus trastuzumab (first generation studies). NeoALTTO, NeoSphere, GeparQuinto, NSABP-B41 and CALGB 40601 represent the second generation of neoadjuvant studies with anti-HER2 regimens in all study arms. pCR definitions: MD Anderson – No evidence of invasive cancer in breast or axilla; NOAH – Total pCR in breast and axillary nodes; NeoALTTO – non invasive cancer in the breast or only non invasive in situ cancer; NeoSphere – pathologic complete response in the breast GeparQuinto – no microscopic evidence of residual viable cells in any specimen.

While these second generation neoadjuvant studies hold considerable promise, it must be acknowledged that the pCR definitions used across these trials were not homogeneous (pCR definitions are described in Table 1), limiting cross-trial analysis.

Over the past years there have been significant improvements in our understanding of the biology of HER2-positive disease and several novel anti-HER2 drugs have been approved for the treatment of HER2-positive breast cancer.

Pertuzumab, another HER2-targeted humanized monoclonal antibody, represents the first of a novel class of drugs that have the ability to block the heterodimerization of HER2 with other members of the HER family (e.g., HER1, HER3). The resulting complementary and enhanced efficacy of HER2 blockade that is provided by the combination of trastuzumab plus pertuzumab has been demonstrated in both the preoperative setting (i.e. NeoSphere) and in the metastatic

setting where this combination of antibodies along with docetaxel leads to improved overall survival compared with trastuzumab and docetaxel [19].

2.2 Agents

2.2.1 Trastuzumab

Trastuzumab (Herceptin®) is a recombinant monoclonal antibody that binds specifically and with high affinity to the extracellular domain of HER2. Refer to the Full Prescribing Information for trastuzumab for complete safety information: http://www.accessdata.fda.gov/scripts/cder/drugsatfda/ in addition to information provided in Sections 5 and 8 of the protocol.

2.2.2 Paclitaxel

Paclitaxel is a chemotherapy agent in the taxane class. Refer to the Full Prescribing Information for paclitaxel for complete safety information: http://www.accessdata.fda.gov/scripts/cder/drugsatfda/ in addition to information provided in Sections 5 and 8 of the protocol.

2.2.3 Pertuzumab, and combination with trastuzumab/taxane

Pertuzumab in the early disease setting

Pertuzumab was combined with trastuzumab and/or chemotherapy in two neoadjuvant studies for the treatment of early-stage HER2-positive breast cancer. NEOSPHERE (Neoadjuvant Study of Pertuzumab and Herceptin in an Early Regimen Evaluation) evaluated the efficacy of 12 weeks of preoperative treatment with docetaxel plus pertuzumab or trastuzumab, or the combination of trastuzumab and pertuzumab in 417 women with early-stage HER2-positive breast cancer [20]. A fourth arm in this study assessed the activity of the trastuzumab/pertuzumab combination without accompanying chemotherapy. The primary endpoint of the study was pCR in the breast. Patients treated with the dual anti-HER2 combination plus docetaxel experienced a higher pCR rate (45.8%) compared to those who received only docetaxel/trastuzumab (29%) or only docetaxel/pertuzumab (24%). Interestingly, 17% of patients treated with trastuzumab plus pertuzumab for 12 weeks (without chemotherapy) also achieved pCR, suggesting that a subset of HER2 patients is highly sensitive to these anti-HER2 agents. In a subgroup analysis, lower pCR rates were observed across the four study arms among patients whose cancers were hormone receptor positive, compared to those patients with hormone receptor negative disease. Overall, neutropenia and febrile neutropenia were the most common serious adverse events, and occurred at similar frequencies in the chemotherapy-containing arms of the study. The mean maximum decrease in LVEF was 4% to 5% and was similar across the four treatment arms. A total of 6 patients experienced a decrease in LVEF of >10% from baseline, and to less than 50%; five of these 6 patients recovered

LVEF to more than 50% by cycle 4, and one patient with a previous history of cardiac disease had to discontinue treatment because of CHF.

In the TRYPHAENA (Trastuzumab plus Pertuzumab in Neoadjuvant HER2-positive Breast Cancer) study, a total of 225 HER2-positive breast cancer patients were randomized to receive trastuzumab and pertuzumab in combination with one of three chemotherapy regimens: concurrently with an anthracycline-taxane containing regimen (FEC [fluorouracil, epirubicin cyclophosphamide]-docetaxel); after FEC, but concurrently with docetaxel; or concurrently with the docetaxel/carboplatin combination [21]. The primary endpoint of the study was safety and tolerability. LVEF dropped by 6% in the concurrent anthracycline arm; by 4% in the sequential arm; and 3% in the carboplatin/docetaxel arm. Symptomatic LVEF dysfunction (grade 3 or higher) was recorded in 2.7% of the patients. pCR rates were 62%, 57% and 66% in the concurrent anthracycline, sequential and docetaxel/carboplatin treatment arms, respectively.

Lastly, the phase III APHINITY trial randomized patients with HER2+ stage I-III breast cancer receiving standard adjuvant chemotherapy plus trastuzumab to pertuzumab versus placebo. Overall, the addition of pertuzumab led to a small but statistically significant improvement in 3-year invasive disease-free survival (IDFS; 94.1% with pertuzumab versus 93.2% with placebo; HR 0.81, p=0.045). In subgroup analyses, a signal for risk reduction with pertuzumab persisted in node-positive and HR-negative patients, but was lost in node-negative and HR+ subgroups.[22] Of note, the magnitude of benefit from adding pertuzumab seemed to be greater in the patients with higher risk disease (i.e. node-positive or HR-negative, though interaction terms were not statistically significant).

Pertuzumab in advanced breast cancer

In a single arm phase II study, a total of 11 patients with HER2-positive breast cancer were treated with pertuzumab/trastuzumab [23]. The study had a target accrual of 37 patients but was stopped early when 6 of 11 (54%) patients experienced a decline in left ventricular ejection fraction (LVEF). Cardiotoxicity was more commonly observed among patients who had previously developed LVEF decline while on trastuzumab therapy, and in all 6 of the patients who had been previously received anthracycline-containing regimens. In addition, all patients had received trastuzumab treatment prior to entering the study, with a cumulative median duration of 82 weeks. The overall response rate (ORR) to the combination treatment was 18% and the median time to progression (TTP) was 6 weeks.

A subsequent single arm phase II study aimed at evaluating the same combination (trastuzumab and pertuzumab) enrolled a total of 66 HER2-positivepatients who had documented disease progression on a previous trastuzumab-based regimen [24]. Patients were excluded if they had experienced a symptomatic decrease in LVEF to less than 50% absolute value during prior trastuzumab therapy. Cardiac safety on the study was carefully monitored, and all echocardiograms were centrally re-evaluated. Three patients experienced a LVEF drop of ≥ 10 percentage points and to less than 50%, but no patient experienced symptoms related to cardiac toxicity. Two of the three patients that experienced LVEF drop remained on trastuzumab/pertuzumab treatment, and experienced subsequent recovery of cardiac function. The ORR was 24%, and the clinical

benefit rate (ORR plus stable disease ≥ 6 months) was 50%. The observed benefit was durable, with an overall median PFS of 5.5 months (range 0.9 to 17 months). The promising results of the trastuzumab/pertuzumab combination led to the recruitment of an additional cohort of patients who were treated with pertuzumab monotherapy, in order to investigate whether the trastuzumab was actually required [25]. A total of 29 patients whose disease progressed during prior trastuzumab therapy were treated with single agent pertuzumab until disease progression or unacceptable toxicity. A minimum of four weeks from the last dose of trastuzumab was required to reduce the confounding effect of the prior trastuzumab treatment on pertuzumab efficacy. The ORR and CBR for pertuzumab monotherapy were 3.4% and 10.3%, respectively. Seventeen of 29 patients progressing on pertuzumab monotherapy continued treatment with the addition of trastuzumab. The ORR and CBR for the combination in this population were 17.6% and 41.2%, respectively. These objective responses observed with the addition of trastuzumab to patients progressing on single agent pertuzumab appear to confirm the synergistic interaction of the two antibodies that was demonstrated in preclinical models.

In addition to the cardiac safety issues, the most frequent adverse events observed in the phase II setting with pertuzumab/trastuzumab, or with pertuzumab monotherapy, were diarrhea, nausea, fatigue, and rash, with most being of mild to moderate intensity (grade 1 or grade 2).

CLEOPATRA (Clinical Evaluation Of Pertuzumab And Trastuzumab) is a randomized double-blind, placebo controlled phase III study that was designed to evaluate the effectiveness of the trastuzumab/pertuzumab combination [26]. A total of 808 patients with advanced HER2-positive breast cancer were randomized to receive trastuzumab and docetaxel, with either pertuzumab or placebo. Eligible patients could not have received prior chemotherapy or targeted therapy in the metastatic setting and only 10% of enrolled patients had received trastuzumab in the early-stage setting. The study primary endpoint was independently assessed PFS; secondary endpoints were overall survival OS, ORR, and safety. The addition of pertuzumab to trastuzumab and docetaxel significantly prolonged PFS, from a median of 12.4 to 18.5 months (hazard ratio [HR] 0.62; 95% CI 0.51-0.75; $P < 0.001$). LVEF dysfunction was more common in the control arm (trastuzumab/docetaxel) than in the pertuzumab-containing arm (8.3% versus 4.4%, respectively). The following adverse events were more common in the pertuzumab than placebo-containing arm: diarrhea (66.8% v 46.3%), mucosal inflammation (33.7% v 24.2%), febrile neutropenia (13.8% v 7.6%), and dry skin (10.6% v 4.3%). In a subsequent analysis with 30 months of median follow-up, the addition of pertuzumab to the trastuzumab/docetaxel combination conferred a 34% reduction in the risk of mortality (HR = 0.66; p =0.0008) [27]. In a subgroup analysis by age, treatment benefit was similar in patients ≥ 65 years compared to that in younger patients [28]. Quality of life assessment showed no detrimental effect with the addition of pertuzumab [29]. In the biomarker analysis of the CLEOPATRA study, PIK3CA gene mutations were identified in 32% of patients (176/557 patients), and patients with PIK3CA gene mutation had worse outcome when compared to those with wild type PIK3CA gene status. However, PIK3CA mutation(s) were not associated with resistance to pertuzumab and the magnitude of benefit of pertuzumab was independent of PIK3CA mutational status [30]. The positive results from CLEOPATRA led to approval by the U.S. Food and Drug Administration for

use of pertuzumab, in combination with trastuzumab and docetaxel, for the treatment of patients with HER2+ advanced breast cancer who have not received prior anti-HER2 therapy or chemotherapy for metastatic disease.

Refer to the Full Prescribing Information for Pertuzumab for complete safety information: http://www.accessdata.fda.gov/scripts/cder/drugsatfda/ in addition to information provided in Sections 5 and 8 of the protocol.

2.3 Rationale

2.3.1 De-escalation of adjuvant therapy following pathologic complete response in HER2+ breast cancer

In this protocol, we plan to examine the feasibility of de-escalating therapy from a multi-agent to a single-agent chemotherapy backbone plus HP in patients with stage II-III HER2+ breast cancer, based on pCR as a prognostic biomarker. At present, standard-of-care neo/adjuvant therapy for stage II-III HER2+ breast cancer involves either adriamycin/cyclophosphamide followed by taxane plus trastuzumab (AC-TH) or taxane/carboplatin/trastuzumab concurrently (TCH), with consideration of pertuzumab in all patients.[31] These multi-agent chemotherapy regimens are associated with multiple short-and long-term toxicities including alopecia, fatigue, neuropathy, cytopenias, cardiomyopathy, and secondary leukemia,[6] with impacts ranging from common but temporary reduction in quality of life to rare and life-threatening conditions.

Though HER2+ breast cancer was historically associated with a worse prognosis than other types of breast cancer,[2] in recent years there has been explosive development and FDA approval of numerous effective HER2-targeted therapies. In 2017 the large majority of patients with non-metastatic HER2+ disease is cured with modern regimens incorporating chemotherapy plus HER2-directed therapy. In the recently published APHINITY trial of adjuvant multi-agent chemotherapy plus trastuzumab with or without pertuzumab, 3-year invasive-disease-free survival was 93-94% for all patients, and 90-92% in patients with node-positive disease.[22] Clearly, ongoing efforts must strive to understand the patients for whom HER2+ breast cancer recurs despite these highly active regimens, and additional treatment options are needed for those patients. At the same time, the excellent long-term outcomes of most patients treated with modern regimens for non-metastatic HER2+ disease mean that we must begin to thoughtfully identify patients who can be cured with less toxic, less intensive therapy.

We plan to investigate pCR-based de-escalation: selecting patients who are likely to have excellent long-term outcomes with less treatment, on the basis of pCR following a standard-of-care neoadjuvant regimen. De-escalating therapy on the basis of pCR makes sense for a number of reasons: Pathologic complete response at surgery provides real-time, concrete evidence of response to a given therapy for a given patient; and it is widely recognized as a positive prognostic biomarker in breast cancer.[32-34] If pCR-based de-escalation is found to be a feasible strategy that maintains excellent long-term outcomes for patients with HER2+ breast cancer, it has the potential to spare a select group of breast cancer patients the short- and long-term toxicities of multi-agent chemotherapy,

without compromising their long-term survival.

Given the novelty of a de-escalated treatment regimen in stage II-III HER2+ breast cancer, it is important to assess the feasibility and streamline the execution of this approach before proceeding to a large outcome-based trial. This feasibility-focused trial is a precursor to a significantly larger trial to be run through the Alliance for Clinical Trials in Oncology, investigating long-term patient outcomes with the same treatment approach (anticipated to open in approximately 3 years).

2.3.2 Choice of regimen: neoadjuvant paclitaxel/trastuzumab/pertuzumab (THP)

On this prospective protocol, de-escalated therapy patients (i.e. those that achieve pCR) will receive only single-agent chemotherapy with paclitaxel (T) in addition to trastuzumab/pertuzumab (HP). THP is an optimal de-escalated regimen for a number of reasons. First, regimens incorporating doublet HER2-directed monoclonal antibodies (trastuzumab plus pertuzumab) show high levels of activity in both metastatic and non-metastatic HER2+ breast cancer, and improved outcomes compared to H alone.[22,35,36] Indeed, in the phase II NeoSphere trial, 16.8% of patients treated with HP alone in the neoadjuvant setting had pathologic complete response (pCR) at surgery.[35] Second, in stage I HER2+ breast cancer, long-term outcomes are very favorable with paclitaxel plus trastuzumab, and this single-agent chemotherapy backbone is now standard-of-care in that setting.[37]

Although the taxane chemotherapy backbone used with HP in the NeoSphere and TRYPHAENA studies (both evaluating neoadjuvant taxane plus HP) was docetaxel, national guidelines support the use of paclitaxel over docetaxel.[31] Safety data from a prospective clinical trial of metastatic HER2+ breast cancer patients treated with paclitaxel plus HP suggest that it is associated with less toxicity than a docetaxel-based regimen.[38]

2.4 Correlative Studies Background

Overall, the rationale for the proposed correlative research is the need to determine the impact of trastuzumab and pertuzumab, two HER2-targeted monoclonal antibodies, on the tumor's immune microenvironment. Our overarching hypothesis is that the immune microenvironment and patients' peripheral immune system will impact response to standard therapy including paclitaxel, trastuzumab and pertuzumab (THP) and that characterization of this interplay will inform the design of rational therapeutic strategies targeting the microenvironment to enhance response to standard therapy. The proposed correlative research is expected to be impactful because it will fully characterize immunologic aspects of the tumor microenvironment in HER2+ breast cancer before and after receipt of standard therapy with paclitaxel, trastuzumab and pertuzumab.

2.4.1 Examining the immune microenvironment pre- and post-HER2-directed therapy in HER2-positive breast cancer

A recent pooled analysis evaluated the predictive value of tumor-infiltrating lymphocytes

(TIL) in breast cancer patients receiving neoadjuvant therapy.[39] Stromal TIL in pretreatment core biopsies were assessed for the number of stromal TILs by standardized methodology according to the guidelines of the International TIL working group.[40] TILs were analyzed as a continuous variable and patients were assigned to one of 3 groups: low (0-10% TIL), intermediate (11-59%), and high (≥60%). In patients with HER2+ breast cancer, a pCR was experienced in 194 (32%) of 605 patients with low TILs, 198 (39%) of 512 patients with intermediate TILs, and 127 (48%) of 262 with high TILs ($p<0.0001$).[39] These data suggest that the presence of TIL does not guarantee a pCR and conversely, the absence of TIL does not preclude a pCR. A number of groups have proposed employing strategies to enhance TIL infiltrate in order to increase rates of pCR. In order for this to be considered as a therapeutic strategy, it is critical to more fully characterize the TIL phenotype which includes CD4 and $CD8^+$ T cells, regulatory T cells, B cells and natural killer (NK) cells.

There is a growing body of literature implicating myeloid cells in the pathogenesis of breast cancer. As an example, one common myeloid cell found within the microenvironment of triple negative breast cancer are macrophages. Tumor associated macrophages (TAM) are a heterogeneous population of macrophages. Although this polarization is idealized, it has generally been thought that there are 2 phenotypes – M1 which are potent effector cells that kill tumor cells and recruit cytotoxic T cells, and M2 which secrete anti-inflammatory cytokines and attract regulatory T cells and Th2 T cell subsets that do not have cytotoxic function. TAMs are generally more M2-like and show pro-tumor functions by promoting tumor survival, proliferation, angiogenesis, and dissemination. While macrophages have been shown to be one of two major populations of infiltrating immune cells (along with T cells) in triple negative breast cancer, less is known regarding the presence of macrophages in HER2-positive breast cancer. A recent study in a mouse model of HER2+ breast cancer (MMTV-HER2) demonstrated macrophage infiltration as an early event that promoted early dissemination affecting long-term metastasis development. TAMs are recruited to tumors through tumor-derived cytokines including CSF-1 and CCL2. Agents targeting CSF1 (anti-CSF-1 antibodies and CSF-1R inhibitors) are being evaluated in clinical trials.

2.4.2 Examining NK cell activity and response to HER2-directed therapy in HER2-positive breast cancer

Multiple mechanisms of action have been proposed for trastuzumab including activation of both innate and adaptive cellular immunity. [41] In vivo models have confirmed antibody-dependent cell-mediated cytotoxicity (ADCC) as one mechanism of action with efficacy correlating with the presence of NK cells. [42,43] ADCC involves the Fc-gamma receptor (FcγR) on NK cells or macrophages cross-linking cell-bound antibodies (i.e. trastuzumab) leading to release of granzyme and perforin into the synapse, promoting apoptosis. Small pilot studies evaluating patients with HER2+ operable breast cancer have confirmed ADCC as a likely mechanism of trastuzumab therapy and have suggested a correlation between the presence of Fc receptors (CD16+) on the surface of NK cells and macrophages with efficacy. To date, limited work has been done to determine the

role of peripheral NK cell activity (i.e. function vs. phenotype) or the presence of NK cells in the tumor microenvironment on the response to trastuzumab therapy. And while ADCC has been demonstrated as a mechanism of action of pertuzumab in in vivo models[44], less is known regarding the impact of NK cell presence and activity on response to pertuzumab (or the combination of trastuzumab and pertuzumab) in patients.

3. PARTICIPANT SELECTION

3.1 Eligibility Criteria

3.1.1 Patients must have Stage II or III (according to AJCC cancer staging manual anatomic staging table, 8th edition) histologically confirmed invasive carcinoma of the breast. A minimum tumor size of 1.5 cm determined by physical exam or imaging (whichever is larger) is required. Patients with inflammatory breast carcinoma (T4d) are NOT eligible.

3.1.2 Tumors must be HER-2 positive by 2018 criteria, as assessed by standard local institutional protocol (central testing is not required):

- IHC 3+

-AND/OR-

- FISH positive based on the following criteria:
  - Dual-probe HER2/CEP17 ratio $\geq$2.0 with an average HER2 copy number $\geq$ 6.0 signals/cell

-AND/OR-

- Based on 2018 American Society of Clinical Oncology/College of American Pathologists guidelines for HER2 testing

3.1.3 ER/PR determination is required. ER- and PR-assays should be performed by immunohistochemical methods according to the local institution standard protocol.

3.1.4 Bilateral breast cancers are allowed as long as both cancers are HER2-positive (as defined in 3.1.2), or the contralateral cancer is a $\leq$1 cm, ER+, and HER2- tumor.

3.1.5 Patients with multifocal or multicentric disease are eligible if the treating physician has determined the patient should be treated as HER2-positive.

3.1.6 Breast imaging should include dedicated ultrasound of the ipsilateral axilla. For subjects with a clinically positive axilla based on exam or imaging, a fine needle aspiration or core biopsy procedure will be performed to determine the presence of metastatic disease in the lymph nodes (though lymph node sampling procedure need not be resulted prior to

patient's registration on trial, as long as all other eligibility are met).

3.1.7 Men and women (with any menopausal status) ≥ 18 years of age are eligible.

3.1.8 ECOG PS 0 or 1. (See Appendix A).

3.1.9 Required laboratory values:

- ANC ≥ 1000/mm^3
- Hemoglobin ≥ 9 g/dl
- Platelets ≥ 100,000/mm^3
- Serum creatinine < 1.5 x ULN (institutional) OR calculated GFR ≥60mL/min
- Total bilirubin ≤ 1.5 x ULN (institutional). For patients with Gilbert Syndrome, the direct bilirubin should be within the institutional normal range OR total bilirubin ≤ 2.0 mg/dL.
- AST and ALT ≤ 2.5x ULN (institutional)

3.1.10 Left ventricular ejection fraction (LVEF) ≥ 50%.

3.1.11 Premenopausal women must have a negative serum pregnancy test within 14 days of registration, including women who have had a tubal ligation and for women less than 12 months after the onset of menopause.

3.1.12 Women of childbearing potential and men with partners of childbearing potential must be willing to use effective contraception by the patient and/or partner and continue its use for the duration of the study treatment and for 7 months after the last dose of study treatment.

3.1.13 Patients with a history of ipsilateral DCIS are eligible.

3.1.14 Patients undergoing breast conservation therapy (i.e. lumpectomy) must not have any contraindications to radiation therapy.

3.1.15 Non-English-speaking patients are eligible, but will be exempt from patient-completed questionnaires.

3.1.16 Willing and able to sign informed consent.

3.1.17 Willing to provide tissue for research purposes.

3.2 Exclusion Criteria

3.2.1 Pregnant or nursing women due to the teratogenic potential of the study drugs.

3.2.2 Active, unresolved infection.

3.2.3 Receipt of intravenous antibiotics for infection within 7 days prior to registration.

3.2.4 Uncontrolled hypertension (systolic >180 mm Hg and/or diastolic >100 mm Hg) or clinically significant (i.e. active) cardiovascular disease: cerebrovascular accident/stroke or myocardial infarction within 6 months prior to first study medication, unstable angina, congestive heart failure (CHF) of New York Heart Association (NYHA) Class II or higher (see Appendix B), or serious cardiac arrhythmia requiring medication.

3.2.5 Significant symptoms (Grade ≥ 2) from peripheral neuropathy.

3.2.6 Other concurrent serious diseases that may interfere with planned treatment, including severe pulmonary conditions/illness, uncontrolled infections, uncontrolled diabetes.

3.2.7 Any prior treatment for the current breast cancer, including chemotherapy, hormonal therapy, radiation, or experimental therapy.

3.3 Inclusion of Women and Minorities

Both men and women of all races and ethnic groups are eligible for this trial. Every effort will be made to include participants from minority populations. Because breast cancer predominantly affects females, it is anticipated that male enrollment will be < 5% of the overall study population.

4. REGISTRATION PROCEDURES

4.1 General Guidelines for DF/HCC Institutions

Institutions will register eligible participants in the Clinical Trials Management System (CTMS) OnCore. Registrations must occur prior to the initiation of protocol therapy. Any participant not registered to the protocol before protocol therapy begins will be considered ineligible and registration will be denied.

An investigator will confirm eligibility criteria and a member of the study team will complete the protocol-specific eligibility checklist.

Following registration, participants may begin protocol therapy. Issues that would cause treatment delays should be discussed with the Overall Principal Investigator (PI). If a participant does not receive protocol therapy following registration, the participant's registration on the study must be canceled. Registration cancellations must be made in OnCore as soon as possible.

4.2 Registration Process for DF/HCC Institutions

DF/HCC Standard Operating Procedure for Human Subject Research Titled *Subject Protocol Registration* (SOP #: REGIST-101) must be followed.

4.3 General Guidelines for Other Investigative Sites

Eligible participants will be entered on study centrally at the Dana-Farber Cancer Institute by the Project Manager. All sites should email or call the Project Manager to verify slot availabilities. The required forms in Section 4.4 should be emailed or faxed to the Project Manager.

Following registration, participants should begin protocol therapy within 7 days. Issues that would cause treatment delays should be discussed with the Overall PI. If a participant does not receive protocol therapy following registration, the participant's registration on the study must be canceled. The Project Manager should be notified of cancellations as soon as possible.

4.4 Registration Process for Other Investigative Sites

To register a participant, the following documents should be completed by the participating site and emailed to the Project Manager at CTOPM@dfci.harvard.edu or faxed to 617-632-5152:

- Pathology report and documentation of ER, PR, and HER2 status
- Clinic visit note documenting history and physical exam, ECOG PS, and vital signs
- Copy of required laboratory tests including: Hematology, Chemistry, and pregnancy test (for women of childbearing potential)
- ECHO or MUGA Report
- Mammogram, Ultrasound, or MRI Report
- Signed participant consent form
- HIPAA authorization form (if separate from the main consent form)
- Completed DF/HCC Eligibility Checklist

To complete the registration process, the Project Manager will

- Follow the DF/HCC Standard Operating Procedure for Human Subject Research Titled *Subject Protocol Registration* (SOP #: REGIST-101) and register the participant on the protocol
- email the research nurse or data manager at the participating site with the participant study number, and registration confirmation

<u>NOTE</u>: **Registrations can only be conducted during the business hours of 8:00 AM and 5:00 PM Eastern Time Monday through Friday.** Same day treatment registrations will only be accepted with prior notice and discussion with the DF/HCC Project Manager.

5. TREATMENT PLAN AND STUDY PROCEDURES

Data will be collected and maintained on study specific case report forms (CRFs). See Section 10 Study Calendar for additional details. Reported adverse events and potential risks are described in Section 7. Appropriate dose modifications are described in Section 6. No investigational or commercial agents of therapies other than those described below may be administered with the intent to treat the participant's malignancy.

5.1 Treatment Regimen

In this study, one cycle is defined as 3 weeks. Study treatment will be administered in 21-day (3-week) cycles if no additional time is required for reversal of toxicity. Ideally treatment will be administered within a +/- 3 day window; however, if a patient has a conflict with the timing of a protocol-mandated procedure, the procedure should be performed on the nearest possible following or preceding date. Reasonable effort should be made to conduct study visits on the day scheduled (+/- 3 days).

Treatment will typically be administered on an outpatient basis. Sites may use their institutional standards (e.g., the Dubois formula) for calculating the doses of paclitaxel and trastuzumab.

**Regimen Description****					
**Agent***	**Premedications; Precautions**	**Dose**	**Route**	**Schedule**	**Cycle Length**
Paclitaxel	Premedications should be administered according to institutional guidelines	80 mg/m^2	IV per local standard operating procedures	Days 1, 8, and 15 of each 21-day cycle	21 days (3 weeks)
Trastuzumab	No routine premedications	8 mg/kg for initial dose; 6 mg/kg for subsequent doses	IV per local standard operating procedures	Day 1 of each 21-day cycle	
Pertuzumab	No routine premedications	840 mg for initial dose; 420 mg for subsequent doses	IV per local standard operating procedures	Day 1 of each 21-day cycle	
*Order of infusion for the three medications may be determined by local standard operating procedures.<br>**Further administration details, including observation periods follow in Section 5.3.					

5.2 Pre-Treatment Criteria

The following should be considered as a guideline for proceeding with treatment at cycle 1 day 1 and on subsequent day 1's of each cycle. Some modification is permissible based on investigator

judgment and local institutional standards.

Guideline criteria to treat at cycle 1 day 1, and subsequent cycle day 1 treatment days:

- Absolute neutrophil count $\geq$1000/mm^3
- Platelets $\geq$100,000/mm^3
- Total bilirubin ≤ 1.5 x ULN (institutional).
- AST and ALT $\leq$2.5 x ULN (institutional)
- Serum creatinine $\leq$ 1.5 x ULN (institutional) OR calculated GFR ≥60mL/min

The management of lab abnormalities/decisions to treat or hold drug throughout each cycle, including on cycle days 8 and 15, is at the discretion of the treating investigator.

5.3 Agent Administration

5.3.1 Paclitaxel

Paclitaxel will be administered in clinic on Days 1, 8, and 15 of each 21-day cycle (with recommended window of +/- 3 days, see Section 5.1) per local standard operating procedures. The dose for the initial treatment with paclitaxel is 80mg/m^2 IV per week. Treatment will typically be administered on an outpatient basis. Dose modifications and toxicity management for paclitaxel are described in Section 6.

Preparation and administration of paclitaxel, including premedications (e.g. dexamethasone), should follow institutional guidelines. Anti-emetics typically should not be administered prophylactically before initial treatment with study drug, though this may be modified based on the investigator’s discretion for a given patient.

Protocol therapy will consist of 12 doses (4 cycles) of paclitaxel delivered in the neoadjuvant setting. Following surgery, it is not expected that patients will receive additional paclitaxel in the adjuvant setting.

5.3.2 Trastuzumab

Trastuzumab will be administered on Day 1 of each 21-day cycle (with recommended window of +/- 3 days, see Section 5.1) per local standard operating procedures. The loading dose for the initial treatment with trastuzumab is 8 mg/kg IV. All subsequent doses are 6 mg/kg IV every 3 weeks. If there are delays between doses, loading doses should be administered per institutional guidelines.

The following are guidelines for trastuzumab infusion and may be modified per local institutional standards. Participants who develop adverse reactions to the infusion should receive subsequent infusions per local institutional standards.

Trastuzumab should not be mixed or diluted with other drugs. Trastuzumab infusions

should not be administered or mixed with dextrose solutions. No incompatibilities between trastuzumab and polyvinylchloride or polyethylene bags have been observed. No hydration is required.

Premedication for nausea and infusion reactions are not commonly required but may be given at the investigator's discretion.

Protocol therapy will consist of 4 doses of trastuzumab (4 cycles) delivered in the neoadjuvant setting. As per Section 5.4, in cases of surgical delay up to 2 additional doses of trastuzumab may be administered preoperatively. Following surgery, all patients may receive at least 13 doses of additional trastuzumab, for a total of 17 doses.

5.3.3 Pertuzumab

Pertuzumab will be administered on Day 1 of each 21-day cycle (with recommended window of +/- 3 days, see Section 5.1) per local standard operating procedures. The initial loading dose of Pertuzumab is 840 mg. The pertuzumab dose in subsequent cycles is 420 mg. Timing of infusion and observation may be modified as necessitated by standard institutional practice. For delayed or missed doses of Pertuzumab, if the time between 2 sequential infusions is less than 6 weeks, the 420 mg IV dose of Pertuzumab may be administered. If the time between 2 sequential infusions is 6 weeks or more, the initial dose of 840 mg Pertuzumab may be re-administered as a 60-minute IV infusion followed every 3 weeks thereafter by a dose of 420 mg IV administered over 30 minutes.

Premedication for nausea and infusion reactions are not commonly required but may be given at the investigator's discretion.

Protocol therapy will consist of 4 doses of pertuzumab (4 cycles) delivered in the neoadjuvant setting. As per Section 5.4, in cases of surgical delay up to 2 additional doses of pertuzumab may be administered preoperatively. Following surgery, all patients may receive at least 13 doses of additional pertuzumab, for a total of 17 doses.

5.4 Additional Pre-Surgical Therapy

Participants may receive up to two additional trastuzumab and pertuzumab (HP) doses before surgery (maintaining the standard schedule of 3 weeks between HP doses), if surgical date is more than two weeks from the final preoperative paclitaxel dose. Receipt of additional preoperative HP is not required and is up to investigator discretion.

In participants with evidence of disease progression on neoadjuvant THP, additional pre-surgical therapy is allowed (and constitutes a possible reason for taking a patient off of the treatment protocol, see Section 5.16). The selected treatment regimen will be at the discretion of the treating investigator.

In participants with obvious residual disease as determined by physical examination or imaging, if performed, following completion of neoadjuvant THP, additional systemic therapy may be given in the neoadjuvant setting at the investigator's discretion (and, if given, should be recorded in the CRF); four cycles of adriamycin and cyclophosphamide is the recommended regimen in this case. In this case, a biopsy of the presumed residual disease is strongly recommended for clinical purposes (to confirm presence of malignant cells) and biopsy should, at the same time, be obtained for research purposes (see Section 9). This biopsy performed at the time of treatment change, prior to initiation of new anti-cancer therapy, is strongly recommended, but not required. Participants who receive additional pre-surgery therapy will continue to be followed per protocol Sections 5.9 through 5.18.

5.5 Medical History and Demographic Data

Medical history includes past or current clinically significant conditions, surgeries, breast cancer surgery and diagnosis, reproductive status, and all medications (e.g., prescription drugs, over the counter drugs, herbal or homeopathic remedies, nutritional supplements) used by the patient within 7 days prior to registration. Demographic data will include age, sex, and self-reported race/ethnicity.

5.6 Physical Examinations

At the initial study visit, physical examination should include examination of breast and local-regional lymphatics. Clinical T and N staging according to AJCC cancer staging manual anatomic staging table 8th edition should be documented. At subsequent visits with medical or surgical providers (or more frequently, if clinically indicated), breast examination and evaluation of local-regional lymphatics should be performed, with breast tumor measurements performed and documented in CRFs. Additional physical examinations should be focused on organ systems related to adverse events. It is anticipated and recommended that physical examinations will occur at least approximately once every 3 weeks (i.e. at day 1 of each cycle), but slight deviations from this schedule are allowable per investigator discretion. Weight is to be measured at Pre-Study (≤ 28 days of registration) and on Day 1 of the specified cycles. Vital signs will include measurements of pulse rate, systolic, and diastolic blood pressures, and temperature.

5.7 Research Blood and Tissue Sample Collection

Procedures and timepoints for collecting and processing blood and tissue specimens for research purposes are located in Sections 9.2 and 9.3 and study calendar (Section 10).

5.8 Tumor Staging

5.8.1 Breast mammogram, ultrasound, MRI

All subjects are required to have a mammogram and diagnostic breast ultrasound (with or without breast MRI*) performed at screening (with at least one of these imaging studies falling within 42 days of registration) and closely preceding or following the last dose of neoadjuvant therapy (from up to one week before to a maximum of up to 4 weeks after

the last dose of neoadjuvant therapy), to facilitate surgical planning. Baseline breast imaging should include imaging of the ipsilateral axilla. The same imaging modality must be used at screening and prior to surgery to assess tumor response (with the exception that breast MRI, if performed at screening, is strongly recommended but not required to be repeated prior to surgery).

* Breast MRI is strongly recommended in patients in whom breast conserving therapy is under consideration, although not required if practical or financial considerations preclude MRI, as long as the target lesion can be adequately measured by mammogram and/or ultrasound.

5.8.2 CT scans

Subjects with Stage III disease according to AJCC Staging Manual anatomic staging table, edition 8, will have CT scans of chest, abdomen and pelvis with or without a bone scan (whole body PET scan as an alternative is allowed but not preferred) performed during screening to rule out metastatic disease.

5.9 Surgical Assessment

All subjects will be seen and examined by the treating surgeon during screening and at the Pre-Operative Visit. Each visit will include a clinical breast and lymph node examination and review of the imaging studies (mammogram, MRI, and any other radiographic method) of the breast.After examining the subject and reviewing the pertinent radiographic studies at the Screening and pre-operative visits, the surgeon will determine whether the subject is eligible for breast conserving surgery. If the subject is not a breast conservation candidate, the reason(s) will be documented in the CRF (multicentric tumor, tumor location, extensive calcifications on imaging, other).

5.10 Axillary Assessment and Axillary Surgery

An axillary assessment will be performed as part of screening. Ipsilateral axillary lymph nodes will be assessed as clinically normal or clinically suspicious by physical examination. In all patients, ipsilateral axillary lymph nodes will also be assessed as normal or suspicious independently by dedicated axillary ultrasound. Axillary ultrasound and/or biopsy do not need to be repeated if performed prior to the screening period. Subjects with suspicious nodes documented by physical exam OR by ultrasound will have a biopsy of the nodes (fine needle aspirate or core needle biopsy).

For patients with clinically node negative disease, sentinel lymph node (SLN) biopsy will be performed after neoadjuvant systemic therapy. Mapping technique (i.e. single versus dual tracer) will be at the discretion of the attending surgeon. For patients with clinically node positive disease at presentation who become clinically node negative by physical examination following completion of their neoadjuvant systemic therapy, SLN biopsy will be performed after preoperative therapy. Dual tracer with both radioisotope and blue dye will be employed. For patients in whom mapping fails or in whom fewer than 3 SLN are identified, an axillary lymph node dissection (ALND) will be performed at the time of the initial operation. Identified SLN(s)

will be sent for intraoperative frozen section evaluation. If the SLN(s) are positive on this intraoperative frozen section evaluation or on final pathologic evaluation by either hematoxylin & eosin (H&E) staining or by immunohistochemistry (IHC) staining for cytokeratin, then a completion ALND will be performed. Positivity on final pathologic evaluation will include the finding of any disease to include micrometastasis or isolated tumor cells regardless of how detected (i.e H&E or IHC). For patients with clinically node positive disease at presentation who remain clinically node positive by physical examination following completion of their neoadjuvant systemic therapy, an ALND will be performed. Of note, this axillary surgical management approach is required only at the coordinating center (Dana-Farber), though is strongly recommended at other study sites.

5.11 General Concomitant Medication and Supportive Care Guidelines

Concomitant therapy includes any medication (e.g., prescription drugs, over-the-counter drugs, herbal/homeopathic remedies, nutritional supplements) used by a patient from 7 days prior to registration to the end of treatment visit. All concomitant medications should be reported to the investigator and recorded in the medical record.

The following treatments are permitted throughout the duration of the study treatment phase and during follow-up:

- Standard therapies for pre-existing medical conditions unless listed as prohibited therapy in section below. Any medication intended solely for supportive care (e.g., analgesics, anti-diarrheal, anti-depressants) may be used at the investigator's discretion.
- Hematopoietic growth factors (e.g., G-CSF, granulocyte macrophage colony stimulating factor) may be used at investigator's discretion for the primary prophylaxis and/or management of treatment-emergent neutropenia and/or for secondary prophylaxis as per NCCN/European Society for Medical Oncology guidelines [45,46] or local standard practice.
- Bisphosphonate or denosumab therapy to be used in accordance with the approved labeled indication and/or nationally recognized treatment guidelines.
- Hormonal contraception is permitted if deemed medically necessary per investigator discretion, though cessation of hormonal contraception is strongly encouraged in patients with hormone receptor-positive tumors.
- Estrogen replacement therapy is permitted in patients with hormone receptor-negative tumors. Estrogen replacement therapy is NOT permitted in patients with hormone receptor-positive tumors.

Explicitly prohibited therapies prior to the end of treatment visit include:

- Anti-cancer therapies other than those administered in this study, including cytotoxic chemotherapy, radiotherapy (except for adjuvant radiotherapy for breast cancer after completion of chemotherapy), immunotherapy, biological, hormonal or targeted (e.g., lapatinib, neratinib) anti-cancer therapy
- Estrogen replacement therapy is not permitted in patients with hormone receptor-positive tumors.
- Any investigational agent, except those used for this study

5.12 Definitive Breast Surgery

Definitive breast surgery (lumpectomy and/or mastectomy) can be performed as soon as 14 days but no later than 42 days following administration of the last dose of protocol therapy (unless an additional preoperative systemic therapy regimen is administered, as described in Section 5.4). If contralateral mastectomy is performed concurrently, the pathology report from the contralateral breast must be reported if invasive disease is identified.

For guidance regarding axillary surgery, see Section 5.10 above.

5.13 Post-operative Radiotherapy

Decisions regarding post-surgical radiotherapy will be made by the treating team.

5.14 Post-operative Adjuvant Systemic Therapy

For the protocol to succeed, accurate recording of adjuvant systemic therapy planned and received is essential.

Note that assessment of pCR must occur after the final breast cancer surgery is performed (i.e. if patient returns to the operating room for ALND, pCR status cannot be ascertained until ALND pathology is reviewed).

5.14.1 Patients who achieve pCR at the time of surgery (defined as ypT0/is ypN0): These patients should go on to receive antibody-only therapy with trastuzumab and pertuzumab (HP) for at least 13 doses in the post-operative setting, for a total of at least 17 doses of HP overall (4 doses pre-operatively and 13 doses post-operatively). For patients with HR+ disease, any regimen of adjuvant hormonal therapy may be given at the investigator's discretion. Receipt of adjuvant bone modifying agents will not be considered in the assessment of escalated or de-escalated adjuvant therapy.

5.14.2 Patients who do not achieve pCR at the time of surgery: All patients who do not achieve pCR are recommended to receive therapy with trastuzumab emtansine (T-DM1) for 14 doses in the post-operative setting. Patients with substantial residual disease (i.e. RCB III) or particularly high risk should also receive additional adjuvant chemotherapy. In this case, four cycles of adjuvant adriamycin/cyclophosphamide (AC) is recommended as a preferred adjuvant chemotherapy regimen. Patients with a small to moderate burden of residual disease (i.e. RCB I-II) may opt to receive T-DM1 alone. For patients with HR+ disease, any regimen of adjuvant hormonal therapy may be given at the investigator's discretion. Receipt of adjuvant bone modifying agents will not be considered in the assessment of escalated or de-escalated adjuvant therapy.

Based on pCR rate of 45.8% for HER2+ breast cancer patients treated with taxane chemotherapy/trastuzumab/pertuzumab in the NeoSphere trial,[35] it is expected that roughly half of patients enrolled on this trial will not achieve pCR and will go on to

receive additional adjuvant chemotherapy.

5.14.3 Post-operative hormonal therapy: Specific regimen and duration of post-operative hormonal therapy should be delivered at the investigator's discretion.

5.14.4 Recording of systemic adjuvant chemotherapy planned: At the post-operative visit, all efforts should be made for the patient and provider to review surgical pathology results and discuss a plan for adjuvant chemotherapy. pCR status and planned adjuvant chemotherapy (or lack thereof) should be recorded in the CRF and the questionnaire regarding planned adjuvant chemotherapy (see Questionnaire Packet: Appendix F) must be completed by a member of the study team, and signed by the investigator. If for any reason the discussion of planned adjuvant chemotherapy is not completed at the post-operative visit, CRF recording of planned non-hormonal adjuvant systemic therapy and completion of the questionnaire may be delayed until a final decision is made regarding planned adjuvant chemotherapy, though it is strongly encouraged that this decision be made within 28 days (and as soon as possible) after the post-operative visit. Every effort must be made to complete adjuvant chemotherapy decision-making at an in-person visit. This determination of planned adjuvant chemotherapy is necessary for real-time administration of patient questionnaires (see Section 5.15).

5.14.5 Recording of adjuvant chemotherapy received (TRIAL PRIMARY ENDPOINT): At approximately 12 weeks after the post-operative visit, or as close to that time interval as possible (and no less than 10 weeks or more than 20 weeks), each patient's chart will be reviewed to assess whether they received further adjuvant chemotherapy, or not, and this information (including yes/no, and if yes, regimen received) will be recorded in the CRF.

5.14.6 Recording of all systemic adjuvant therapy received: Over the course of adjuvant systemic therapy, the patient record should be regularly reviewed and adjuvant systemic therapy received (including hormonal therapy, targeted therapy, and bone modifying agents) should be recorded and updated as of the most recent review timepoint. As in Section 5.17 "Duration of Follow-up," every effort should be made for these assessments to occur at least every 6 months until 5 years after surgery, then at least yearly until 10 years after surgery, or until patient death (if occurring sooner than 10 years).

Of note, though these represent targets for follow-up data gathering interval and timespan, depending on availability of funding, the interval and duration of record review in follow-up may change.

5.15 Assessment of Adjuvant Systemic Therapy Decision-making

5.15.1 Designation of adjuvant systemic therapy status

Based on pCR status (yes/no) plus systemic adjuvant therapy planned, as defined and recorded per Section 5.14, patients will be designated as belonging to one of three categories: (1) non-de-escalator; (2) unplanned-de-escalator; (3) protocol-consistent adjuvant therapy. Of note, in all patients with HR+ disease, any regimen of adjuvant hormonal therapy may be given at the investigator's discretion (and will not be considered in the assessment of escalation/de-escalation). Adjuvant bisphosphonate use will also not be considered in the assessment of escalation/de-escalation. See Table below:

	pCR: Yes	pCR: No
**Plan adjuvant HP or T-DM1 alone** (+/- hormonal therapy)	Protocol-consistent adjuvant therapy	***Unplanned-de-escalator***
**Plan adjuvant HP or T-DM1 + additional adjuvant chemotherapy** (+/- hormonal therapy)	***Non-de-escalator***	Protocol-consistent adjuvant therapy

As per Section 5.14.1: This designation will be established at the time of the post-operative medical oncology visit, and recorded in the CRF. The planned adjuvant chemotherapy questionnaire (Questionnaire Packet: Appendix F; to be completed by a study team member) will also be completed at that time. If for any reason the discussion of planned adjuvant chemotherapy is not completed at the post-operative visit, CRF recording of planned adjuvant chemotherapy may be delayed until a final decision is made regarding planned adjuvant chemotherapy, though it is strongly encouraged that this decision be made within 28 days (and as soon as possible) after the post-operative visit.

5.15.2 Patient questionnaire completion for non-de-escalator patients and unplanned-de-escalator patients

For patients designated to fall in the non-de-escalator and unplanned-de-escalator groups, a questionnaire should be administered at the timepoint at which this designation is made. The goal of these questionnaires is to assess the rationale behind planning for non-protocol-consistent adjuvant therapy.

Questionnaire for non-de-escalator patients, see Appendix A of the Questionnaire Packet.

Questionnaire for unplanned-de-escalator patients, see Appendix B of the Questionnaire Packet.

5.15.3 Patient questionnaire completion for protocol-consistent adjuvant therapy patients

For patients designated to fall in the protocol-consistent adjuvant therapy group, a questionnaire should be administered at the timepoint at which this designation is made.

This questionnaire can be found in Appendix E of the Questionnaire Packet.

5.15.4 Structured physician medical record review for non-de-escalator patients and unplanned-de-escalator patients

Following patient designation into the non-de-escalator or unplanned-de-escalator groups, medical record review of all notes relating to adjuvant chemotherapy decision-making (meaning notes filed prior to the final, recorded decision being made) will be performed by at least one physician member of the study team. Every effort will be made for the same physician study team member to review all charts, for consistency. External sites must submit a printed copy of a patient's post-operative visit note to ctopm@dfci.harvard.edu. The physician study team member will record, using the same structure of responses as provided on patient questionnaires, their interpretation of the reasons for planning a non-protocol-consistent adjuvant therapy regimen. For each case, this will be recorded on a structured physician review form. Every effort will be made for each record to be independently reviewed and questionnaire completed by two physicians, in order to ensure validity of results as much as possible.

Structured physician review form for non-de-escalator patients, see Appendix C of the Questionnaire Packet.

Structured physician review form for unplanned-de-escalator patients, see Appendix D of the Questionnaire Packet.

5.16 Criteria for Taking a Participant Off Protocol Therapy

Treatment will continue for 4 cycles in the neoadjuvant setting, or until one of the following criteria applies:

- Disease progression
- Intercurrent illness that prevents further administration of treatment
- Unacceptable adverse event(s)
- Participant demonstrates an inability or unwillingness to comply with the medication regimen and/or documentation requirements
- Participant decides to withdraw from the protocol therapy
- General or specific changes in the participant's condition render the participant unacceptable for further treatment in the judgment of the treating investigator

Participants will be removed from the protocol therapy when any of these criteria apply. The reason for removal from protocol therapy, and the date the participant was removed, must be documented in the case report form (CRF). Alternative care options will be discussed with the participant.

When a participant is removed from protocol therapy and/or is off of the study, the relevant Off-Treatment/Off-Study information will be updated in OnCore.

In the event of unusual or life-threatening complications, treating investigators must immediately notify the Overall PI, Adrienne Waks, MD, at 617-632-3800 or awaks@partners.org.

5.17 Duration of Follow-Up

The post-surgery follow-up visit at which a final decision is made about whether or not the patient will receive further adjuvant chemotherapy will be considered the subject's final study visit (regardless of whether or not the patient goes to surgery immediately following protocol therapy, or receives additional preoperative therapy). It is expected that this will constitute the first post-surgery follow-up visit for the vast majority of patients. If breast surgery is not performed the participant's final visit should be within 28 days after the last dose of protocol-specified therapy Comprehensive information about post-surgical treatment received will be subsequently collected. See Section 5.14.6.

Every effort should be made for follow-up assessments to occur at least every 6 months until 5 years after surgery, then at least yearly until 10 years after surgery, or until patient death (if occurring sooner than 10 years).

Event-free survival (EFS) will be defined from the time of registration until the occurrence of the first of the following events:

- Local/regional recurrence: a recurrent or new invasive ipsilateral breast cancer, invasive breast cancer in the axilla, regional lymph nodes, chest wall, or skin of the ipsilateral breast.
- Contralateral invasive breast cancer,
- Distant recurrence: metastatic disease that has either been biopsy confirmed or clinically diagnosed as recurrent invasive breast cancer. A single new lesion on a bone scan without evidence of lytic disease on x-ray and without symptoms does not in and of itself constitute distant recurrence, but multiple new bone lesions, or increased isotope uptake associated with new bone symptoms are more likely due to metastases. Bone metastases must be documented with x-rays and clinical description.
- Death from any cause

In situ cancer is not included as EFS event. If a patient has in situ breast cancer (on the ipsilateral or contralateral side) diagnosed during follow-up before any of the EFS events above, then the patient should continue to be followed for EFS on study (even if she is given hormonal therapy or undergoes additional surgery after the in situ diagnosis). These patients will be followed for survival.

If a patient is diagnosed with a non-melanoma skin cancer or a vaginal carcinoma in situ, she will continue on this study and continue to be followed for EFS.

Disease-free survival (DFS) will be defined for patients who undergo surgery for breast cancer as the interval from the time of surgery until the occurrence of EFS events noted above.

Recurrence-free interval will be defined for patients who undergo surgery for breast cancer as the interval from the time of surgery until the occurrence of invasive local/regional recurrence (as defined in EFS events, above), distant recurrence (as defined in EFS events, above), or death from breast cancer.

It is recommended that any disease-free survival event should be biopsied to confirm recurrent disease. Every effort will be made to collect information on breast cancer status, new anti-cancer therapy, and new onset malignancy diagnoses via simplified CRFs. Following a DFS event, survival information (i.e., date and cause of death or last known alive date if not deceased and new onset malignancy information) should be collected.

Lastly, post-surgical blood draws for correlative trial analyses will be performed in the one-year post-operative follow-up period, as described in Section 9.3.

5.18 Criteria for Taking a Participant Off Study

Participants will be removed from study before study completion when any of the following criteria apply:

- Lost to follow-up
- Withdrawal of consent for data submission
- Death

The reason for taking a participant off study, and the date the participant was removed, must be documented in the case report form (CRF).

When a participant is removed from protocol therapy and/or is off of the study, the relevant Off-Treatment/Off-Study information will be updated in OnCore.

In the event of unusual or life-threatening complications, treating investigators must immediately notify the Overall PI, Adrienne Waks, MD, at 617-632-3800 or awaks@partners.org.

6. DOSING DELAYS/DOSE MODIFICATIONS

Paclitaxel/trastuzumab/pertuzumab (THP) is part of a standard, guideline-backed regimen[31] for the neo/adjuvant treatment of non-metastatic HER2+ breast cancer. This triplet combination, or the combination of HP with the related taxane docetaxel, has already been studied and associated toxicity and efficacy results reported in multiple prospective clinical trials.[35,38,47] Accordingly, all toxicities experienced by patients on this protocol may be managed at the investigator's discretion according to institutional guidelines, with dosing delays and dose modifications instituted by the investigator based on their clinical experience with the regimen and clinical judgment for a given patient receiving this standard-of-care therapy.

The overall PI of the study may be contacted at any point if an investigator has a question about management of a toxicity that arises on study treatment.

Since the overarching goal of the protocol is to evaluate de-escalation of therapy following pathologic complete response to neoadjuvant THP, <u>all efforts should be made to maximize THP dosing and achieve completion of the neoadjuvant THP regimen</u> in as many patients as possible. Therefore, though the standard regimen as written in this protocol has a 12 week duration, <u>it will be acceptable to make up missed doses of any component of the regimen through 14 weeks.</u> In addition, if a patient is able to complete only 10 doses of paclitaxel (plus all 4 doses of HP) in the neoadjuvant setting, and achieves pCR at surgery, that patient may be treated in the adjuvant setting as if s/he had completed all 12 doses of neoadjuvant weekly paclitaxel (as described in

Section 5.14.1).

As described further in Section 7, dose delay for toxicity, dose modification for toxicity, doses missed for toxicity, and total neoadjuvant THP doses completed should be recorded on study CRFs. In the case of delayed, modified, or missed doses, the reason may also be recorded, with the descriptions and grading scales found in the revised NCI Common Terminology Criteria for Adverse Events (CTCAE) version 4.0. A copy of the CTCAE version 4.0 can be downloaded from the CTEP website http://ctep.cancer.gov/protocolDevelopment/electronic_applications/ctc.htm.

7. ADVERSE EVENTS: LIST AND REPORTING REQUIREMENTS

As described in Section 6, since paclitaxel/trastuzumab/pertuzumab (THP) is part of a standard, guideline-backed, Food and Drug Administration (FDA)-approved regimen for the neo/adjuvant treatment of non-metastatic HER2+ breast cancer, and has already been studied in prospective clinical trials with associated published safety data,[22,31,38] **not all adverse events occurring on trial will be routinely recorded on CRFs**, and safety is not a defined trial endpoint**. Any adverse event leading to a delayed, modified, or missed dose of any component of the neoadjuvant THP regimen may be recorded.**

Adverse events will be followed and recorded, as described, while study subjects complete the neoadjuvant portion of the THP regimen only. Grade 5 events, regardless of expectedness or relationship to study treatment, will be followed and reported up to 30 days after the last dose of treatment on the neoadjuvant THP regimen. Otherwise, adverse events that occur after a patient's receipt of final therapy infusions on the neoadjuvant THP regimen will not be followed or recorded.

7.1 Expected Toxicities

7.1.1 Adverse Event List(s) for Trastuzumab

Signs and symptoms of cardiac dysfunction, such as dyspnea, increased cough, paroxysmal nocturnal dyspnea, peripheral edema, S3 gallop, or reduced ejection fraction, have been observed in participants treated with trastuzumab. Congestive heart failure associated with trastuzumab therapy may be severe and has been associated with disabling cardiac failure, death, and mural thrombosis leading to stroke. The probability of cardiac dysfunction was highest in participants who received trastuzumab concurrently with anthracyclines.

An increased incidence of anemia and leukopenia was observed in the treatment group receiving trastuzumab and chemotherapy, especially in the trastuzumab and adriamycin/cytoxan subgroup, compared with the treatment group receiving chemotherapy alone. The majority of these cytopenic events were mild or moderate in intensity, reversible, and none resulted in discontinuation of therapy with trastuzumab. Hematologic toxicity is infrequent following the administration of trastuzumab as a single agent, with an incidence of Grade III toxicities for WBC, platelets, hemoglobin all <1%.

No Grade IV toxicities were observed.

Of participants treated with trastuzumab as a single agent, 25% experienced diarrhea. An increased incidence of diarrhea, primarily mild to moderate in severity, was observed in participants receiving trastuzumab in combination with chemotherapy.
An increased incidence of infections, primarily mild upper respiratory infections of minor clinical significance or catheter infections was observed in participants receiving trastuzumab in combination with chemotherapy.

During the first infusion with trastuzumab, a symptom complex most commonly consisting of chills and/or fever was observed in about 40% of participants in clinical trials. Other signs and/or symptoms may include nausea, vomiting, pain (in some cases at tumor sites), rigors, headache, dizziness, dyspnea, hypotension, rash and asthenia. The symptoms occurred infrequently with subsequent trastuzumab infusions.

7.1.2 Adverse Event List(s) for Paclitaxel

Myelosuppression, liver function test abnormalities (elevated SGOT, SGPT, bilirubin, alkaline phosphatase), nausea, vomiting, diarrhea, mucositis, peripheral neuropathy, transient asymptomatic bradycardia, and with much less frequency, arrhythmias, hypotension, hypersensitivity/anaphylaxis reactions (dyspnea, tachycardia, rash, urticaria, hypotension, or hypertension), myalgias, arthralgias, and alopecia have been observed in patients receiving paclitaxel.

7.1.3 Adverse Event List(s) for Pertuzumab

Overall, data indicate that pertuzumab is well-tolerated as monotherapy and that it can be given in combination with trastuzumab and a range of other therapeutic agents with manageable additional toxicity. No new or unexpected toxicities have been encountered other than those that are known for agents that target the HER family of receptors.
Serious or severe infusion-associated symptoms have been rarely observed in patients receiving pertuzumab. A low level of cardiac toxicities, predominantly asymptomatic declines in left ventricular ejection fraction (LVEF), has been reported. In the pivotal Phase III trial WO20698/TOC4129g the rates of symptomatic and asymptomatic left ventricular systolic dysfunction (LVSD) were not higher in patients receiving pertuzumab, trastuzumab and docetaxel than in those receiving placebo, trastuzumab and docetaxel.

No fetal studies in humans have been performed but pertuzumab caused oligohydramnios, delayed renal development and embryo-fetal deaths in pregnant cynomolgus monkeys. Moreover, in the post-marketing setting, cases of oligohydramnios, some associated with fatal pulmonary hypoplasia of the fetus, have been reported in pregnant women receiving trastuzumab. Therefore, pertuzumab should not be used in pregnant women. Protocols for ongoing pertuzumab studies indicate that one highly effective or two effective contraceptive measures must be used; continuous

pregnancy monitoring must be performed during the trials and for six months after the last dose of pertuzumab is administered. Because of the long half-life of pertuzumab women should be warned not to become pregnant for at least seven months after completion of treatment.

7.2 Adverse Event Characteristics

- **CTCAE term (AE description) and grade:** The descriptions and grading scales found in the revised NCI Common Terminology Criteria for Adverse Events (CTCAE) version 4.0 will be utilized for AE reporting. All appropriate treatment areas should have access to a copy of the CTCAE version 4.0. A copy of the CTCAE version 4.0 can be downloaded from the CTEP web site http://ctep.cancer.gov/protocolDevelopment/electronic_applications/ctc.htm.

- **For expedited reporting purposes only:**
  - AEs for the protocol that do not require expedited reporting are outlined in the next section (Expedited Adverse Event Reporting) under the sub-heading of Protocol-Specific Expedited Adverse Event Reporting Exclusions.

- **Attribution** of the AE:
  - Definite – The AE *is clearly related* to the study treatment.
  - Probable – The AE *is likely related* to the study treatment.
  - Possible – The AE *may be related* to the study treatment.
  - Unlikely – The AE *is doubtfully related* to the study treatment.
  - Unrelated – The AE *is clearly NOT related* to the study treatment.

7.3 Expedited Adverse Event Reporting

7.3.1 Events requiring expedited adverse event reporting are only those that result in a death, that is at least possibly related to agents received on this protocol. The only regulatory bodies who need to receive SAE reports are local IRBs and the DF/HCC IRB. There is no reporting to the FDA or drug companies in the context of this protocol.

7.3.2 For Multi-Center Trials where a DF/HCC investigator is serving as the Sponsor, each participating institution **must** abide by the reporting requirements set by the DF/HCC. This applies to any grade 5 toxicity (death) regardless of attribution.

7.3.3 DF/HCC Expedited Reporting Guidelines

Given the well-known safety profile of this FDA-approved regimen, in the context of this protocol, only grade 5 adverse events will be reported to the DFCI IRB.

Other investigative sites will report AEs to their respective IRB according to the local

IRB's policies and procedures in reporting adverse events. A copy of the submitted institutional AE form should be forwarded to the Overall PI within 24 hours..

The Overall PI will submit AE reports from outside institutions to the DFCI OHRS according to DFCI IRB policies and procedures in reporting adverse events.

7.3.4 Protocol-Specific Expedited Adverse Event Reporting Exclusions

THP is part of a commercially available, guideline-backed, FDA-approved regimen for the neo/adjuvant treatment of non-metastatic HER2+ breast cancer. Safety data for the combination is well-documented and as a result is not a defined trial endpoint.[22,31,38] For this protocol only, all grade 5 events regardless of expectedness or relationship to study treatment will be reported to the Overall PI or the DFCI IRB and entered into the case report forms. No other events require reporting.

7.4 Expedited Reporting to Hospital Risk Management

Participating investigators will report to their local Risk Management office any participant safety reports or sentinel events that require reporting according to institutional policy.

7.5 Routine Adverse Event Reporting

All Adverse Events necessitating modified, delayed, or missed doses must be reported in routine study data submissions to the Overall PI on the toxicity case report forms. **AEs reported through expedited processes (e.g., reported to the IRB, FDA, etc.) must also be reported in routine study data submissions.**

8. PHARMACEUTICAL INFORMATION

A list of the adverse events and potential risks associated with the agents administered in this study can be found in Section 7.1.

All pharmaceutical agents will be obtained from commercial institutional supply, therefore slight modifications to the below guidelines are allowable so long as local institutional policy is followed.

8.1 Trastuzumab

8.1.1 Description, Form, and Preparation

This study will use trastuzumab from commercial supply. Trastuzumab is a sterile, white to pale yellow, preservative free lyophilized powder for intravenous (IV) administration.

150 mg single-dose vial:

Each single-dose vial of Herceptin delivers 150 mg trastuzumab, 136.2 mg α,α-trehalose dihydrate, 3.4 mg L-histidine HCl monohydrate, 2.2 mg L-histidine, and 0.6 mg polysorbate 20.
Use appropriate aseptic technique. Reconstitute each 150 mg vial of single-dose Trastuzumab with 7.4 mL of sterile water for injection (SWFI) to yield a solution containing 21mg/mL trastuzumab that delivers 7.15 mL (150 mg trastuzumab), at a pH of approximately 6.
Use the trastuzumab solution immediately following reconstitution with SWFI, as it contains no preservative and is intended for single-dose only. If not used immediately, store the reconstituted trastuzumab solution for up to 24 hours at 2○C to 8○C (36○F to 46○F); discard any unused trastuzumab after 24 hours.
Use of other reconstitution diluents should be avoided. Determine the dose of trastuzumab needed, based on a loading dose of 8 mg trastuzumab/kg body weight for q3wk dosing schedules or a maintenance dose of 6 mg/kg trastuzumab/kg body weight for q3w dosing schedules. Calculate the correct dose using 21 mg/mL trastuzumab solution. Withdraw this amount from the vial and add it to an infusion bag containing 250 mL of 0.9% sodium chloride, USP. **DEXTROSE (5%) SOLUTION SHOULD NOT BE USED**. Gently invert the bag to mix the solution. The reconstituted preparation results in a colorless to pale yellow transparent solution. Parenteral drug products should be inspected visually for particulates and discoloration prior to administration.

Trastuzumab should not be mixed or diluted with other drugs. Trastuzumab infusions should not be administered or mixed with Dextrose solutions. Trastuzumab should not be filtered during administration.

Refer to the Investigator's Brochure for detailed trastuzumab information and FDA approved package insert for more information.

8.1.2 Storage and Stability

Vials of trastuzumab are stable at 2ºC–8ºC (36ºF–46ºF) prior to reconstitution. Do not use beyond the expiration date stamped on the vial. A vial of 440 mg trastuzumab reconstituted with BWFI is stable for 28 days after reconstitution when stored refrigerated at 2ºC–8ºC (36ºF–46ºF), and the solution is preserved for multiple use. Discard any remaining multi dose reconstituted solution after 28 days. If unpreserved SWFI (not supplied) is used, the reconstituted trastuzumab solution should be used immediately and any unused portion must be discarded. A vial of 150 mg trastuzumab once reconstituted with SWFI should be used immediately, as it contains no preservative and is intended for single-dose only. If not used immediately, store the reconstituted trastuzumab solution for up to 24 hours at 2°C–8°C; discard any unused trastuzumab after 24 hours.

DO NOT FREEZE TRASTUZUMAB THAT HAS BEEN RECONSTITUTED.

The solution of trastuzumab for infusion diluted in polyvinylchloride or polyethylene bags containing 0.9% sodium chloride for injection, USP, may be stored at 2ºC–8ºC (36ºF–46ºF) for up to 24 hours prior to use. Diluted trastuzumab has been shown to be stable for up to

24 hours at room temperature 15ºC–25ºC; however, since diluted trastuzumab contains no effective preservative the reconstituted and diluted solution should be stored refrigerated (2ºC–8ºC).

8.1.3 Compatibility

No incompatibilities between trastuzumab and polyvinylchloride, polyolefin or polypropylene bags have been observed. Dextrose 5% solution should not be used since it causes aggregation of the protein. Trastuzumab should not be mixed or diluted with other drugs.

8.1.4 Handling

Qualified personnel, familiar with procedures that minimize undue exposure to themselves and the environment, should undertake the preparation, handling, and safe disposal of the chemotherapeutic agent in a self-contained and protective environment.

8.1.5 Availability

Trastuzumab is a commercially available agent. The cost of trastuzumab will be charged to the patient and/or his/her insurance company since its use is considered standard of care for neoadjuvant treatment of HER2-positive breast cancer.

8.1.6 Administration

Trastuzumab will be administered over 30-90 minutes, or as per local institutional guidelines. Patients should be observed for fever and chills or other infusion-associated symptoms for at least 60 minutes after the initial, or as per local institutional guidelines. Refer to Section 5.1 and Section 5.3 for more details.

8.1.7 Ordering

As trastuzumab will be used as a commercially-supplied agent, ordering will be at the discretion of each local institution pharmacy's standard practices.

8.1.8 Drug Supplies and Accountability

As commercial supply of trastuzumab will be used, it will be supplied and the inventory kept according to local institutional pharmacy practices.

8.1.9 Destruction and Return

Unused supplies of trastuzumab should be destroyed according to institutional policies.

8.2 Paclitaxel

8.2.1 Description and Form

Other Names
Taxol (NSC 125973)

Classification
Antimicrotubule agent.

Mode of Action
Promotes microtubule assembly and stabilizes tubulin polymers by preventing their depolarization, resulting in the formation of extremely stable and nonfunctional microtubules, and consequently inhibition of many cell functions.

8.2.2 Preparation

Preparation of paclitaxel should follow each institutional guideline, and may slightly deviate from the suggested preparations guidelines in the remainder of this paragraph to accommodate different institutional guidelines. Paclitaxel may be diluted in 0.9% sodium chloride injection, USP or 5% dextrose injection, USP. Paclitaxel must be prepared in glass, plypropylene or plyolefin containers and non-PVC containing (nitroglycerin) infusion sets. In-line filtration with a 0.22 micron filter is required.

8.2.3 Storage and Stability

Intact vials should be stored between 20° - 25° C (68° - 77° F) in the original package to protect from light, and remain stable until the expiration date on the label. Neither freezing nor refrigeration adversely affects stability. Upon refrigeration components in the paclitaxel vial may precipitate, but will redissolve upon reaching room temperature with little or no agitation. There is no impact on product quality under these circumstances. If the solution remains cloudy or if an insoluble precipitate is noted, the vial should be discarded. Solutions for infusion prepared as recommended are stable at ambient temperature (approximately 25° C) and lighting conditions for up to 27 hours.

8.2.4 Compatibility

Avoid the use of PVC bags and infusion sets due to leaching of DEHP (plasticizer). Ketoconazole may inhibit paclitaxel metabolism, based on *in vitro* data. Prescription of concomitant drugs should address the Launch Lexi-Interact™ Drug Interactions Program.

8.2.5 Handling

Qualified personnel, familiar with procedures that minimize undue exposure to themselves and the environment, should undertake the preparation, handling, and safe disposal of the chemotherapeutic agent in a self-contained and protective environment.

8.2.6 Availability

Paclitaxel is commercially available agent. Each institutional pharmacy should assure availability for the study.

8.2.7 Administration

Paclitaxel will be administered over 30-180 minutes, or as per local institutional guidelines. Refer to Section 5.1 and Section 5.3 for more details.

8.2.8 Ordering

As paclitaxel will be used as a commercially-supplied agent, ordering will be at the discretion of each local institution pharmacy’s standard practices.

8.2.9 Drug Supplies and Accountability

As commercial supply of paclitaxel will be used, it will be supplied and the inventory kept according to local institutional pharmacy practices.

8.2.10 Destruction and Return

Unused supplies of paclitaxel should be destroyed according to institutional policies.

8.3 Pertuzumab

8.3.1 Description, Form, and Preparation

Pertuzumab drug product is provided as a single use formulation containing 30 mg/mL pertuzumab in 20 mM L-histidine acetate (pH 6.0), 120 mM sucrose and 0.02%

polysorbate 20. Each 20 mL vial contains 420 mg of Pertuzumab (14.0 mL/vial). **DEXTROSE (5%) SOLUTION SHOULD NOT BE USED.** The preparation of pertuzumab solution for infusion, using aseptic technique, should be as follows:

- Parenteral drug products should be inspected visually for particulates and discoloration prior to administration.
- Withdraw the appropriate volume of pertuzumab liquid concentrate from the vial(s).
- Dilute into the 250 mL 0.9% sodium chloride PVC or non PVC polyolefin infusion bags. Do not withdraw saline out of the infusion bag.
- Mix diluted solution by gentle inversion. Do not shake.
- Administer immediately once prepared.

Refer to the Investigator's Brochure for detailed pertuzumab information and FDA approved package insert for more information.

8.3.2 Storage and Stability

Upon receipt, pertuzumab vials are to be refrigerated at 2°C–8°C (36°F–46°F) until use. Pertuzumab vials should not be used beyond the expiration date provided by the manufacturer. Because the formulation does not contain a preservative, the vial seal may only be punctured once. Any remaining solution should be discarded. Vial contents should be protected from light, and should not be frozen or shaken. The solution of pertuzumab for infusion, diluted in PVC or non-PVC polyolefin bags containing 0.9% Sodium Chloride Injection, USP, may be stored at 2°C–8°C for up to 24 hours prior to use. Diluted pertuzumab has been shown to be stable for up to 24 hours (up to 30°C). However, since diluted pertuzumab contains no preservative, the diluted solution should be stored refrigerated (2°C–8°C).

8.3.3 Compatibility

No incompatibilities between pertuzumab and polyvinylchloride, polyethylene or non-PVC polyolefin bags have been observed. **Dextrose (5%) in water (D5W) solution should not be used to dilute pertuzumab** since it was chemically and physically unstable in such solutions (dilute formulations of pertuzumab liquid formulations in D5W IV bags did not maintain stable pH after storage at room temperature (27-33°C) for 24 hours followed by 24 hours at refrigerator temperature [2-8°C]). Pertuzumab should not be mixed or diluted with other drugs.

8.3.4 Handling

Qualified personnel, familiar with procedures that minimize undue exposure to themselves and the environment, should undertake the preparation, handling, and safe disposal of the chemotherapeutic agent in a self-contained and protective environment.

8.3.5 Availability

Pertuzumab is a commercially available agent. The cost of pertuzumab will be charged to the patient and/or his/her insurance company since its use is considered standard of care for neoadjuvant treatment of HER2-positive breast cancer.

8.3.6 Administration

Pertuzumab will be administered over 30-60 minutes, or as per local institutional guidelines. Patients should be observed for fever and chills or other infusion-associated symptoms for at least 60 minutes after the initial dose and for 30 minutes after subsequent doses, or as per local institutional guidelines. Refer to Section 5.1 and Section 5.3 for more details.

8.3.7 Ordering

As pertuzumab will be used as a commercially-supplied agent, ordering will be at the discretion of each local institution pharmacy's standard practices.

8.3.8 Drug Supplies and Accountability

As commercial supply of pertuzumab will be used, it will be supplied and the inventory kept according to local institutional pharmacy practices.

8.3.9 Destruction and Return

Unused supplies of pertuzumab should be destroyed according to institutional policies.

9. BIOMARKER, CORRELATIVE, AND SPECIAL STUDIES

Breast tumor tissue sampling is required at baseline and at the time of surgery for all patients enrolled on this trial. For patients with obvious residual disease following completion of neoadjuvant THP, additional systemic therapy may be given in the neoadjuvant setting (see Section 5.4). In this case, a biopsy performed at the time of treatment change, prior to initiation of new anti-cancer therapy, is strongly recommended, but not required. Tissue will be used for profiling of the immune microenvironment, as detailed below. Additionally, we will bank blood and tissue specimens for possible future genomic sequencing analysis, and other further testing.

If sufficient tissue for correlative testing is not available from the baseline biopsy or surgical collection specimens collected, the assays may be attempted on the patient's banked tumor tissue, if feasible.
An optional research biopsy and blood collection at the time of breast cancer recurrence may be obtained for patients who consent (and, in the case of biopsy, have biopsy-accessible tumor). These tissue and blood samples will undergo the same collection protocol and testing as described below.

Blood collections will be performed at 4 timepoints on trial (one baseline timepoint plus 3 on-treatment timepoints) as detailed below. Blood will be used for profiling of peripheral blood mononuclear cells (PBMCs) and banked for future sequencing of circulating tumor DNA (ctDNA) and other future assays of interest.

Summary table: sample collection

**Research Sampling**	**Timepoint** *(more details follow)*	**Contents**	**Destination**
Fresh tissue	Baseline	5-7 cores	Laboratory of Elizabeth Mittendorf MD, PhD***
	Prior to neoadjuvant therapy switch*	5-7 cores	
	Surgery	4 core-sized pieces of tissue from residual tumor or tumor bed	
	Breast Cancer Recurrence**	5-7 cores	
Archival Tissue	Baseline and Surgical Tissue	FFPE Block and 1 H&E **OR** 10 unstained slides cut at 4-5um and 1 H&E	
Blood	Baseline (≤14 days prior to registration or ≤14 days prior to C1D1)	3 – 10 mL Streck tubes (ctDNA)<br>4 – 10 mL CPT tubes (PBMCs)	
	Preoperative (≤6 weeks prior to surgery; can be drawn just prior to last infusion)	3 – 10 mL Streck tubes (ctDNA)<br>4 – 10 mL CPT tubes (PBMCs)	
	Postoperative (≤6 weeks post-surgery)	3 – 10 mL Streck tubes (ctDNA)<br>4 – 10 mL CPT tubes (PBMCs)	
	Final 3 months of adjuvant HP (between 13th overall HP dose and end of HP treatment)	3 – 10 mL Streck tubes (ctDNA)<br>4 – 10 mL CPT tubes (PBMCs)	
	Breast Cancer	3 – 10 mL Streck	

	Recurrence**	tubes (ctDNA)<br>4 – 10 mL CPT tubes (PBMCs)	

* ONLY for patients with obvious residual disease at the completion of neoadjuvant THP, for whom additional neoadjuvant systemic therapy is planned. This biopsy is strongly recommended but not required in these patients.
**Optional
*** Handling and Shipping: Processing and shipping of the blood and tissue samples after they are obtained is described in the study operations manual accompanying this protocol.

9.1 Hypotheses

The central hypothesis of our correlative studies is that the immune microenvironment and patients' peripheral immune system will impact response to standard therapy with paclitaxel, trastuzumab and pertuzumab (THP).

Firstly, we hypothesize that the specific TIL phenotype and the T cell repertoire will impact response to therapy. To test this hypothesis, we will fully characterize the TIL phenotype in HER2+ breast tumor specimens obtained pre- and post-treatment with standard therapy to include chemotherapy + HER2-targeted therapy. In addition, characterization of the co-inhibitory molecules on T cells in the tumor microenvironment post-treatment may inform the design of subsequent studies incorporating immunotherapy agents and enrolling patients who do not achieve a pCR with standard chemotherapy + HER2-targeted therapy.

Secondly, we hypothesize that myeloid cells within the tumor microenvironment will impact the response to standard treatment (paclitaxel, trastuzumab, pertuzumab) administered neoadjuvantly to HER2+ breast cancer patients. To test this hypothesis, we will fully characterize myeloid cells in the tumor microenvironment of HER2+ breast tumor specimens obtained pre- and post-treatment with standard therapy. We anticipate that there will be a macrophage infiltrate which would justify additional preclinical work investigating the potential utility of targeting CSF-1, the cytokine that recruits macrophages to tumors. Agents targeting CSF-1 are already being evaluated in clinical trials.

Thirdly, we hypothesize that the presence and functional activity of NK cells will impact response to anti-HER2 antibody therapy. Antibody-dependent cell-mediated cytotoxicity (ADCC) is a known mechanism of action of monoclonal antibodies to include trastuzumab and pertuzumab. ADCC is mediated by cells such as NK cells that have an Fc-gamma receptor that can facilitate cross-linking of cell-bound antibodies by binding their Fc portion. To test our hypothesis, we will evaluate NK cells present in both the peripheral blood and tumor tissues in HER2+ patients before and after anti-HER2 antibody therapy.

9.2 Overview of tissue collection protocols

Obtaining formalin-fixed paraffin embedded tissue from diagnostic specimen
Following processing of diagnostic tissue, an archival FFPE block, or if a pathology department will not release a block, 10 unstained slides cut at 4-5um should be obtained. An H&E from this

block should also be requested. Shipping and handling is described in the study operations manual accompanying this protocol.

Collection of non-surgical breast biopsies

Research core biopsies of the primary breast lesion will be obtained at the baseline timepoint from all participants, less than or equal to 14 days prior to registration.

For patients with obvious residual disease at the completion of neoadjuvant THP, for whom additional neoadjuvant systemic therapy is planned, a repeat breast biopsy prior to change in neoadjuvant therapy is strongly recommended. This biopsy should ideally be obtained less than or equal to 14 days prior to the initiation of the post-THP neoadjuvant therapy.

It is mandatory that core biopsies be image-guided. A clip should be placed in the biopsy site at the time of the research biopsy if a clip was not placed during diagnostic biopsy. Pre- and post-procedure 90-degree lateral and craniocaudal mammogram is recommended to ensure that the correct lesion has been biopsied and to determine the relationship of the clip to the lesion that was visualized prebiopsy. Clip migration following biopsy has been reported and the distance from the original biopsy cavity can be measured[48]. If sufficiently far away from the biopsy cavity, then an addendum should be made to the report documenting that the clip should NOT be used to guide post-treatment tumor sampling. Experience with NeoALTTO and I-SPY1 suggest that taking four core biopsy samples from one area is feasible and acceptable to participants and ethics committees[49].

Ideally five core biopsies will be obtained at each timepoint, with the following order of specimen collection:

- First and second core: 10% neutral buffered formalin tube
- Third core: Flash frozen in OCT
- Fourth core: 10% neutral buffered formalin tube
- Fifth core: Flash frozen in OCT

If additional cores are obtained, they should be processed as follows:

- Sixth core: 10% neutral buffered formalin tube
- Seventh core: Flash frozen in OCT

For up to 10 patients enrolled at DFCI, in whom it is anticipated that at least 4 cores will be obtained, the order of specimen collection will be as follows:

- First core-sized piece: 10% neutral buffered formalin
- Second core-sized piece: proprietary fixative used for RNA sequencing
- Third core-sized piece: proprietary fixative used for RNA sequencing
- Fourth core-sized piece: Flash frozen in OCT
- Fifth core-sized piece: 10% neutral buffered formalin

If additional cores are obtained in these patients, they should be processed as for all other patients described above.

<u>Obtaining formalin-fixed paraffin embedded tissue from surgical specimen</u>
Following processing of surgical tissue, an archival FFPE block, or if a pathology department will not release a block, 10 unstained slides cut at 4-5um should be obtained. An H&E from this block should also be requested. Shipping and handling is described in the study operations manual accompanying this protocol.

<u>Collection of fresh tissue from surgical specimens</u>

At the time of definitive surgery, the pathologist or pathology assistant will take core-sized pieces of tissue from the tumor or residual tumor bed (goal is at least four core-sized pieces of tissue; more or less are allowable per protocol, though a minimum of four is strongly preferred). Every effort should be made to obtain the sample as soon as possible after the time of resection.

If surgery is taking place at a location where there is insufficient research infrastructure for the described tissue collection at the surgical timepoint, surgical specimens should not be collected. Instead, an archival FFPE block or 10 unstained slides, and an H&E from this block should be obtained.

Immediate handling for the four core-sized pieces is as follows, with the following order of specimen collection:

- First core-sized piece: 10% neutral buffered formalin
- Second core-sized piece: Flash frozen in OCT
- Third core-sized piece: 10% neutral buffered formalin
- Fourth core-sized piece: Flash frozen in OCT

<u>Handling and Shipping:</u> Processing and shipping of the fresh surgical specimens after they are obtained is described in the study operations manual accompanying this protocol.

9.3 Overview of blood collection protocols

Research blood collection is mandatory for all participants. The samples may be banked in the DF/HCC Clinical Trials Core Laboratory or in the laboratory of Elizabeth Mittendorf MD, PhD for these and future research purposes. These specimens will become the property of the DF/HCC.

Research blood draws should be collected as follows at the following timepoints:

Baseline (≤14 days prior to registration or ≤14 days prior to C1D1)

- 3-10 mL Streck Tubes for whole blood
- 4-10mL CPT tubes for whole blood

Preoperative timepoint (≤6 weeks pre-surgery, and as close to surgery as possible; can be drawn just prior to last infusion):

- 3-10 mL Streck Tubes for whole blood

- 4-10mL CPT tubes for whole blood

Postoperative timepoint (≤6 weeks post-surgery):

- 3-10 mL Streck Tubes for whole blood
- 4-10mL CPT tubes for whole blood

During the final 3 months of adjuvant HP/T-DM1 *(note: blood draw timeframe should be kept constant, even if patient does not complete HP/T-DM1 therapy)*

- 3-10 mL Streck Tube for whole blood
- 4-10mL CPT tubes for whole blood

Breast Cancer Recurrence – **optional**

- 3-10 mL Streck Tubes for whole blood
- 4-10mL CPT tubes for whole blood

Handling and Shipping: Processing and shipping of the blood samples after they are obtained is described in the study operations manual accompanying this protocol.

Of note, every effort will be made to coordinate research blood draws with clinically-indicated blood tests to minimize additional venipuncture. Research coordinators will track participants closely and make every effort to collect blood at required timepoints. However, if research blood draws are missed or fall outside window due to the following circumstance they will not be considered deviations from the protocol.

Unanticipated changes in treatment regimens and last-minute clinic visits. There are no plans to bring participants back to clinic solely for the purposes of a research blood draw. Instead, the study team should attempt to coordinate a missed research blood draw with the participant's next clinically indicated blood draw unless otherwise specified by the Overall PI. Collection of CPT tubes may be omitted at study sites that do not have the capacity for PBMC processing before shipment.

9.4 Planned assays

All of the below-mentioned analyses may be altered or added to based on updated best practices and major questions of interest in immuno-oncology and breast cancer biology in general at the time that correlative science is performed. Alternative technologies (based on evolving scientific knowledge or techniques) may be used. Some of the assays also may be eliminated, if they are no longer deemed relevant at the time that correlative science is performed.

9.4.1 Tissue-based analyses

9.4.1.1 Characterization of the immune microenvironment

Assays will be performed to characterize immunologic aspects of the tumor microenvironment. Potential assays include: 1) flow cytometry analyses performed on single cell suspensions evaluating immune markers characterizing various

lymphocyte (ie. T cells, B cells, NK cells) and myeloid (i.e. macrophages, dendritic cells) populations and 2) multiplex immunofluorescence imaging performed on FFPE tissue to identify various lymphocyte and myeloid populations and their spatial relationships. 3) Bulk and single cell RNA sequencing for profiling and to determine changes in RNA expression signatures. 4) Whole Exome, Whole Genome Sequencing.

9.4.1.2 Nanostring Technologies

Nanostring digital spatial profiling technology will also be used to assess tissue architecture features of the immune microenvironment at high resolution.

9.4.2 Blood-based analyses

The presence and functional activity of various immune cell populations will be determined using flow cytometry. Circulating tumor DNA (ctDNA) will undergo sequencing analysis at the timepoints outlined, and may be used to explore (1) the correlation between ctDNA at the preoperative timepoint and presence of pCR versus residual disease; (2) the correlation between ctDNA at a postoperative timepoint and long-term breast cancer outcomes; (3) mechanisms of response and resistance to HER2-directed therapy.

9.5 Sites Performing Correlative Studies and/or Data Analysis

We will be sharing de-identified biospecimens and data with the following entities for the purposes described above:

- Breast Tumor Immunology Laboratory (Elizabeth Mittendorf, MD, PhD)
- Brigham and Women's Hospital
- The Broad Institute of MIT
- Harvard Medical School
- Nanostring Technologies
- University of Pennsylvania (Jonni Moore, PhD)
- Parker Institute for Cancer Immunotherapy

9.6 Tissue and blood banking

All leftover tissue and blood will be banked in the appropriate blood/tissue bank (e.g., in the DF/HCC Clinical Trials Core Laboratory as per standard lab protocol), such that it can be used for additional or future analyses as needed.

10. STUDY CALENDAR

Assessments must be performed prior to administration of any study agent. Every reasonable effort should be made to administer study assessments and agents **within +/- 3 days** of the protocol-specified date, unless otherwise noted. **Assessment**	**Pre-Study**		**Cycle 1-4 [b]**			**Pre-Op Visit**	**Post-Op Visit**	**Follow-up [m]**
	**≤ 28 days of registration [a]**	**≤ 14 days of registration**	**D1**	**D8**	**D15**			
Medical history	X							
Physical exam[n]	X		X					
Concomitant medications	X		X			X	X	
ECOG PS	X							
Vital signs	X		X					
Height and Weight	X		Weight only					
Breast imaging [c]	X					X		
Axillary assessment [d]	X							
Whole-body staging [e]	X							
Laboratories[f]	X		X	X [f]	X [f]			
Serum B-HCG [g]		X						
Adverse Event evaluation			X			X	X	
LVEF assessment [h]	X							
Visit with breast surgeon	X					X[i]	X	
Document Planned Adjuvant Chemotherapy [j]							X	
Questionnaire Completion for Non-De-Escalator and Unplanned De-Escalator Patients [j]							X	
Document adjuvant therapy received [k]								X
Research blood		X[o]				X	X	X
Research biopsy [l]		X				X (at surgery)		

a: All screening tests should be completed within 28 days of registration, with the exception of breast and axillary imaging, which may be completed up to 42 days prior to registration, pregnancy test (only required in specified patients), which should be completed within 14 days of registration, and LVEF assessment

(which should be completed within 3 months prior to registration). Axillary ultrasound and/or biopsy do not need to be repeated if performed prior to the screening period.<br>b: Every effort should be made for study visits to occur on the day specified, +/- 3 days. See Section 5.1.<br>c: See Section 5.8.1.<br>d: See Section 5.10.<br>e: Whole-body staging recommended only in patients with stage III disease according to AJCC staging manual edition 8, anatomic staging table.<br>f: See patient eligibility for lab values that must be verified at the time of screening. Thereafter, comprehensive metabolic panel and complete blood count with differential should be drawn at day 1 of each cycle (see Section 5.2 for guidelines regarding acceptable lab values), and contents and timing of additional lab draws are at the discretion of the treating physician.<br>g: Pregnancy test only required in women of childbearing potential (within 14 days of registration).<br>h: LVEF Assessment may be either Echo or MUGA.<br>i: Pre-op visit with surgeon is recommended to occur any time on or after C4D8, but can be performed earlier at the discretion of the surgeon.<br>j: See Section 5.14.4 and Section 5.15.<br>k: Receipt of adjuvant chemotherapy (Y/N and regimen) should be assessed at approximately 12 weeks following the post-op visit. This assessment forms the basis for the primary endpoint of the trial (see Section 5.14.5). Receipt of additional adjuvant systemic therapy should be followed and recorded as per Section 5.14.6.<br>l: See Section 9.2.<br>m: See Sections 5.14 and 9.3.<br>n: See Section 5.6.<br>o: ≤14 days prior to registration or ≤14 days prior to C1D1.

11. MEASUREMENT OF EFFECT

11.1 Antitumor Effect – Solid Tumors

A baseline and presurgical radiographic study of the breast is required; MRI is recommended. The same radiographic modality should be used consistently if at all possible. The baseline scan must be obtained within 42 days prior to registration. The presurgical scan should occur a maximum of 4 weeks after the last neoadjuvant therapy. If the participant clinically progresses, repeat imaging is required. If there is discordance (clinical progression, but radiographic stable disease or response), and further input is desired, contact the overall study PI.

11.2 Radiographic assessment

Each participant will have pre- and post-therapy radiographic tumor measurements, preferably by MRI, however if logistic or practical issues preclude MRI use, mammogram or ultrasound may be substituted. The longest diameter (LD) of the target lesion at the time of study initiation will be reported as the baseline LD. The baseline LD of the target lesion may be used as reference to further characterize the objective tumor response of the measurable dimension of the disease.

Response criteria are based on the RECIST 1.1 criteria:

Radiographic Complete Response (CR): Complete disappearance of the target lesion

Radiographic Partial Response (PR): Greater than or equal to 30% decrease in the longest diameter (LD) of the target lesion taking as reference the baseline LD.

Radiographic Progressive Disease (PD): Greater than or equal to 20% increase in the LD of target lesion taking as reference the baseline LD or the appearance of one or more new lesions
.
Radiographic Stable Disease (SD): Neither sufficient shrinkage to qualify for PR nor sufficient increase to qualify for PD taking as reference the baseline LD

11.3 Clinical assessments

Both target and, in the event of multifocal or multicentric invasive cancer, nontarget lesions should be followed clinically and their clinical size recorded at baseline. Measurements thereafter are required; these lesions should be categorized at subsequent visits regarding whether there is evidence of progression. If "yes", the study chair may be notified in order to determine whether the participant should come off protocol treatment.

11.4 Pathologic Response

For the purpose of this study pCR will be defined as ypT0/is ypN0.

Pathologic response will also be reported using the Residual Cancer Burden calculator [1] from

M.D Anderson: http://www.mdanderson.org/breastcancer_RCB.

The following parameters are required from pathologic examination in order to calculate Residual Cancer Burden (RCB) after neoadjuvant treatment:

- The largest two dimensions (mms) of the residual tumor bed in the breast (largest tumor bed if multicentric disease)
- Histologic assessment of the percentage of the tumor bed area that contains carcinoma (all carcinoma, i.e. invasive and in situ), select one of the following: 0%, 1%, 5%, 10%, 20%, 30%, 40%, 50%, 60%, 70%, 80%, 90%
- To assess cellularity it is helpful to scan across the sections of tumor bed and then estimate the average cellularity from the different microscopic fields.
- When estimating percentage cancer cellularity in any microscopic field, compare the involved area with obvious standards, e.g. more or less than half, one quarter, one fifth, one tenth, one twentieth, etc.
- Expect there to be variable cellularity within the cross section of any tumor bed, but estimate the overall cellularity from the average of the estimates in different microscopic fields of the tumor bed. E.g., if cellularity in different fields of the tumor bed were estimated as 20%, 10%, 20%, 0%, 20%, 30%, then an average estimate of overall cellularity would be 20%.
- Histologic estimate of the percentage of the carcinoma in the tumor bed that is in situ, select one of the following: 0%, 1%, 5%, 10%, 20%, 30%, 40%, 50%, 60%, 70%, 80%, 90%, 100%
- The number of positive (metastatic) lymph nodes
- The largest diameter (mm) of the largest nodal metastasis

12. DATA REPORTING / REGULATORY REQUIREMENTS

Adverse event lists, guidelines, and instructions for AE reporting can be found in Section 7 (Adverse Events: List and Reporting Requirements).

12.1 Data Reporting

12.1.1 Method

The DAPHNe study team will collect, manage, and perform quality checks on the data for this study. Data entry will be performed using RedCap.

12.1.2 Responsibility for Data Submission

Investigative sites within DF/HCC or DF/PCC are responsible for submitting data and/or data forms via RedCap.

12.2 Data Safety Monitoring

A plan for continuous monitoring of unacceptable levels of recurrence, with associated prespecified stopping rules, is outlined in Section 13.4. As this is a non-high-risk pilot trial, it does not meet DF/HCC Data and Safety Monitoring Committee (DSMC) reporting criteria.

12.3 Multicenter Guidelines

This protocol will adhere to the policies and requirements of the DF/HCC Multi-Center Data and Safety Monitoring Plan. The specific responsibilities of the Overall PI, Coordinating Center, and Participating Institutions and the procedures for auditing are presented in Appendix C.

- Mechanisms will be in place to ensure quality assurance, protocol compliance, and adverse event reporting at each site.

12.4 Collaborative Research and Future Use of Data and Biospecimens

Tissue, blood, bodily fluids, and other materials derived from these will be collected in this study to analyze genes, DNA, RNA, proteins and cells for the study's correlative endpoints and potential future research, utilizing new types of biomarker testing as it becomes available.

These samples and any data generated as a part of this clinical trial may be used for future research studies and may be provided to collaborating investigators both within and outside of the DF/HCC for either correlative endpoints or secondary use. Samples and data may be shared with outside non-profit academic investigators, as well as with for-profit pharmaceutical investigators or commercial entities, with whom we collaborate. When samples or data are sent to collaborators and when any research is performed on them, all information will be identified with a code, and will not contain any PHI, such as name, birthday, or MRNs.

In order to allow the greatest amount of research to be performed on the specimens and information generated as a part of this trial, researchers in this study may share results of genetic sequencing with other scientists. De-identified specimen or genetic data may be placed into one of more publicly-accessible scientific databases, such as the National Institutes of Health's Database for Genotypes and Phenotypes (dbGaP). The results from the correlative research on this study will be shared with these public databases. Through such databases, researchers from around the world will have access to de-identified samples or data for future research. More detailed information, beyond the public database, may only be accessed by scientists at other research centers who have received special permission to review de-identified data.

13. STATISTICAL CONSIDERATIONS

13.1 Study Design

This is a single arm, single-stage evaluation of adjuvant treatment decisions in patients who achieve pCR with neoadjuvant paclitaxel/trastuzumab/pertuzumab. This open-label pilot trial will enroll patients with treatment-naïve stage II-III (according to AJCC staging manual

anatomic staging table, edition 8) HER2+ breast cancer and a minimum primary tumor size of 1.5 cm. Patients with any hormone receptor status will be eligible. Following registration, all patients will receive neoadjuvant paclitaxel/trastuzumab/pertuzumab (THP), and pathologic response will be assessed at surgery per standard clinical practice.

Patients with pathologic complete response (pCR; defined as ypT0/is N0) will complete one year of HP therapy in the adjuvant setting. Patients who do not achieve pCR will receive additional adjuvant chemotherapy of the investigator's choice, in addition to completing one year of HP in the adjuvant setting. In patients with hormone receptor-positive (HR+) disease, adjuvant hormonal therapy may be given at the investigator's discretion

All patients will be followed for recurrence and survival for up to 10 years from definitive surgery, as funding allows.

13.2 Endpoints

13.2.1 Primary Endpoint

The primary objective is to assess adherence to protocol-specified antibody doublet therapy (trastuzumab/pertuzumab only) in the adjuvant setting among patients who achieve pCR following neoadjuvant paclitaxel/trastuzumab/pertuzumab.

The primary endpoint is adjuvant chemotherapy received, as assessed at approximately 12 weeks following the post-operative visit as discussed in Section 5.14.5. A secondary endpoint for adherence is completion of one year of HP (patients who recur during the year of HP will be considered not evaluable for this secondary adherence endpoint), and recording of all adjuvant therapies administered (hormonal, chemotherapy, biologic or otherwise).

Patient questionnaires will assess reasons for off-protocol escalation for patients with pCR, or de-escalation for patients without pCR.

13.2.2 Secondary Endpoints

<u>Clinical secondary endpoints:</u>

1. pCR rate (defined as ypT0/is N0)
2. Residual Cancer Burden (RCB) scores[1]
3. All adjuvant therapies administered in all patients
4. EFS defined as the interval from study entry until invasive disease recurrence, new primary (breast or non-breast), or death due to any cause. Patients who are alive and event-free will be censored at the last known disease assessment.
5. RFI defined (for patients who undergo surgery for breast cancer) as the interval from the time of surgery until the occurrence of invasive local/regional recurrence, distant recurrence, or death due to breast cancer.
6. Overall survival defined as the interval from study entry until death due to any cause.

7. Exploratory endpoint: correlation between post-THP imaging findings and pathology findings in the surgical specimen

Correlative secondary endpoints:

1. Characterization of TIL phenotype and T cell repertoire before and after chemotherapy plus HER2-directed therapy.
2. Characterization of myeloid cells within the tumor microenvironment before and after standard THP administered neoadjuvantly to HER2+ breast cancer patients.
3. Characterization of the presence and functional activity of NK cells before and during treatment with standard chemotherapy + HER2-directed therapy or HER2-directed therapy alone, and correlation with response to anti-HER2 antibody therapy.

13.3 Sample Size and Analysis of Primary Endpoint

The primary assessment of feasibility will be conducted in the subgroup of patients who achieve pCR following THP using a Binomial exact test with a one-sided alpha = 0.05. If the true rate of adherence is 80% or lower in that subpopulation, de-escalation would not be deemed sufficiently feasible. The sample size was selected to have high power (>90%) to reject the null if the true rate of adherence were 95% or greater in the subset of patients who achieve pCR. Under this set of parameters, an exact Binomial test with n = 44 patients who achieve pCR will reject the null if 40 or more patients receive adjuvant HP-only therapy (exact alpha = 0.044). We expect a pCR rate of approximately 45%, based on the rate of 45.8% observed in a similar cohort of patients treated with neoadjuvant docetaxel/HP in the NeoSphere trial.[35] The following table provides decision rules for varying numbers of patients to achieve pCR.

Number to achieve pCR	Decision rule	Exact alpha	Exact beta
40	≥ 37	0.028	0.138
42	≥ 39	0.020	0.157
44	≥ 40	0.044	0.067
46	≥ 42	0.033	0.079
48	≥ 44	0.025	0.091
50	≥ 45	0.048	0.038

A total sample size of 100 patients is planned, in order to target n = 44 patients who achieve a pCR following THP. Patients who progress during neoadjuvant THP, withdraw consent to participate, receive neoadjuvant therapy in addition to THP, or fail to achieve pCR will not be included in the primary analysis. The analysis plan for these patients, who are not evaluable for the primary endpoint, is addressed in Section 13.5.

13.4 Interim Monitoring Plan

Total sample size will be re-estimated after 70% of patients are evaluable for response to neoadjuvant therapy. Based on the observed rate of unevaluable patients (as assessed based on

the first 70% of patients registered) due to exclusion criteria noted above, a revised target sample size will be based on the posterior mode from a beta-binomial model with the flat prior (a = 1, b = 1), setting a minimum permissible sample size of 100 patients and a maximum permissible sample size of 150 patients. This calculation for estimated maximum sample size is supported by the reported findings of the NeoSphere trial,[9] in which a similar neoadjuvant regimen was administered to a similar patient population. In NeoSphere, approximately 5% of patients were inevaluable for pCR due to miscellaneous reasons for protocol withdrawal prior to surgery, and the lower bound of the 95% confidence interval for pCR rate to neoadjuvant taxane/HP was 36.1%. No adjustments will be made to the primary analysis plan for the evaluable subpopulation who achieves pCR.

No formal early interim analysis for feasibility is planned, but the study can terminate early if more than 5 subjects elect to receive chemotherapy before 44 evaluable patients are reached.

We anticipate a low rate of recurrence during the time patients are receiving de-escalated adjuvant HP. Continuous monitoring of unacceptable levels of recurrence will use Pocock-style boundaries constructed following the method of Ivanova, et al (2005).[50] An event rate of 5% or less would be accepted, and the desired probability of stopping in the maximum planned sample size of 45 patients is 0.1, leading to the following stopping rules.

Number de-escalated	1	2 - 6	7 - 16	17 - 27	28 - 39	40 or more
Early recurrence	-	2	3	4	5	6

The probabilities of stopping the trial early are presented below:

True rate	Probability of stopping early
---	---
0.05	0.097
0.10	0.46
0.20	0.95
0.30	>0.99

13.5 Analysis of Secondary Endpoints

Descriptive statistics will be used to summarize patient and disease characteristics and for secondary endpoints related to adjuvant treatments received, adherence, and reported reasons for off-protocol treatment decisions.

The observed rates of pCR and RCB will be reported with exact binomial two-sided 90% confidence intervals. The distribution of the survival function and cumulative incidence function for EFS, RFI, and OS will be summarized using the Kaplan Meier product limit estimator and

90% confidence interval (CI) using Greenwood's formula for the standard error.

Clinical outcomes will be evaluated in the entire study population—including those patients who are evaluable for the primary endpoint and those who are not—and in the subgroups defined by HR+ and HR- breast cancer. Contrasts in the long-term outcomes (EFS, RFI, and OS) in patient subgroups according to endpoints at time of surgery will use landmark survival analyses to avoid guarantee time bias. Contrasts will be estimation-only and reported as ratios (odds ratios and hazard ratios) with two-sided 95% confidence intervals.

Analyses of correlative endpoints will be exploratory, and will use logistic regression and Cox proportional hazard models to evaluate the association between molecular phenotypes and clinical outcomes: binomial and time-to-event endpoints, respectively. For assessments at the time of surgery, landmark survival analyses will be performed. For post-baseline and serial assessments, including circulating tumor DNA, time-varying covariates will be used in survival analysis to infer the association.

14. PUBLICATION PLAN

The results should be made public within 3 years of reaching the end of the study. The end of the study is the time point at which the last data items for the primary endpoint are to be reported, or after the outcome data are sufficiently mature for analysis. If a report is planned to be published in a peer-reviewed journal, then that initial release may be an abstract. A full report of the outcomes should be made public no later than five (5) years after the end of the study.

15. REFERENCES

1. Symmans WF, Peintinger F, Hatzis C, et al: Measurement of residual breast cancer burden to predict survival after neoadjuvant chemotherapy. J Clin Oncol 25:4414-22, 2007

2. Slamon DJ, Clark GM, Wong SG, et al: Human breast cancer: correlation of relapse and survival with amplification of the HER-2/neu oncogene. Science 235:177-82, 1987

3. Slamon DJ, Leyland-Jones B, Shak S, et al: Use of chemotherapy plus a monoclonal antibody against HER2 for metastatic breast cancer that overexpresses HER2. N Engl J Med 344:783-92, 2001

4. Romond EH, Perez EA, Bryant J, et al: Trastuzumab plus adjuvant chemotherapy for operable HER2-positive breast cancer. N Engl J Med 353:1673-84, 2005

5. Piccart-Gebhart MJ, Procter M, Leyland-Jones B, et al: Trastuzumab after adjuvant chemotherapy in HER2-positive breast cancer. N Engl J Med 353:1659-72, 2005

6. Slamon D, Eiermann W, Robert N, et al: Adjuvant trastuzumab in HER2-positive breast cancer. N Engl J Med 365:1273-83, 2011

7. Joensuu H, Kellokumpu-Lehtinen PL, Bono P, et al: Adjuvant docetaxel or vinorelbine with or without trastuzumab for breast cancer. N Engl J Med 354:809-20, 2006

8. Buzdar AU, Valero V, Ibrahim NK, et al: Neoadjuvant therapy with paclitaxel followed by 5-fluorouracil, epirubicin, and cyclophosphamide chemotherapy and concurrent trastuzumab in human epidermal growth factor receptor 2-positive operable breast cancer: an update of the initial randomized study population and data of additional patients treated with the same regimen. Clin Cancer Res 13:228-33, 2007

9. Gianni L, Eiermann W, Semiglazov V, et al: Neoadjuvant chemotherapy with trastuzumab followed by adjuvant trastuzumab versus neoadjuvant chemotherapy alone, in patients with HER2-positive locally advanced breast cancer (the NOAH trial): a randomised controlled superiority trial with a parallel HER2-negative cohort. Lancet 375:377-84, 2010

10. Untch M, Rezai M, Loibl S, et al: Neoadjuvant treatment with trastuzumab in HER2-positive breast cancer: results from the GeparQuattro study. J Clin Oncol 28:2024-31, 2010

11. Baselga J, Bradbury I, Eidtmann H, et al: First Results of the NeoALTTO Trial (BIG 01-06 / EGF 106903): A Phase III, Randomized, Open Label, Neoadjuvant Study of Lapatinib, Trastuzumab, and Their Combination Plus Paclitaxel in Women with HER2-Positive Primary Breast Cancer. Cancer Res 70(24 Suppl.):Abstract S3-3, 2010

12. Gianni L, Pienkowski T, Im Y-H, et al: Neoadjuvant Pertuzumab (P) and Trastuzumab (H): Antitumor and Safety Analysis of a Randomized Phase II Study ('NeoSphere'). Cancer Res 70(24 Suppl.):Abstract S3-2, 2010

13. Untch M, Loibl S, Bischoff J, et al: Lapatinib vs Trastuzumab in Combination with Neoadjuvant Anthracycline-Taxane-Based Chemotherapy: Primary Efficacy Endpoint Analysis of the GEPARQUINTO STUDY (GBG 44). Cancer Res 70(24 Suppl.) Abstract S3-1, 2010

14. Baselga J, et al.: First Results of the NeoALTTO Trial (BIG 01-06 / EGF 106903): A Phase III, Randomized, Open Label, Neoadjuvant Study of Lapatinib, Trastuzumab, and Their Combination Plus Paclitaxel in Women with HER2-Positive Primary Breast Cancer. Cancer Res 70(24 Suppl.), 2010: p. Abstract S3-3

15. Gianni L, et al.: Neoadjuvant Pertuzumab (P) and Trastuzumab (H): Antitumor and Safety Analysis of a Randomized Phase II Study ('NeoSphere'). Cancer Res 70(24 Suppl.), Abstract S3-2, 2010

16. Robidoux A, Tang G, Rastogi P, et al: Evaluation of lapatinib as a component of neoadjuvant therapy for HER2+ operable breast cancer: NSABP protocol B-41. ASCO Meeting Abstracts 30:LBA506, 2012

17. Carey LA, Berry DA, Ollila D, et al: Clinical and translational results of CALGB 40601: A neoadjuvant phase III trial of weekly paclitaxel and trastuzumab with or without lapatinib for HER2-positive breast cancer. ASCO Meeting Abstracts 31:500, 2013

18. Dowsett M, Nielsen TO, A'Hern R, et al: Assessment of Ki67 in breast cancer: recommendations from the International Ki67 in Breast Cancer working group. J Natl Cancer Inst 103:1656-64, 2011

19. Baselga J, Cortes J, Kim SB, et al: Pertuzumab plus trastuzumab plus docetaxel for metastatic breast cancer. N Engl J Med 366:109-19, 2011

20. Gianni L, Pienkowski T, Im YH, et al: Efficacy and safety of neoadjuvant pertuzumab and trastuzumab in women with locally advanced, inflammatory, or early HER2-positive breast cancer (NeoSphere): a randomised multicentre, open-label, phase 2 trial. Lancet Oncol 13:25-32, 2011

21. Schneeweiss A, Chia S, Hickish T, et al: Neoadjuvant Pertuzumab and Trastuzumab Concurrent or Sequential with an Anthracycline-Containing or Concurrent with an Anthracycline-Free Standard Regimen: A Randomized Phase II Study (TRYPHAENA). Cancer Res 71(24 Suppl):S5-6, 2011

22. von Minckwitz G, Procter M, de Azambuja E, et al: Adjuvant Pertuzumab and Trastuzumab in Early HER2-Positive Breast Cancer. N Engl J Med 377:122-131, 2017

23. Portera CC, Walshe JM, Rosing DR, et al: Cardiac toxicity and efficacy of trastuzumab combined with pertuzumab in patients with [corrected] human epidermal growth factor receptor 2-positive metastatic breast cancer. Clin Cancer Res 14:2710-6, 2008

24. Baselga J, Gelmon KA, Verma S, et al: Phase II Trial of Pertuzumab and Trastuzumab in Patients With Human Epidermal Growth Factor Receptor 2-Positive Metastatic Breast Cancer That Progressed During Prior Trastuzumab Therapy. J Clin Oncol, 2010 28:1138-1144, 2010

25. Cortes J, Fumoleau P, Bianchi GV, et al: Pertuzumab monotherapy after trastuzumab-based treatment and subsequent reintroduction of trastuzumab: activity and tolerability in patients with advanced human epidermal growth factor receptor 2-positive breast cancer. J Clin Oncol 30:1594-600, 2012

26. Baselga J, Cortes J, Kim SB, et al: Pertuzumab plus trastuzumab plus docetaxel for metastatic breast cancer. N Engl J Med 366:109-19, 2012

27. Swain SM, Kim S-B, Cortés J, et al: Pertuzumab, trastuzumab, and docetaxel for HER2-positive metastatic breast cancer (CLEOPATRA study): overall survival results from a randomised, double-blind, placebo-controlled, phase 3 study. The Lancet Oncology, 2013

28. Miles D, J. B, Amadori D, et al: Pertuzumab (P) in Combination with Trastuzumab (T) and Docetaxel (D) in Elderly Patients with HER2-Positive Metastatic Breast Cancer in the CLEOPATRA Study. Cancer Res 72 (Suppl.): (3) P5-18-01, 2012

29. Cortes J, Baselga J, Im Y-H, et al: Quality of life assessment in CLEOPATRA, a phase III study combining pertuzumab with trastuzumab and docetaxel in

metastatic breast cancer. J Clin Oncol 30:598, 2012

30. Baselga J, Cortes J, Im S-A, et al: Biomarker analyses in CLEOPATRA: A phase III, placebo-controlled study of pertuzumab in HER2-positive, first-line metastatic breast cancer (MBC). Cancer Res 72 (24) Suppl: 3 Abstract S5-1, 2012

31. NCCN Clinical Practice Guidelines in Oncology - Breast Cancer, Version 2.2017. National Comprehensive Cancer Network, 2017

32. von Minckwitz G, Untch M, Blohmer JU, et al: Definition and impact of pathologic complete response on prognosis after neoadjuvant chemotherapy in various intrinsic breast cancer subtypes. J Clin Oncol 30:1796-804, 2012

33. Gianni L, Pienkowski T, Im YH, et al: 5-year analysis of neoadjuvant pertuzumab and trastuzumab in patients with locally advanced, inflammatory, or early-stage HER2-positive breast cancer (NeoSphere): a multicentre, open-label, phase 2 randomised trial. Lancet Oncol 17:791-800, 2016

34. Cortazar P, Zhang L, Untch M, et al: Pathological complete response and long-term clinical benefit in breast cancer: the CTNeoBC pooled analysis. Lancet 384:164-72, 2014

35. Gianni L, Pienkowski T, Im YH, et al: Efficacy and safety of neoadjuvant pertuzumab and trastuzumab in women with locally advanced, inflammatory, or early HER2-positive breast cancer (NeoSphere): a randomised multicentre, open-label, phase 2 trial. Lancet Oncol 13:25-32, 2012

36. Swain SM, Baselga J, Kim SB, et al: Pertuzumab, trastuzumab, and docetaxel in HER2-positive metastatic breast cancer. N Engl J Med 372:724-34, 2015

37. Tolaney SM, Barry WT, Dang CT, et al: Adjuvant paclitaxel and trastuzumab for node-negative, HER2-positive breast cancer. N Engl J Med 372:134-41, 2015

38. Smyth LM, Iyengar NM, Chen MF, et al: Weekly paclitaxel with trastuzumab and pertuzumab in patients with HER2-overexpressing metastatic breast cancer: overall survival and updated progression-free survival results from a phase II study. Breast Cancer Res Treat 158:91-7, 2016

39. Denkert C, von Minckwitz G, Darb-Esfahani S, et al: Tumour-infiltrating lymphocytes and prognosis in different subtypes of breast cancer: a pooled analysis of 3771 patients treated with neoadjuvant therapy. Lancet Oncol 19:40-50, 2018

40. Salgado R, Denkert C, Demaria S, et al: The evaluation of tumor-infiltrating lymphocytes (TILs) in breast cancer: recommendations by an International TILs Working Group 2014. Ann Oncol 26:259-71, 2015

41. Spector NL, Blackwell KL: Understanding the mechanisms behind trastuzumab therapy for human epidermal growth factor receptor 2-positive breast cancer. J Clin Oncol 27:5838-47, 2009

42. Clynes RA, Towers TL, Presta LG, et al: Inhibitory Fc receptors modulate in vivo cytotoxicity against tumor targets. Nat Med 6:443-6, 2000

43. Spiridon CI, Guinn S, Vitetta ES: A comparison of the in vitro and in vivo activities of IgG and F(ab')2 fragments of a mixture of three monoclonal anti-Her-2 antibodies. Clin Cancer Res 10:3542-51, 2004

44. Scheuer W, Friess T, Burtscher H, et al: Strongly enhanced antitumor activity of trastuzumab and pertuzumab combination treatment on HER2-positive human xenograft tumor models. Cancer Res 69:9330-6, 2009

45. Crawford J, Caserta C, Roila F, et al: Hematopoietic growth factors:

ESMO Clinical Practice Guidelines for the applications. Ann Oncol 21 Suppl 5:v248-51, 2010

46. Crawford J, Armitage J, Balducci L, et al: Myeloid growth factors. J Natl Compr Canc Netw 11:1266-90, 2013

47. Schneeweiss A, Chia S, Hickish T, et al: Pertuzumab plus trastuzumab in combination with standard neoadjuvant anthracycline-containing and anthracycline-free chemotherapy regimens in patients with HER2-positive early breast cancer: a randomized phase II cardiac safety study (TRYPHAENA). Ann Oncol 24:2278-84, 2013

48. Esserman LE, Cura MA, DaCosta D: Recognizing pitfalls in early and late migration of clip markers after imaging-guided directional vacuum-assisted biopsy. Radiographics 24:147-56, 2004

49. Loi S, Symmans WF, Bartlett JM, et al: Proposals for uniform collection of biospecimens from neoadjuvant breast cancer clinical trials: timing and specimen types. Lancet Oncol 12:1162-8, 2011

50. Ivanova A, Qaqish BF, Schell MJ: Continuous toxicity monitoring in phase II trials in oncology. Biometrics 61:540-5, 2005

16. APPENDIX A: PERFORMANCE STATUS CRITERIA

<table>
<tr><th colspan="2">ECOG Performance Status Scale</th><th colspan="2">Karnofsky Performance Scale</th></tr>
<tr><td>Grade</td><td>Descriptions</td><td>Percent</td><td>Description</td></tr>
<tr><td rowspan="2">0</td><td rowspan="2">Normal activity. Fully active, able to carry on all pre-disease performance without restriction.</td><td>100</td><td>Normal, no complaints, no evidence of disease.</td></tr>
<tr><td>90</td><td>Able to carry on normal activity; minor signs or symptoms of disease.</td></tr>
<tr><td rowspan="2">1</td><td rowspan="2">Symptoms, but ambulatory. Restricted in physically strenuous activity, but ambulatory and able to carry out work of a light or sedentary nature (e.g., light housework, office work).</td><td>80</td><td>Normal activity with effort; some signs or symptoms of disease.</td></tr>
<tr><td>70</td><td>Cares for self, unable to carry on normal activity or to do active work.</td></tr>
<tr><td rowspan="2">2</td><td rowspan="2">In bed <50% of the time. Ambulatory and capable of all self-care, but unable to carry out any work activities. Up and about more than 50% of waking hours.</td><td>60</td><td>Requires occasional assistance, but is able to care for most of his/her needs.</td></tr>
<tr><td>50</td><td>Requires considerable assistance and frequent medical care.</td></tr>
<tr><td rowspan="2">3</td><td rowspan="2">In bed >50% of the time. Capable of only limited self-care, confined to bed or chair more than 50% of waking hours.</td><td>40</td><td>Disabled, requires special care and assistance.</td></tr>
<tr><td>30</td><td>Severely disabled, hospitalization indicated. Death not imminent.</td></tr>
<tr><td rowspan="2">4</td><td rowspan="2">100% bedridden. Completely disabled. Cannot carry on any self-care. Totally confined to bed or chair.</td><td>20</td><td>Very sick, hospitalization indicated. Death not imminent.</td></tr>
<tr><td>10</td><td>Moribund, fatal processes progressing rapidly.</td></tr>
<tr><td>5</td><td>Dead.</td><td>0</td><td>Dead.</td></tr>
</table>

17. APPENDIX B: NEW YORK HEART ASSOCIATION (NYHA) CLASSIFICATIONS

The New York Heart Association (NYHA) Cardiac Disease Classification provides a functional and therapeutic classification for the prescription of physical activity for cardiac participants. Based on NYHA definitions, participants are to be classified as follows:

Class	Definition
Class I	Subjects with no limitation of activities; they suffer no symptoms from ordinary activities.
Class II	Subjects with slight, mild limitation of activity; they are comfortable with rest or with mild exertion.
Class III	Subjects with marked limitation of activity; they are comfortable only at rest.
Class IV	Subjects who should be at complete rest, confined to bed or chair; any physical activity brings on discomfort and symptoms occur at rest.

18. APPENDIX C: DF/HCC MULTI-CENTER DATA AND SAFETY MONITORING PLAN

DFCI IRB Protocol #: 18-394

TABLE OF CONTENTS

1. INTRODUCTION

1.1 Purpose
1.2 Multi-Center Data and Safety Monitoring Plan Definitions

2. GENERAL ROLES AND RESPONSIBILITIES

2.1 DF/HCC Sponsor
2.2 Coordinating Center
2.3 Participating Institution

3. DF/HCC REQUIREMENTS FOR MULTI-CENTER PROTOCOLS

3.1 Protocol Distribution
3.2 Protocol Revisions and Closures
3.3 Informed Consent Requirements
3.4 IRB Documentation
3.5 IRB Re-Approval
3.6 Participant Confidentiality and Authorization Statement
3.7 DF/HCC Multi-Center Protocol Registration Policy
3.8 DF/HCC Protocol Case Number
3.9 Safety Assessments and Toxicity Monitoring
3.10 Data Management

4. REQUISITIONING INVESTIGATIONAL DRUG

5. MONITORING: QUALITY CONTROL

5.1 Ongoing Monitoring of Protocol Compliance
5.2 Monitoring Reports
5.3 Accrual Monitoring

6. AUDITING: QUALITY ASSURANCE

6.1 DF/HCC Internal Audits
6.2 Audit Notifications
6.3 Audit Reports
6.4 Participating Institution Performance

1. INTRODUCTION

The Dana-Farber/Harvard Cancer Center Multi-Center Data and Safety Monitoring Plan (DF/HCC DSMP) outlines the procedures for conducting a DF/HCC Multi-Center research protocol. The DF/HCC DSMP serves as a reference for any sites external to DF/HCC that are participating in a DF/HCC clinical trial.

1.1 Purpose

To establish standards that will ensure that a Dana-Farber/Harvard Cancer Center Multi-Center protocol will comply with Federal Regulations, Health Insurance Portability and Accountability Act (HIPAA) requirements and applicable DF/HCC Standard Operating Procedures.

1.2 Multi-Center Data and Safety Monitoring Plan Definitions

DF/HCC Multi-Center Protocol: A research protocol in which one or more outside institutions are collaborating with Dana-Farber/Harvard Cancer Center where a DF/HCC investigator is the sponsor. DF/HCC includes Dana-Farber/Partners Cancer Care (DF/PCC) Network Clinical Trial Affiliates.

Lead Institution: One of the Dana-Farber/Harvard Cancer Center consortium members (Dana-Farber Cancer Institute (DFCI), Massachusetts General Hospital (MGH), Beth Israel Deaconess Medical Center (BIDMC), Boston Children's Hospital (BCH), Brigham and Women's Hospital (BWH) responsible for the coordination, development, submission, and approval of a protocol as well as its subsequent amendments per the DFCI IRB and applicable regulatory guidelines (CTEP, Food and Drug Administration (FDA), Office of Biotechnology Activities (OBA) etc.). The *Lead Institution is typically the home of the DF/HCC Sponsor. The Lead Institution also typically serves as the Coordinating Center for the DF/HCC Multi-Center Protocol.*

DF/HCC Sponsor: The person sponsoring the submitted Multi-Center protocol who takes responsibility for initiation, management and conduct of the protocol at all research locations. In applicable protocols, the DF/HCC Sponsor will serve as the single liaison with any regulatory agencies (i.e. FDA*)*. The DF/HCC Sponsor has ultimate authority over the protocol and is responsible for the conduct of the study at DF/HCC and all Participating Institutions. In *most cases the DF/HCC Sponsor is the same person as the DF/HCC Overall Principal Investigator; however, both roles can be filled by two different people.*

Participating Institution: An institution that is outside the DF/HCC and DF/PCC consortium that is collaborating with DF/HCC on a protocol where the sponsor is a DF/HCC Investigator. The Participating Institution acknowledges the DF/HCC Sponsor as having the ultimate authority and responsibility for the overall conduct of the study.

Coordinating Center: The entity (i.e. Lead Institution, Medical Monitor, Contract Research Organization (CRO), etc) that provides administrative support to the DF/HCC Sponsor in order that he/she may fulfill the responsibilities outlined in the protocol document and DSMP, and as specified in applicable regulatory guidelines (i.e. CTEP Multi-Center Guidelines). In general, the

Lead Institution is the Coordinating Center for the DF/HCC Multi-Center Protocol.

DF/HCC Office of Data Quality (ODQ): A group within DF/HCC responsible ensuring high-quality standards are used for data collection and the ongoing management of clinical trials, auditing, and data and safety monitoring. ODQ also coordinates quality assurance efforts related to multi-center clinical research.

DF/HCC Clinical Trials Research Informatics Office (CTRIO): A group within DF/HCC responsible for providing a comprehensive data management platform for managing clinical trial data.

2 GENERAL ROLES AND RESPONSIBILITIES

For DF/HCC Multi-Center Protocols, the DF/HCC Sponsor, the Coordinating Center, and the Participating Institutions are expected to adhere to the following general responsibilities:

2.1 DF/HCC Sponsor

The DF/HCC Sponsor, Adrienne Gropper Waks, MD will accept responsibility for all aspects of conducting a DF/HCC Multi-Center protocol which includes but is not limited to:

- Oversee the coordination, development, submission, and approval of the protocol as well as subsequent amendments.
- Ensure that the investigators, study team members, and Participating Institutions are qualified and appropriately resourced to conduct the protocol.
- Include the Multi-Center Data and Safety Monitoring Plan as an appendix to the protocol.
- Ensure all Participating Institutions are using the correct version of the protocol.
- Ensure that each participating investigator and study team member receives adequate protocol training (and/or a Site Initiation Visit prior to enrolling participants) and throughout trial’s conduct as needed.
- Ensure the protocol will be provided to each participating site in a language understandable to all applicable site personnel when English is not the primary language.
- Monitor progress and overall conduct of the study at all Participating Institutions.
- Ensure all DFCI Institutional Review Board (IRB), DF/HCC and other applicable (i.e. FDA) reporting requirements are met.
- Review data and maintain timely submission of data for study analysis.
- Act as the single liaison with FDA (investigator-held IND trials) as applicable.
- Ensure compliance with all requirements as set forth in the Code of Federal Regulations, applicable DF/HCC requirements, HIPAA requirements, and the approved protocol.
- Commit to the provision that the protocol will not be rewritten or modified by anyone other than the DF/HCC Sponsor.
- Identify and qualify Participating Institutions and obtain accrual commitments prior to extending the protocol to that site.
- Monitor accrual and address Participating Institutions that are not meeting their accrual requirements.

2.2 Coordinating Center

The general responsibilities of the Coordinating Center may include but are not limited to:

- Assist in protocol development.
- Maintain FDA correspondence as applicable.
- Review registration materials for eligibility and register participants from Participating Institutions in the DF/HCC clinical trial management system (CTMS).
- Distribute protocol and informed consent document updates to Participating Institutions as needed.
- Oversee the data collection process from Participating Institutions.
- Maintain documentation of Serious Adverse Event (SAE) reports and deviations/violation submitted by Participating Institutions and provide to the DF/HCC Sponsor for timely review and submission to the DFCI IRB, as necessary.
- Distribute serious adverse events reported to the DF/HCC Sponsor that fall under the DFCI IRB Adverse Event Reporting Policy to all Participating Institutions.
- Provide Participating Institutions with information regarding DF/HCC requirements that they will be expected to comply with.
- Carry out plan to monitor Participating Institutions either by on-site or remote monitoring.
- Maintain Regulatory documents of all Participating Institutions which includes but is not limited to the following: local IRB approvals/notifications from all Participating Institutions, confirmation of Federalwide Assurances (FWAs) for all sites, all SAE submissions, Screening Logs for all sites, IRB approved consents for all sites
- Conduct regular communications with all Participating Institutions (conference calls, emails, etc) and maintain documentation all relevant communications.

2.3 Participating Institution

Each Participating Institution is expected to comply with all applicable federal regulations and DF/HCC requirements, the protocol and HIPAA requirements.

The general responsibilities for each Participating Institution may include but are not limited to:

- Document the delegation of research specific activities to study personnel.
- Commit to the accrual of participants to the protocol.
- Submit protocol and/or amendments to their local IRB.
- Maintain regulatory files as per sponsor requirements.
- Provide the Coordinating Center with regulatory documents or source documents as requested.
- Participate in protocol training prior to enrolling participants and throughout the trial as required (i.e. teleconferences).
- Update Coordinating Center with research staff changes on a timely basis.
- Register participants through the Coordinating Center prior to beginning research related activities.
- Submit Serious Adverse Event (SAE) reports to local IRB per institutional requirements and to the Coordinating Center, in accordance with DF/HCC requirements.
- Submit protocol deviations and violations to local IRB per institutional requirements and

to the DF/HCC Sponsor in accordance with DF/HCC requirements.

- Order, store and dispense investigational agents and/or other protocol mandated drugs per federal guidelines and protocol requirements.
- Have office space, office equipment, and internet access that meet HIPAA standards.
- Participate in any quality assurance activities and meet with monitors or auditors at the conclusion of a visit to review findings.
- Promptly provide follow-up and/or corrective action plans for any monitoring queries or audit findings.

3 DF/HCC REQUIREMENTS FOR MULTI-CENTER PROTOCOLS

The following section will clarify DF/HCC Requirements and further detail the expectations for participating in a DF/HCC Multi-Center protocol.

3.1 Protocol Distribution

The Coordinating Center will distribute the final DFCI IRB approved protocol and any subsequent amended protocols to all Participating Institutions.

3.2 Protocol Revisions and Closures

The Participating Institutions will receive notification of protocol revisions and closures from the Coordinating Center. It is the individual Participating Institution's responsibility to notify its IRB of these revisions.

- **Non life-threatening revisions:** Participating Institutions will receive written notification of protocol revisions regarding non life-threatening events from the Coordinating Center. Non-life-threatening protocol revisions must be IRB approved and implemented within 90 days from receipt of the notification.

- **Revisions for life-threatening causes:** Participating Institutions will receive immediate notification from the Coordinating Center concerning protocol revisions required to protect lives with follow-up by fax, mail, e-mail, etc. Life-threatening protocol revisions will be implemented immediately followed by IRB request for approval.

- **Protocol closures and temporary holds:** Participating Institutions will receive notification of protocol closures and temporary holds from the Coordinating Center. Closures and holds will be effective immediately. In addition, the Coordinating Center, will update the Participating Institutions on an ongoing basis about protocol accrual data so that they will be aware of imminent protocol closures.

3.3 Informed Consent Requirements

The DF/HCC approved informed consent document will serve as a template for the informed

consent for Participating Institutions. The Participating Institution consent form must follow the consent template as closely as possible and should adhere to specifications outlined in the DF/HCC Guidance Document on Model Consent Language for PI-Initiated Multi-Center Protocols. This document will be provided separately to each Participating Institution upon request.

Participating Institutions are to send their version of the informed consent document and HIPAA authorization, if a separate document, to the Coordinating Center for review and approval prior to submission to their local IRB. The approved consent form must also be submitted to the Coordinating Center after approval by the local IRB for all consent versions.

The Principal Investigator (PI) at each Participating Institution will identify the physician members of the study team who will be obtaining consent and signing the consent form for therapeutic protocols. Participating institutions must follow the DF/HCC requirement that for all interventional drug, biologic, or device research, only attending physicians may obtain initial informed consent and any re-consent that requires a full revised consent form.

3.4 IRB Documentation

The following must be on file with the Coordinating Center:

- Initial approval letter of the Participating Institution's IRB.
- Copy of the Informed Consent Form(s) approved by the Participating Institution’s IRB.
- Participating Institution’s IRB approval for all amendments.
- Annual approval letters by the Participating Institution's IRB.

3.5 IRB Re-Approval

Verification of IRB re-approval from the Participating Institutions is required in order to continue research activities. There is no grace period for continuing approvals.

The Coordinating Center will not register participants if a re-approval letter is not received from the Participating Institution on or before the anniversary of the previous approval date.

3.6 Participant Confidentiality and Authorization Statement

In 1996, congress passed the first federal law covering the privacy of health information known as the Health Insurance Portability and Accountability Act (HIPPA). Any information, related to the physical or mental health of an individual is called Protected Health Information (PHI). HIPAA outlines how and under what circumstances PHI can be used or disclosed.

In order for covered entities to use or disclose protected health information during the course of a study, the study participant must sign an authorization statement. This authorization statement may or may not be separate from the informed consent document. The Coordinating Center, with the approval from the DFCI IRB will provide a consent template, with information regarding authorization for the disclosure of protected health information.

The DF/HCC Sponsor will use all efforts to limit its use of protected health information in its trials. However, because of the nature of these trials, certain protected health information must be collected. DF/HCC has chosen to use authorizations, signed by the participant in the trial, rather than limited data sets with data use agreements.

3.6.1 DF/HCC Multi-Center Protocol Confidentiality

All documents, investigative reports, or information relating to the participant are strictly confidential. Whenever reasonably feasible, any participant specific reports (i.e. Pathology Reports, MRI Reports, Operative Reports, etc.) submitted to the Coordinating Center should be de-identified. It is recommended that the assigned protocol case number (as described below) be used for all participant specific documents. Participant initials may be included or retained for cross verification of identification.

3.7 DF/HCC Multi-Center Protocol Registration Policy

3.7.1 Participant Registration and Randomization

Please refer to Protocol Section 4.3 and 4.4 for participant registration information. Treatment cannot begin until site has received confirmation that participant has been registered with DF/HCC CTMS.

3.7.2 Initiation of Therapy

Participants must be registered with the DF/HCC CTMS before the initiation of treatment or other protocol-specific interventions. Treatment and other protocol-specific interventions may not be initiated until the Participating Institution receives confirmation of the participant's registration from the Coordinating Center. The DF/HCC Sponsor and DFCI IRB must be notified of any violations to this policy.

3.7.3 Eligibility Exceptions

No exceptions to the eligibility requirements for a protocol without DFCI IRB approval will be permitted. All Participating Institutions are required to fully comply with this requirement. The process for requesting an eligibility exception is defined below.

3.8 DF/HCC Protocol Case Number

At the time of registration, the following identifiers are required for all subjects: initials, date of birth, gender, race and ethnicity. Once eligibility has been established and the participant successfully registered, the participant is assigned a unique protocol case number. Participating Institutions should submit all de-identified subsequent communication and documents to the Coordinating Center, using this case number to identify the subject.

3.8.1 Protocol Deviations, Exceptions and Violations

Federal Regulations require an IRB to review proposed changes in a research activity to ensure that researchers do not initiate changes in approved research without IRB review and approval, except when necessary to eliminate apparent immediate hazards to the participant. DF/HCC requires all departures from the defined procedures set forth in the IRB approved protocol to be reported to the DF/HCC Sponsor, who in turn is responsible for reporting to the DFCI IRB.

For reporting purposes, DF/HCC uses the terms "violation", "deviation" and "exception" to describe departures from a protocol. All Participating Institutions must adhere to these requirements for reporting to the DF/HCC Sponsor and will follow their institutional policy for reporting to their local IRB.

3.8.2 Definitions

Protocol Deviation: Any departure from the defined procedures set forth in the IRB-approved protocol which is *prospectively approved* prior to its implementation.

Protocol Exception: Any protocol deviation that relates to the eligibility criteria, e.g. enrollment of a participant who does not meet all inclusion/exclusion criteria.

Protocol Violation: Any protocol departure that was not *prospectively approved* by the IRB prior to its initiation or implementation.

3.8.3 Reporting Procedures

DF/HCC Sponsor: is responsible for ensuring that clear documentation is available in the medical record and/or regulatory documents to describe all protocol exceptions, deviations and violations. The DF/HCC Sponsor will also be responsible for ensuring that all protocol violations/deviations are promptly reported per DFCI IRB guidelines.

Participating Institutions: Protocol deviations require prospective approval from the DFCI IRB. The Participating Institution must submit the deviation request to the Coordinating Center who will then submit the deviation request to the DFCI IRB. Upon DFCI IRB approval the deviation is submitted to the Participating Institution IRB, per institutional policy. A copy of the Participating Institution's IRB report and determination will be forwarded to the Coordinating Center within 10 business days after the original submission. The deviation may not be implemented without all required approvals.

All protocol violations must be sent to the Coordinating Center in a timely manner. The Coordinating Center will provide training for the requirements for the reporting of violations.

Coordinating Center: Upon receipt of the violation/deviation report from the Participating Institution, the Coordinating Center will submit the report to the DF/HCC Sponsor for review. Subsequently, the Participating Institution's IRB

violation/deviation report will be submitted to the DFCI IRB for review per DFCI IRB reporting guidelines.

3.9 Safety Assessments and Toxicity Monitoring

The study teams at all participating institutions are responsible for protecting the safety, rights and well-being of study participants. Recording and reporting of adverse events that occur during the course of a study help ensure the continuing safety of study participants.

All participants receiving study agents and/or other protocol mandated therapy will be evaluated for safety. The safety parameters include all laboratory tests and hematological abnormalities, physical examination findings, and spontaneous reports of adverse events reported by participants. All toxicities encountered during the study will be evaluated according to the NCI criteria specified in the protocol. Life-threatening toxicities must be reported immediately to the DF/HCC Sponsor via the Coordinating Center.

Additional safety assessments and toxicity monitoring will be outlined in the protocol.

3.9.1 Guidelines for Reporting Serious Adverse Events

Guidelines for reporting Adverse Events (AEs) and Serious Adverse Events (SAEs) are detailed in protocol section 7.

Participating Institutions must report the SAEs to the DF/HCC Sponsor and the Coordinating Center following the DFCI IRB Adverse Event Reporting Policy.

The Coordinating Center will maintain documentation of all Participating Institution Adverse Event reports and be responsible for communicating to all participating investigators, any observations reportable under the DFCI IRB Reporting Requirements. Participating Institutions will review and submit to their IRB according to their institutional policies and procedures.

3.10 Data Management

DF/HCC CTRIO develops case report forms (CRF/eCRFs), for use with the protocol. These forms are designed to collect data for each study. DF/HCC CTRIO provides a web based training for all eCRF users.

3.10.1 Data Forms Review

Data submissions are monitored for timeliness and completeness of submission. If study forms are received with missing or questionable data, the submitting institution will receive a written or electronic query from the DF/HCC Office of Data Quality, Coordinating Center, or designee.

Responses to all queries should be completed and submitted within 14 calendar days.

Responses may be returned on the written query or on an amended paper case report form, or in the case of electronic queries, within the electronic data capture (eDC) system. In the case of a written query for data submitted on a paper case report form, the query must be attached to the specific data being re-submitted in response.

If study forms are not submitted on schedule, the Participating Institution will periodically receive a Missing Form Report from the Coordinating Center noting the missing forms.

4 REQUISITIONING INVESTIGATIONAL DRUG

The ordering of the study agents is specified in the protocol section 8.

Participating Institutions should order their own agent regardless of the supplier.

If the agent is commercially available, check with the local Director of Pharmacy and/or the Research Pharmacy to ensure that the agent is in stock. If the agent is not stocked, ensure that the agent can be ordered once the protocol is approved by the local IRB.

If the agent is investigational, ensure that the pharmacy will be able to receive and store the agent according to state and federal requirements. The local IRB should be kept informed of who will supply the agent (i.e., NCI or a pharmaceutical company) so that any regulatory responsibilities can be met in a timely fashion.

5 MONITORING: QUALITY CONTROL

The quality control process for a clinical trial requires verification of protocol compliance and data accuracy. The Coordinating Center, with the aid of the DF/HCC Office of Data Quality, provides quality control oversight for the protocol.

5.1 Ongoing Monitoring of Protocol Compliance

The Participating Institutions may be required to submit participant source documents to the Coordinating Center for monitoring. Participating Institution may also be subject to on-site monitoring conducted by the Coordinating Center.

The Coordinating Center will implement ongoing monitoring activities to ensure that Participating Institutions are complying with regulatory and protocol requirements, data quality, and participant safety. Monitoring practices may include but are not limited to source data verification, and review and analysis of eligibility requirements, informed consent procedures, adverse events and all associated documentation, review of study drug administration/treatment, regulatory files, protocol departures reporting, pharmacy records, response assessments, and data management.

Additionally, a plan will be formulated to provide regular and ongoing communication to Participating Institutions about study related information which will include participation in regular Lead Institution initiated teleconferences. Teleconferences will occur every 2 weeks and will continue regularly until completion of accrual. Upon completion of accrual, teleconferences will occur monthly until all patients complete protocol therapy. Upon completion of protocol therapy, teleconferences will occur every 3 months until study completion. Additional communication may be distributed via “Newsletter” or email as deemed appropriate by DF/HCC Sponsor.

On-Site Monitoring: On-site monitoring will occur on an as-needed basis. Participating Institutions will be required to provide access to participants’ complete medical record and source documents for source documentation verification during the visit. In addition, Participating Institutions should provide access to regulatory documents, pharmacy records, local policies related to the conduct of research, and any other trial-related documentation maintained by the Participating Site. On-site monitoring visits can be substituted with remote (virtual) monitoring visits at the discretion of the Principal Investigator.

Remote Monitoring: Remote monitoring will be performed on an as-needed basis by the Clinical Trial Monitor. Sites will be asked to provide source documentation via fax, email, or mail as specified by the Clinical Trial Monitor for virtual monitoring.

5.2 Monitoring Reports

The DF/HCC Sponsor will review all monitoring reports to ensure protocol compliance. The DF/HCC Sponsor may increase the monitoring activities at Participating Institutions that are unable to comply with the protocol, DF/HCC Sponsor requirements or federal and local regulations.

5.3 Accrual Monitoring

Prior to extending a protocol to an external site, the DF/HCC Sponsor will establish accrual requirements for each participating institution. Accrual will be monitored for each participating institution by the DF/HCC Sponsor or designee. Sites that are not meeting their accrual expectations may be subject to termination. Sites are expected to accrue at least 3 patients per year.

6 AUDITING: QUALITY ASSURANCE

Auditing is a method of Quality Assurance and involves the systematic and independent examination of all trial related activities and documents. Audits determine if evaluated activities were appropriately conducted and whether data was generated, recorded and analyzed, and accurately reported per the protocol, applicable Policies, and the Code of Federal Regulations (CFR).

6.1 DF/HCC Internal Audits

All Participating Institutions are subject to audit by the DF/HCC Office of Data Quality (ODQ). Typically, approximately 3-4 participants would be audited at the site over a 2-day period. If violations which impact participant safety or the integrity of the study are found, more participant records may be audited.

6.2 Audit Notifications

It is the Participating Institution's responsibility to notify the Coordinating Center of all external audits or inspections (e.g., FDA, EMA, NCI) that involve this protocol. All institutions will forward a copy of final audit and/or re-audit reports and corrective action plans (if applicable) to the Coordinating Center, within 12 weeks after the audit date.

6.3 Audit Reports

The DF/HCC Sponsor will review all final audit reports and corrective action plans, if applicable. The Coordinating Center, must forward any reports to the DF/HCC ODQ per DF/HCC policy for review by the DF/HCC Audit Committee. For unacceptable audits, the DF/HCC Audit Committee would forward the final audit report and corrective action plan to the DFCI IRB as applicable.

6.4 Participating Institution Performance

The DF/HCC Sponsor and the DFCI IRB are charged with considering the totality of an institution's performance in considering institutional participation in the protocol.

Participating Institutions that fail to meet the performance goals of accrual, submission of timely and accurate data, adherence to protocol requirements, and compliance with state and federal regulations, may be recommended for a six-month probation period. Such institutions must respond with a corrective action plan and must demonstrate during the probation period that deficiencies have been corrected, as evidenced by the improved performance measures. Participating Institutions that fail to demonstrate significant improvement will be considered by the DF/HCC Sponsor for revocation of participation. A DF/HCC Sponsor and/or the DFCI IRB may terminate a site's participation if it is determined that a site is not fulfilling its responsibilities as described above.
