# Supplementary material for: A prospective trial of treatment de-escalation following neoadjuvant paclitaxel/trastuzumab/pertuzumab in HER2-positive breast cancer
Source: NPJ Breast Cancer. 2022 May 10;8:63. doi: 10.1038/s41523-022-00429-7 (PMC9091255; doi:10.1038/s41523-022-00429-7)
Supplement: Supplementary file 3 — Questionnaire Packet [file 41523_2022_429_MOESM3_ESM.pdf]

DFCI 18-394

DAPHNe: De-escalation to adjuvant antibodies post-pCR to neoadjuvant THP (paclitaxel/trastuzumab/pertuzumab)-a pilot study in HER2-positive breast cancer

Overall Sponsor and PI: Adrienne Gropper Waks, MD

Questionnaire Packet

Version: June 22, 2018

Table of Contents

1. STUDY CONTACT LIST 3

APPENDIX A: QUESTIONNAIRE FOR NON-DE-ESCALATOR PATIENTS 4

APPENDIX B: QUESTIONNAIRE FOR UNPLANNED-DE-ESCALATOR PATIENTS 6

APPENDIX C: STRUCTURED PHYSICIAN REVIEW FORM FOR NON-DE-ESCALATOR PATIENTS 8

APPENDIX D: STRUCTURED PHYSICIAN REVIEW FORM FOR UNPLANNED DE-ESCALATOR PATIENTS 9

APPENDIX E: QUESTIONNAIRE FOR PATIENTS RECEIVING PROTOCOL-CONSISTENT ADJUVANT THERAPY 10

APPENDIX F: ADJUVANT THERAPY PLANNING QUESTIONNAIRE FOR STUDY TEAM 12

STUDY CONTACT LIST

Research Project Manager:
J. Ryan Afable
Dana-Farber Cancer Institute
450 Brookline Ave, LG-GC
Boston, MA 02215
Phone: 617-582-7543
Fax: 617-632-5152
Email: CTOPM@dfci.harvard.edu

Clinical Research Coordinator:
Eileen Wrabel
Dana-Farber Cancer Institute
450 Brookline Ave, Dana 157
Boston, MA 02215
Phone: 617-632-3684
Email: ewrabel@partners.org

For all study-related questions, please email ctopm@dfci.harvard.edu, and ewrabel@partners.org

APPENDIX A: QUESTIONNAIRE FOR NON-DE-ESCALATOR PATIENTS

Date:

Patient Name:

Patient Study ID Number:

These 4 questions ask about your decision-making regarding chemotherapy after surgery.

DEFINITIONS:

- Antibody medicines: Such as trastuzumab (H) and pertuzumab (P); together these medicines are called “HP”. All of the patients on your clinical trial will receive more antibody medicines (typically “HP”) after surgery. Some patients will switch to the antibody medicine T-DM1 after surgery.
- Chemotherapy medicines: Such as paclitaxel (T), or adriamycin (A) plus cyclophosphamide (C) (together these medicines are called “AC”). Not all patients on your clinical trial will receive more chemotherapy medicines after surgery.
- Hormone medicines: Such as tamoxifen or aromatase inhibitor (anastrazole, letrozole, exemestane). Patients with hormone receptor-positive breast cancer will receive hormone medicines after surgery.

*PART I - INSTRUCTIONS: Please **circle ONE number** response for each question.*

1. On a scale from 1 to 5, where 1 = you feel you should not receive more chemotherapy, and 5 = you feel you should receive more chemotherapy, how strongly do **you feel** that you should or should not receive **more chemotherapy** (in addition to HP) after your surgery? (circle one number)

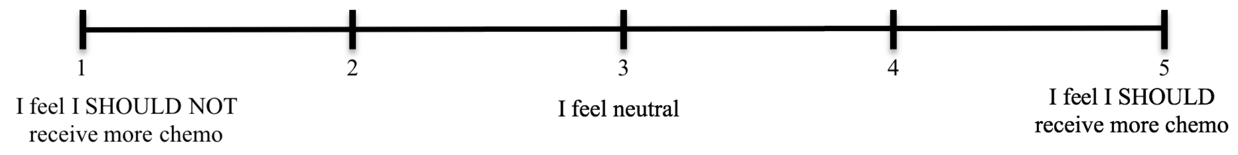

2. On a scale from 1 to 5, where 1 = your doctor feels you should not receive more chemotherapy, and 5 = your doctor feels you should receive more chemotherapy, how strongly does **your doctor feel** that you should or should not receive **more chemotherapy** (in addition to HP) after your surgery? (circle one number)

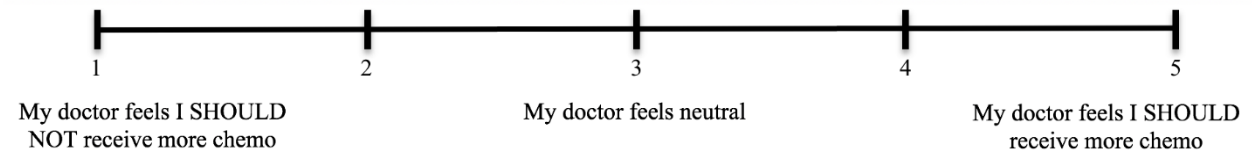

3. On a scale from 1 to 5, where 1 = the chemotherapy I received before surgery went worse than expected, and 5 = the chemotherapy I received before surgery went better than expected, how would you **describe your experience with the chemotherapy you received before surgery**?

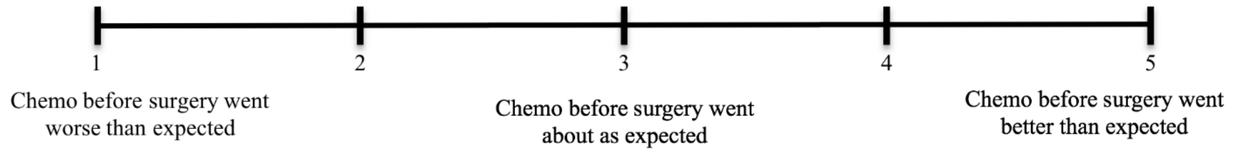

PART II – INSTRUCTIONS: We are interested in the reason(s) that you will be receiving more chemotherapy after surgery. Please ***check ALL that apply.***

4. The reason(s) I am going to receive more chemotherapy after my surgery is/are:

☐ I will not get all my doses of pertuzumab (“P”), so I am getting more chemotherapy instead.

☐ At surgery we found a lot of cancer that was treated and killed in my breast (“treatment effect” in the breast), so we decided that I should get more chemotherapy.

☐ At surgery we found a lot of cancer that was treated and killed in my lymph nodes (“treatment effect” in the lymph nodes), so we decided that I should get more chemotherapy.

☐ Other—*please explain:*____

____

____

APPENDIX B: QUESTIONNAIRE FOR UNPLANNED-DE-ESCALATOR PATIENTS

Date:

Patient Name:

Patient Study ID Number:

These 4 questions ask about your decision-making regarding chemotherapy after surgery.

<u>DEFINITIONS:</u>

- <u>Antibody medicines:</u> Such as trastuzumab (H) and pertzumab (P); together these medicines are called “HP”. All of the patients on your clinical trial will receive more antibody medicines (typically “HP”) after surgery. Some patients will switch to the antibody medicine T-DM1 after surgery.
- <u>Chemotherapy medicines:</u> Such as adriamycin (A) and cyclophosphamide (C); together these medicines are called “AC”. Not all patients on your clinical trial will receive more chemotherapy medicines after surgery.
- <u>Hormone medicines:</u> Such as tamoxifen or aromatase inhibitor (anastrazole, letrozole, exemestane). Patients with hormone receptor-positive breast cancer will receive hormone medicines after surgery.

*<u>PART I - INSTRUCTIONS: Please **circle one number** response for each question.</u>*

1. On a scale from 1 to 5, where 1 = you feel you should not receive more chemotherapy, and 5 = you feel you should receive more chemotherapy, how strongly do **<u>you feel</u>** that you should or should not receive **<u>more chemotherapy</u>** (in addition to HP) after your surgery? (circle one number)

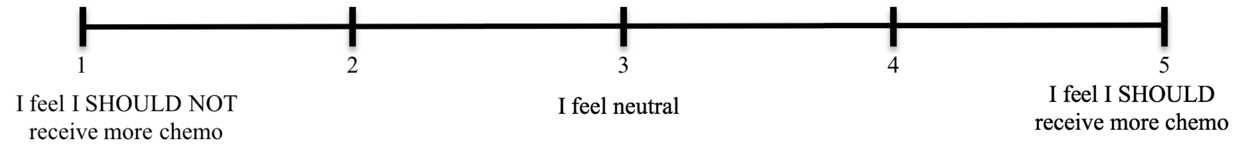

2. On a scale from 1 to 5, where 1 = your doctor feels you should not receive more chemotherapy, and 5 = your doctor feels you should receive more chemotherapy, how strongly does **<u>your doctor feel</u>** that you should or should not receive **<u>more chemotherapy</u>** (in addition to HP) after your surgery? (circle one number)

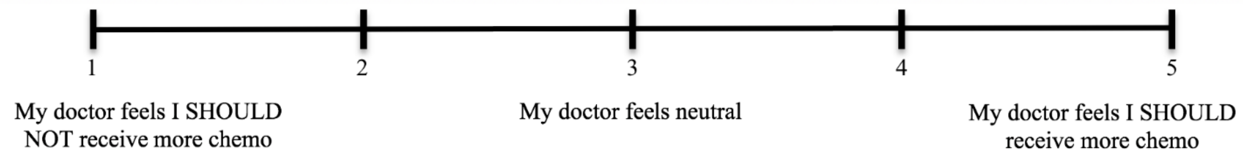

3. On a scale from 1 to 5, where 1 = the chemotherapy I received before surgery went worse than expected, and 5 = the chemotherapy I received before surgery went better than expected, how would you **<u>describe your experience with the chemotherapy you received before surgery</u>**?

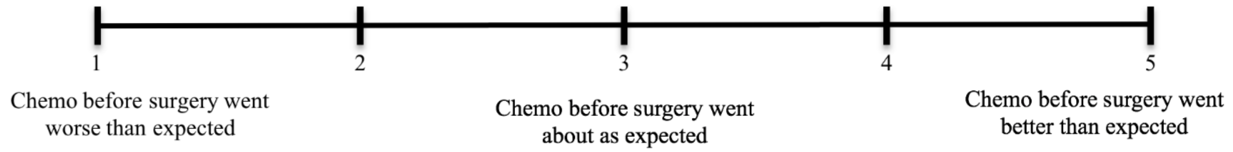

*<u>PART II – We are interested in the reason(s) that you will not be receiving more chemotherapy after surgery. Please **check ALL that apply.**</u>*

4. The reason(s) I am not going to receive more chemotherapy after my surgery is/are:

☐ My cancer already responded very well to the treatment that I got before my surgery, so I am not going to get additional chemotherapy after surgery.

☐ I am going to receive the medicine T-DM1 (also called trastuzumab emtansine, or Kadcyla®) after surgery, so I am not going to get additional chemotherapy after surgery.

☐ Because of my age, my body is not likely to handle more chemotherapy at this point.

☐ Because of illnesses I have other than breast cancer, my body is not likely to handle more chemotherapy at this point.

☐ Because my breast cancer is going to be treated with hormone medicines as well as the “HP” antibody medicines, I do not need more chemotherapy after surgery.

☐ Other—*please explain:*____

____

____

APPENDIX C: STRUCTURED PHYSICIAN REVIEW FORM FOR NON-DE-ESCALATOR PATIENTS

*INSTRUCTIONS: Please **check ALL applicable** box(es) to indicate reason(s) why the patient will receive adjuvant chemotherapy.*

The reason(s) the patient is going to receive more chemotherapy after their surgery is/are:

☐ The patient will not get all doses of pertuzumab ("P"), so they are getting more chemotherapy instead.

☐ There was a lot of cancer that was treated and killed in the breast ("treatment effect" in the breast), so it was decided that the patient should get more chemotherapy.

☐ There was a lot of cancer that was treated and killed in the lymph nodes ("treatment effect" in the lymph nodes), so it was decided that the patient should get more chemotherapy.

☐ Other—*please explain:*____

____

____

APPENDIX D: STRUCTURED PHYSICIAN REVIEW FORM FOR UNPLANNED DE-ESCALATOR PATIENTS

*INSTRUCTIONS: Please **check ALL applicable** box(es) to indicate reason(s) why the patient will not receive adjuvant chemotherapy.*

☐ The patient's cancer already responded very well to the treatment that they got before surgery, so they are not going to get additional chemotherapy after surgery.

☐ The patient is going to receive the medicine T-DM1 (also called trastuzumab emtansine, or Kadcyla®) after surgery, so they are not going to get additional chemotherapy after surgery.

☐ Because of the patient's age, their body is not likely to tolerate more chemotherapy at this point.

☐ Because of illnesses the patient has other than breast cancer, their body is not likely to tolerate more chemotherapy at this point.

☐ Because the patient's breast cancer is going to be treated with hormone medicines as well as the "HP" antibody medicines, the patient does not need more chemotherapy after surgery.

☐ Other—*please explain:*____

____

____

APPENDIX E: QUESTIONNAIRE FOR PATIENTS RECEIVING PROTOCOL-CONSISTENT ADJUVANT THERAPY

Date:

Patient Name:

Patient Study ID Number:

These 4 questions ask about your decision-making regarding chemotherapy after surgery.

DEFINITIONS:

- Antibody medicines: Such as trastuzumab (H) and pertzumab (P); together these medicines are called “HP”. All of the patients on your clinical trial will receive more antibody medicines (typically “HP”) after surgery. Some patients will switch to the antibody medicine T-DM1 after surgery.
- Chemotherapy medicines: Such as adriamycin (A) and cyclophosphamide (C); together these medicines are called “AC”. Not all patients on your clinical trial will receive more chemotherapy medicines after surgery.
- Hormone medicines: Such as tamoxifen or aromatase inhibitor (anastrazole, letrozole, exemestane). Patients with hormone receptor-positive breast cancer will receive hormone medicines after surgery.

*PART I - INSTRUCTIONS: Please **circle one number** response for each question.*

1. On a scale from 1 to 5, where 1 = you feel you should not receive more chemotherapy, and 5 = you feel you should receive more chemotherapy, how strongly do **you feel** that you should or should not receive **more chemotherapy** (in addition to HP) after your surgery? (circle one number)

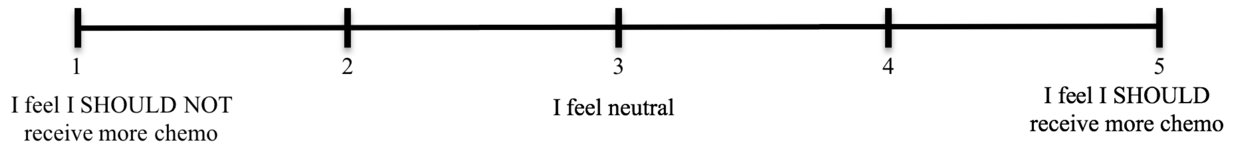

2. On a scale from 1 to 5, where 1 = your doctor feels you should not receive more chemotherapy, and 5 = your doctor feels you should receive more chemotherapy, how strongly does **your doctor feel** that you should or should not receive **more chemotherapy** (in addition to HP) after your surgery? (circle one number)

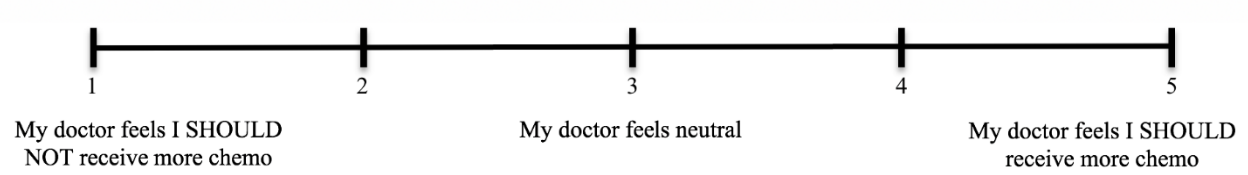

3. On a scale from 1 to 5, where 1 = the chemotherapy I received before surgery went worse than expected, and 5 = the chemotherapy I received before surgery went better than expected, how would you **describe your experience with the chemotherapy you received before surgery**?

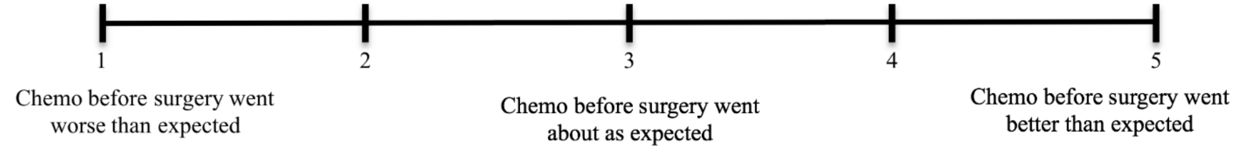

OPTIONAL *PART II – Other than the guidelines of the clinical trial, please list any other* ***reasons that you will or will not be receiving more chemotherapy*** *after surgery.*

NOTE: This question is optional. It is fine to leave it blank.

Please list reasons, if applicable:____

____

____

APPENDIX F: ADJUVANT THERAPY PLANNING QUESTIONNAIRE FOR STUDY TEAM

Subject ID:_____________

Site:__________________

Date of Assessment:_____

1. Did this patient's pathology from final breast surgery demonstrate a pathologic complete response (pCR; defined as ypT0/is ypN0) in breast and evaluated lymph nodes? *(Please circle)*

    a. Yes
    b. No

2. Is adjuvant cytotoxic chemotherapy planned? *(Please circle)*
   Note: cytotoxic chemotherapy does NOT include antibody therapy (i.e. Herceptin [Trastuzumab], Perjeta [Pertuzumab], Kadcyla (T-DM1)), bone modifying agents (bisphosphonates, RANKL inhibitors), or hormonal therapy.

    a. Yes *(Please answer Q3)*
    b. No (*Stop here, do not answer Q3)*

3. If yes to Q2, what regimen is planned? *(Please circle and enter regimen if appropriate)*

    a. Adriamycin/Cytoxan (AC) x 4 cycles
    b. Other, please indicate____________________________________

Investigator Signature: ____

FOR STUDY STAFF:

If Q1=Yes *AND* Q2=Yes, patient is a non-de-escalator. Administer patient questionnaire for non-de-escalator patients.

If Q1=No *AND* Q2=No, patent is an unplanned de-escalator. Administer patient questionnaire for unplanned de-escalator patients.

Otherwise, patient is receiving protocol-consistent adjuvant therapy. Administer patient questionnaire for patients receiving protocol-consistent adjuvant therapy.
