# Supplementary material for: A prospective trial of treatment de-escalation following neoadjuvant paclitaxel/trastuzumab/pertuzumab in HER2-positive breast cancer
Source: NPJ Breast Cancer. 2022 May 10;8:63. doi: 10.1038/s41523-022-00429-7 (PMC9091255; doi:10.1038/s41523-022-00429-7)
Supplement: Supplementary file 4 — NPJ Reporting Summary [file 41523_2022_429_MOESM4_ESM.pdf]

nature portfolio

Corresponding author(s): Eric P. Winer

Last updated by author(s): Nov 15, 2021

Reporting Summary

Nature Portfolio wishes to improve the reproducibility of the work that we publish. This form provides structure for consistency and transparency in reporting. For further information on Nature Portfolio policies, see our Editorial Policies and the Editorial Policy Checklist.

Statistics

For all statistical analyses, confirm that the following items are present in the figure legend, table legend, main text, or Methods section.

n/a	Confirmed	
☐	☒	The exact sample size (n) for each experimental group/condition, given as a discrete number and unit of measurement
☒	☐	A statement on whether measurements were taken from distinct samples or whether the same sample was measured repeatedly
☐	☒	The statistical test(s) used AND whether they are one- or two-sided<br>*Only common tests should be described solely by name; describe more complex techniques in the Methods section.*
☒	☐	A description of all covariates tested
☒	☐	A description of any assumptions or corrections, such as tests of normality and adjustment for multiple comparisons
☒	☐	A full description of the statistical parameters including central tendency (e.g. means) or other basic estimates (e.g. regression coefficient) AND variation (e.g. standard deviation) or associated estimates of uncertainty (e.g. confidence intervals)
☒	☐	For null hypothesis testing, the test statistic (e.g. F, t, r) with confidence intervals, effect sizes, degrees of freedom and P value noted<br>*Give P values as exact values whenever suitable.*
☒	☐	For Bayesian analysis, information on the choice of priors and Markov chain Monte Carlo settings
☒	☐	For hierarchical and complex designs, identification of the appropriate level for tests and full reporting of outcomes
☒	☐	Estimates of effect sizes (e.g. Cohen's d, Pearson's r), indicating how they were calculated

Our web collection on statistics for biologists contains articles on many of the points above.

Software and code

Policy information about availability of computer code

Data collection	No software was used
Data analysis	SAS v9.4 was used for data analysis and R v4.0.2 was used to make figures

For manuscripts utilizing custom algorithms or software that are central to the research but not yet described in published literature, software must be made available to editors and reviewers. We strongly encourage code deposition in a community repository (e.g. GitHub). See the Nature Portfolio guidelines for submitting code & software for further information.

Data

Policy information about availability of data

All manuscripts must include a data availability statement. This statement should provide the following information, where applicable:

- Accession codes, unique identifiers, or web links for publicly available datasets
- A description of any restrictions on data availability
- For clinical datasets or third party data, please ensure that the statement adheres to our policy

The datasets generated during and/or analyzed during the current study are available from the corresponding author on reasonable request.

Field-specific reporting

Please select the one below that is the best fit for your research. If you are not sure, read the appropriate sections before making your selection.

☒ Life sciences ☐ Behavioural & social sciences ☐ Ecological, evolutionary & environmental sciences

For a reference copy of the document with all sections, see nature.com/documents/nr-reporting-summary-flat.pdf

Life sciences study design

All studies must disclose on these points even when the disclosure is negative.

Sample size	Among patients with pCR to THP, de-escalation would be deemed infeasible if the true rate of adherence to HP-only was <80%. With a sample size of 100 patients, the study was designed to have >90% power to reject the null if the true rate of adherence was >95% (binomial exact test; one-sided alpha=0.05).
Data exclusions	Patients who progressed during neoadjuvant THP, withdrew consent to participate, received neoadjuvant therapy in addition to THP, or did not have pCR were not included in the primary analysis (prespecified). Patients who received additional non-THP neoadjuvant therapy were counted as non-pCR.
Replication	No measures were taken to verify the reproducibility of the experimental findings.
Randomization	Allocation was not random and there were no covariates evaluated in this single-arm study.
Blinding	Blinding was not necessary or possible in this single-arm study with a primary endpoint related to feasibility.

Reporting for specific materials, systems and methods

We require information from authors about some types of materials, experimental systems and methods used in many studies. Here, indicate whether each material, system or method listed is relevant to your study. If you are not sure if a list item applies to your research, read the appropriate section before selecting a response.

Materials & experimental systems

n/a	Involved in the study	
☒	☐	Antibodies
☒	☐	Eukaryotic cell lines
☒	☐	Palaeontology and archaeology
☒	☐	Animals and other organisms
☐	☒	Human research participants
☐	☒	Clinical data
☒	☐	Dual use research of concern

Methods

n/a	Involved in the study	
☒	☐	ChIP-seq
☒	☐	Flow cytometry
☒	☐	MRI-based neuroimaging

Human research participants

Policy information about studies involving human research participants

Population characteristics	Eligible patients had clinical anatomic stage II-III HER2+ invasive breast cancer. Patients could have any menopausal or hormone receptor status, and were required to have performance status <1 and adequate organ function at baseline. Patients with baseline cardiac ejection fraction <50% or significant peripheral neuropathy (grade >2 by common terminology criteria for adverse events v4.0) were excluded. Table 1 summarizes patient and tumor characteristics for 98 patients who began treatment on trial. The large majority of patients had clinical anatomic stage II disease (85.7%), and approximately one-third of patients had hormone receptor-negative (HR-) tumors (33.7%). 99% of patients were female. 83.7% of patients were white, 5.1% black, 7.1% Asian, and 4.1% other racial background.
Recruitment	This was a single-arm prospective trial that enrolled patients from 11/2018 to 01/2020 at Dana-Farber/Harvard Cancer Center (DF/HCC; composed of Dana-Farber Cancer Institute [DFCI], Massachusetts General Hospital, and Beth Israel Deaconess Medical Center) and affiliated community satellite practices. Most patients were enrolled at a single tertiary academic cancer center (DFCI) where providers already had familiarity with adjuvant de-escalation trials in HER2+ breast cancer based on participation in prior protocols, which may have impacted their comfort level with this approach and experience presenting it to prospective participants. Offsetting this, approximately one in three enrolled patients were from other centers including community satellite practices.
Ethics oversight	All trial procedures were approved by the DF/HCC institutional review board.

Note that full information on the approval of the study protocol must also be provided in the manuscript.

Clinical data

Policy information about clinical studies

All manuscripts should comply with the ICMJE guidelines for publication of clinical research and a completed CONSORT checklist must be included with all submissions.

Clinical trial registration	NCT03716180
Study protocol	The full protocol is included in Supplementary Material.
Data collection	This trial enrolled patients from 11/2018 to 01/2020 at Dana-Farber/Harvard Cancer Center
Outcomes	The primary objective was to assess adherence to protocol-specified antibody doublet therapy (HP-only) in the adjuvant setting among patients with pCR following neoadjuvant THP. The primary endpoint was receipt of adjuvant cytotoxic chemotherapy, assessed 3 months post-operatively. Among patients with pCR to THP, de-escalation would be deemed infeasible if the true rate of adherence to HP-only was <80%. With a sample size of 100 patients, the study was designed to have >90% power to reject the null if the true rate of adherence was >95% (binomial exact test; one-sided alpha=0.05). Patients who progressed during neoadjuvant THP, withdrew consent to participate, received neoadjuvant therapy in addition to THP, or did not have pCR were not included in the primary analysis (prespecified). Secondary endpoints included event-free survival and overall survival. Patients who received additional non-THP neoadjuvant therapy were counted as non-pCR.
